# Elucidation of the Relationship between CD Cotton Effects and the Absolute Configuration of Sixteen Stereoisomers of Spiroheterocyclic-Lactams

**DOI:** 10.3390/md16070223

**Published:** 2018-06-29

**Authors:** Takeshi Yamada, Tetsuya Kajimoto, Takashi Kikuchi, Reiko Tanaka

**Affiliations:** 1 Department of Medicinal Molecular Chemistry, Osaka University of Pharmaceutical Sciences, 4-20-1, Nasahara, Takatsuki, Osaka 569-1094, Japan; t.kikuchi@gly.oups.ac.jp (T.K.); tanakar@gly.oups.ac.jp (R.T); 2 Medicinal Organic Chemistry Laboratory, College of Pharmaceutical Sciences, Ritsumeikan University, 1-1-1, Nojihigshi, Kusatsu, Shiga 525-8577, Japan; kajimoto@fc.ritsumei.ac.jp

**Keywords:** cephalimysins, *Aspergillus fumigatus*, marine microorganism, *Mugil cephalus*, spiro-heterocyclic γ-lactam, circular dichroism spectroscopy, cytotoxicity

## Abstract

As part of research to search for antitumor agents in fungi originating from marine organisms, cephalimysins E–L were isolated from a culture broth of *Aspergillus fumigatus* that was separated from the marine fish *Mugil cephalus*. One- and two-dimensional nuclear magnetic resonance spectra revealed their planar structures, which are diastereomers of each other. Their absolute stereostructures were established by epimerization at C-8 with acidic methanol, nuclear Overhauser effect spectroscopy (NOESY), and circular dichroism (CD) spectroscopy. These demonstrated the detailed relationships between absolute configuration and CD Cotton effects. Additionally, in the growth inhibition assay against P388, HL-60, L1210, and KB cell lines, some of the fungal metabolites or reaction products exhibited moderate activities.

## 1. Introduction

Our group searched for active antitumor compounds in microorganisms in marine environments [1,2,3,4]. We previously reported the isolation and structure determination of FD-838 and its three diastereomers, referred to as cephalimysins B–D, from a culture broth of the fungal strain *A. fumigatus* OUPS-T106B-5, which lives on the stomach wall of a marine fish, *M. cephalus* [5]. Since FD-838 has three chiral centers, it should have eight stereoisomers. However, naturally occurring spiroheterocyclic-lactams, including FD-838 and cephalimysins B–D isolated from this fungal strain, had the *S* absolute configuration at the methoxy acetal carbon in common. The treatment of FD-838 and its diastereomers with acidic methanol could provide each epimer at the methoxy acetal carbon, and we succeeded in obtaining a set of all the stereoisomers of FD-838. Thus, we unambiguously established the relationship between the absolute configuration and the CD Cotton effects for the spirofuranone-lactam skeleton [5]. Based on the above method, we succeeded in correcting the absolute configuration of pseurotin A_2_ reported by F. Z. Wang and co-workers in 2011 [6,7].

During a further search for novel active antitumor compounds from this fungal strain, we isolated eight new spiroheterocyclic-lactams designated as cephalimysins E–L (**1**–**8**), and their treatment with acidic methanol gave a set of eight unnatural forms (**1**′–**8**′) to afford sixteen stereoisomers. The isolation of many structural or stereoisomers from one source often indicates that their biosynthesis, stereochemistry, and bioactivity are worth investigating. As a part of this research, we hypothesized that **1**–**8** were produced from a plausible precursor with an olefin side chain such as pseurotin A [7], and examined the biosynthetic pathway for an intramolecular annulation reaction using molecular orbital calculations. This examination showed that compounds **1**–**8** were more stably produced from the plausible precursor with an *E*-olefin side chain than one with a *Z*-olefin side chain, and provided a rational explanation why C-2, C-3, and C-13, three chiral centers newly generated by an intramolecular annulation reaction, have all *S* or all *R* absolute configurations [8].

Herein, we report the details of the relationship between the absolute configuration of the above compounds and the CD Cotton effect as well as the elucidation of the relative configurations by nuclear Overhauser effect spectroscopy (NOESY) experiments. Additionally, their cytotoxic activity against murine P388 leukemia, human HL-60 leukemia, murine L1210 leukemia, and human KB epidermoid carcinoma cell lines is described.

## 2. Results and Discussion

**Figure 1:**
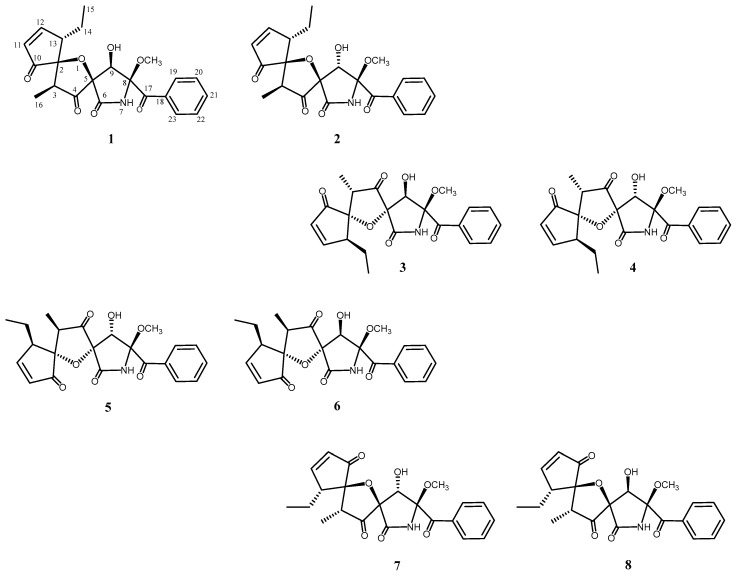
Structures of natural products from *A. fumigatus*.

An ethyl acetate extract of the culture broth of *A. fumigatus* OUPS-T106B-5 was fractionated, employing a stepwise combination of Sephadex LH-20 and silica gel column chromatography, and purification by reverse-phase HPLC afforded cephalimysins E–L (**1**–**8**) (Figure 1) as reported previously [8].

Cephalimysins E–L (**1**–**8**) were assigned the same molecular formula, C_22_H_2__3_NO_7_, based on deductions made from high-resolution fast atom bombardment mass spectral (HRFABMS) data. These ^1^H and ^13^C NMR spectra showed similar features except for differences in the chemical shifts at C-4, C-5, C-8, C-9, and C-10 (Table 1 and Table 2), and HMBC analysis showed that they have the same planar structure. We previously isolated a series of diastereomers, cephalimysins B–D and FD-838, and reported their absolute stereostructures. Compounds **1**–**8** are a new series of diastereomers from the fungal metabolites, which have six chiral centers in their molecules.

**Figure 2:**
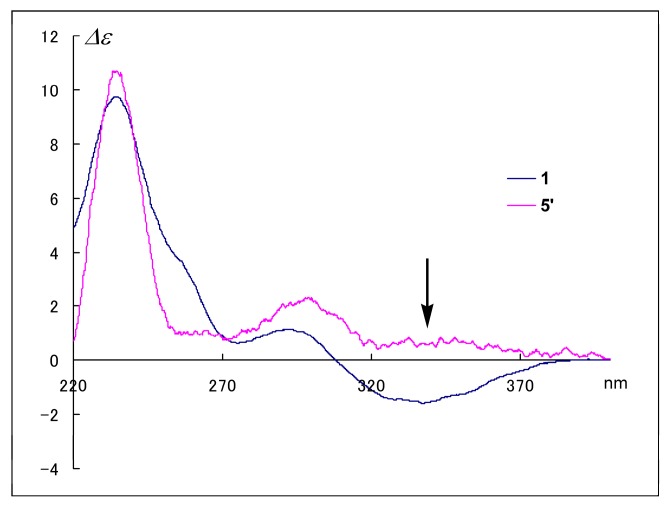
CD spectra of **1** and **5′**.

As a first step toward examining their stereochemistry, we performed epimerization at C-8 with acidic methanol. We previously found that treatment of spiroheterocyclic-lactams with concentrated H_2_SO_4_ in MeOH reversed the absolute configuration at C-8, and the CD Cotton effect of the product showed an opposite sign to that of the reactant at a specific wavelength. Specifically, the CD spectra of cephalimysins B–D and FD-838 with an 8*S* configuration showed a negative Cotton effect at around 320 nm, whereas those of the reaction products, their 8*R* isomers, showed a positive Cotton effect at the same wavelength [5]. In this study, the same acid treatment of **1** gave the 8*R* isomer **5**′ at a constant ratio (7.5%). The Cotton effects in the CD spectrum of **5**′ reversed to positive at around 340 nm as shown by arrow in Figure 2. This result demonstrated that the Cotton effects at around 340 nm assigned the absolute configuration at C-8 in **1**; therefore, the negative Cotton effect (∆*ε*_340_ −1.6) in the CD spectrum of **1** clearly indicated an *S* configuration at C-8 as for the cephalimysins isolated to date [5,7].

In the second step, the relative configuration between H-9 and 8-OCH_3_ was deduced from the coupling constant between H-9 and 9-OH in the ^1^H NMR spectrum. In the ^1^H NMR spectrum of **1**, the coupling constant between H-9 and 9-OH was large (*J* = 12.6 Hz). This was due to a hydrogen bond between 9-OH and 8-OCH_3_, which maintained the conformation of 9-OH; therefore, 9-OH was oriented *cis* to 8-OCH_3_; i.e., **1** had the 9*R* configuration. Hayashi and co-workers reported that the hydrogen bond prevented racemization at C-9 in the last step of the synthesis of synerazol, which was the elimination of the protecting group [9].

**Figure 3:**
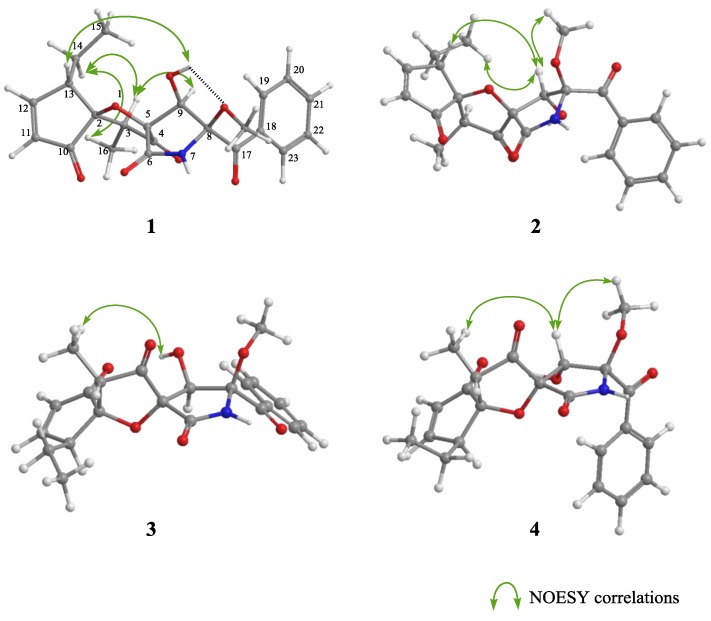
Key NOESY correlations of **1**–**4**.

In NOESY experiments of **1** as a final step, observed NOESY correlations (H-3/H-14 and H-16/H-14) revealed the relative configurations of C-2 and C-13. Additionally, when the H-9/H-3 and 9-OH/H-13 correlations were added, the relative configurations of C-2, C-3, C-5, C-9, and C-13 were deduced as shown in Figure 3. The above three steps demonstrated that **1** possessed the absolute configuration 2*S*,3*S*,5*R*,8*S*,9*R*,13*S*. In NOESY experiments with cephalimysins F–L (**2**–**8**), the observed NOESY correlations (H-3/H-14 and H-16/H-14) were the same as for **1**. Consequently, compounds **2**–**8** all have the same relative configuration of C-2, C-3, and C-13 as that of **1**. Additionally, the negative Cotton effects at around 340 nm in their CD spectra indicated that compounds **2**–**8** all possessed the 8*S* absolute configuration together with the natural spiroheterocyclic γ-lactams to date. If **1**–**8** are the stereoisomers at the two remaining stereogenic centers (C-5 and C-9), eight diastereomers cannot exist; therefore, we guessed that the absolute configurations at C-2, C-3, and C-13 formed two series (2*S*,3*S*,13*S* or 2*R*,3*R*,13*R*). For example, the acid treatment of **1** gave product **5**′ as described above, for which the spectroscopic data were identical to that of **5** except that its [α]_D_ value ([α]_D_ −45.7) had the opposite sign to that of **5** ([α]_D_ +44.0). As expected, the same acid treatment transformed **2**, **3**, and **4** to 8-epimers **6**′, **7**′, and **8**′ as enantiomers of **6**, **7**, and **8**, respectively. On the other hand, the same chemical transformation produced **1**′ from **5**, **2**′ from **6**, **3**′ from **7**, and **4**′ from **8**. CD spectra of the pairs of naturally occurring metabolites **1**–**8** and the reaction products **1**′–**8**′ were observed with inverted signs (see a previous report [8] or Supplementary Materials, Figure S81), and demonstrated that compound **1**–**8** and **1**′–**8**′ were the pairs of enantiomers, respectively. We then examined the absolute configuration at C-5 and C-9 in **2**, **3**, and **4** to match **1**–**8** to the eight assumed diastereomers shown in Figure 1. NOESY correlations for **2** (H-9/H-13, H-15, and 8-OCH_3_) implied that **2** was an epimer of **1** at C-9, and the correlations for **3** (9-OH/H-16) implied that **3** was a epimer of **1** at C-5. Additionally, the NOESY correlations for **4** (H-9/H-16 and H-9/8-OCH_3_) implied that **4** was a stereoisomer of **1** at C-5 and C-9.

**Figure 4:**
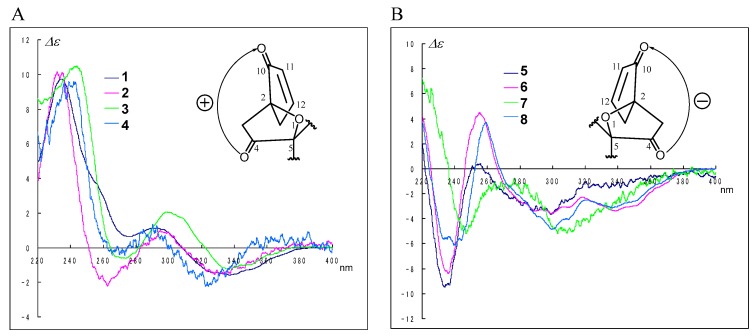
The difference of CD spectra of **1**–**4** twisting clockwise (**A**) and **5**–**8** twisting counterclockwise (**B**) between the carbonyl (C-4) and the enone moiety (C-10–C-12).

The CD spectra of compounds **1**–**8** can be roughly divided into two groups according to the absolute configuration at C-2; i.e., 2*S*,3*S*,13*S* isomers as **1** showed positive Cotton effects (Figure 4A), while 2*R*,3*R*,13*R* isomers as **5** showed negative Cotton effects (Figure 4B) at around 240 (π→π*) and 290 (*n*→π*) nm, respectively. We deduced that the screw sense of chromophores between the carbonyl (C-4) and the enone moiety (C-10–C-12) determined the signs of the CD Cotton effects as shown in Figure 4; therefore, the absolute configurations at C-2, C-3, and C-13 for **2**–**4** and **6**–**8** were revealed; i.e., compounds **2**–**4** were2*S*,3*S*,13*S* isomers, and compounds **6**–**8** were 2*R*,3*R*,13*R* isomers. The above evidence, including the information provided from the transformation to 8-epimers **1**′–**8**′, revealed the absolute stereostructures of **1**–**8**. No additional stereoisomers of **1**–**8** were found despite the six chiral centers in the structure, although further purification was carried out. In a previous study, we studied the biosynthetic pathway for an intramolecular annelation reaction using molecular orbital calculations and concluded that the absolute configurations for C-2, C-3, and C-13 are either all *S* or all *R*.

We successfully isolated sixteen stereoisomers, including eight reaction products, and subsequently deduced the relationship between their stereochemistry and NMR chemical shifts as described below. The absolute configurations at C-2 and C-5 influenced the ^13^C NMR chemical shift of the carbonyl group at C-10; i.e., in 2*S*,5*R* or 2*R*,5*S* isomers (**1**, **2**, **5**, and **6**), the carbonyl group (C-10) and the amido carbonyl group (C-6) are very close; therefore, the carbon signal at C-10 shifted to high field (*δ*_C_ around 204 ppm) by the anisotropic effect. The relationship between the chemical shift and the absolute configuration at C-5 and C-9 is similar to those in cephalimysins B–D and FD-838 [9]; i.e., the ^13^C chemical shift at C-9 for the 5*R*,9*S* isomer (**2** and **7**) is observed in the maximum value (*δ*_C_ around 82 ppm) for the 5*R*,9*S* isomer (**2** and **7**), while that for the 5*R*,9*R* isomer (**1** and **8**) is observed in the minimum value (*δ*_C_ around 70 ppm). These findings, including the CD data, are useful for examining the stereochemistry of spirofuranone-lactams.

As a primary screening for antitumor activity, the inhibitory properties of natural products **1**–**8** and enantiomers **1**′–**8**′ toward cancer cell growth were examined using murine P388 leukemia, human HL-60 leukemia, murine L1210 leukemia, and human KB epidermoid carcinoma cell lines. All compounds except **3**′ and **6**′ exhibited potent or moderate activity against the cell lines (Table 3). Compounds **3** and **6** exhibited the strongest activities (11.1 μM and 7.0 μM, respectively), equal to that of 5-fluorouracil against the KB cell line. Interestingly, **3**′ and **6**′, the enantiomers of **3** and **6**, did not inhibit cell growth. To determine the bioactivity mechanism, molecular target screening for inhibitory effects on histone deacetylases, protein kinases, telomerases, and farnecyltransferases will be needed

## 3. Conclusions

In this study, we established the detailed relationships between absolute configuration and CD Cotton effects for new natural products, cephalimysins E–L (**1**–**8**), isolated a fungal strain separated from a marine fish together with unnatural products **1**′–**8**′. This result agreed well with the consideration derived from the orbital molecular calculation in the previous report [8]. In addition, the growth inhibition assay using P388, HL-60, L1210, and KB cell lines could not give the information of a structure-activity relationship well; however, **3** and **6** against KB cell, and **4**, **5**, **7**′, and **8**′ against L1210 exhibited moderate activities.

## 4. Experimental Section

### 4.1. General Experimental Procedures

NMR spectra were recorded on an Agilent-NMR-vnmrs (Agilent Technologies, Santa Clara, CA, USA) 600 with tetramethylsilane (TMS) as an internal reference. FABMS was recorded using a JEOL JMS-7000 mass spectrometer (JEOL, Tokyo, Japan). IR spectra was recorded on a JASCO FT/IR-680 Plus (Tokyo, Japan). Optical rotations were measured using a JASCO DIP-1000 digital polarimeter (Tokyo, Japan). Silica gel 60 (230–400 mesh, Nacalai Tesque, Inc., Kyoto, Japan) was used for column chromatography with medium pressure. ODS HPLC was run on a JASCO PU-1586 (Tokyo, Japan) equipped with a differential refractometer RI-1531 (Tokyo, Japan) and Cosmosil Packed Column 5C_18_-MSII (25 cm × 20 mm i.d., Nacalai Tesque, Inc., Kyoto, Japan). Analytical TLC was performed on precoated Merck aluminum sheets (DC-Alufolien Kieselgel 60 F254, 0.2 mm, Merck, Darmstadt, Germany) with the solvent system CH_2_Cl_2_–MeOH (19:1) (Nacalai Tesque, Inc., Kyoto, Japan), and compounds were viewed under a UV lamp (AS ONE Co., Ltd., Osaka, Japan) and sprayed with 10% H_2_SO_4_ (Nacalai Tesque, Inc., Kyoto, Japan) followed by heating.

### 4.2. Fungal Material

A strain of *A. fumigatus* was initially isolated from a piece of the marine alga *Undaria pinnatifida* collected at collected in Osaka bay, Japan in May 2015. The fungal strain was identified by Techno Suruga Laboratory Co., Ltd., Shizuoka, Japan. The surface of the marine alga was wiped with EtOH (Nacalai Tesque, Inc., Kyoto, Japan) and its snip applied to the surface of nutrient agar layered in a Petri dish. Serial transfers of one of the resulting colonies provided a pure strain of *A. fumigatus.*

### 4.3. Culturing and Isolation of Metabolites

A strain of *A. fumigatus* was initially isolated from the marine fish *Mugil cephalus* captured in Katsuura Bay, Japan in October 2000. The fish was disinfected with EtOH and its gastrointestinal tract applied to the surface of nutrient agar layered in a Petri dish. Serial transfers of one of the resulting colonies provided a pure strain of *A. fumigatus*. The fungal strain was cultured at 27 °C for 6 weeks in a liquid medium (75 L) containing 1% soluble starch and 0.1% casein in 50% artificial seawater adjusted to pH 7.4. The culture was filtered under suction, and the culture filtrate was extracted three times with EtOAc (Nacalai Tesque, Inc., Kyoto, Japan). The combined extracts were evaporated in vacuo to afford a mixture of crude metabolites (18.8 g) that exhibited cytotoxicity against the P388 cell line (IC_50_ < 1 g/mL). The EtOAc extract was passed through a Sephadex LH-20 (GE Healthcare Japan, Tokyo, Japan) column using CHCl_3_–MeOH (1:1) (Nacalai Tesque, Inc., Kyoto, Japan) as the eluent. The second fraction (14.6 g), exhibiting strong activity, was chromatographed on a silica gel column with a CHCl_3_–MeOH gradient as the eluent to afford Fr. 1 (the 100% CHCl_3_ eluate, 1.2 g) and Fr. 2 (the 1% MeOH in CHCl_3_ eluate, 1.7 g). Fr. 1 was purified by HPLC using MeOH–H_2_O (70:30) as the eluent to afford Fr. 3 (48.3 mg) and Fr. 4 (20.3 mg). Fr. 2 was purified by HPLC using MeOH–H_2_O (70:30) as the eluent to afford Fr. 5 (359.0 mg) and Fr. 6 (263.7 mg). Fr. 3 was purified by ODS HPLC using MeCN–H_2_O (45:55) as the eluent to afford Fr. 7 (2.8 mg) and Fr. 8 (2.1 mg). Fr. 7 and Fr. 8 were further purified by HPLC using MeCN–H_2_O (43:57) as the eluent to afford **4** (1.1 mg, 0.006%) and **7** (0.4 mg, 0.002%), respectively. Fr. 4 was purified by ODS HPLC using MeCN (Nacalai Tesque, Inc., Kyoto, Japan)–H_2_O (45:55) as the eluent to afford Fr. 9 (4.5 mg) and Fr. 10 (3.5 mg). Fr. 9 and Fr. 10 were further purified by HPLC using MeCN–H_2_O (40:60) as the eluent to afford **8** (2.0 mg, 0.011%) and **3** (1.8 mg, 0.009%), respectively. Fr. 5 was purified by ODS HPLC using MeCN–H_2_O (45:55) as the eluent to afford Fr. 11 (17.1 mg) and Fr. 12 (4.5 mg). Fr. 11 was further purified by HPLC using MeCN–H_2_O (30:70) as the eluent to afford **2** (0.8 mg, 0.004%). Fr. 12 was further purified by HPLC using MeCN–H_2_O (40:60) as the eluent to afford **5** (0.3 mg, 0.002). Fr. 6 was purified by ODS HPLC using MeCN–H_2_O (45:55) as the eluent to afford Fr. 13 (4.5 mg) and Fr. 14 (4.0 mg). Fr. 13 was further purified by HPLC using MeCN–H_2_O (38:62) as the eluent to afford **6** (1.4 mg, 0.007%). Fr. 14 was further purified by HPLC using MeCN–H_2_O (40:60) as the eluent to afford **1** (2.2 mg, 0.011%).

**Cephalimysin E** (**1**). Pale yellow oil; [α${]}_{D}^{24}$ +69.1 (*c* 0.16, EtOH); IR (liquid) *ν*_max_ 3342, 2923, 1770, 1729, 1680, 1596, 1490 cm^−1^; UV (EtOH) *λ*_max_ (log *ε*) 249 (3.29), 288 (2.81), 343 (2.44) nm; NMR data, see Table 1 and Table S1 (Supplementary Materials); FABMS *m*/*z* (rel int) 414 ([M + H]^+^, 100.0), 382 ([M − OCH_3_]^+^, 25.2); HRFABMS *m*/*z* 414.1554 [M + H]^+^ (calcd for C_22_H_24_NO_7_ 414.1552); CD (*c* 2.60 × 10^−4^ M, EtOH) *λ*_max_ (∆*ε*) 340 (−1.6), 292 (1.1), 234 (9.7) nm.

**Cephalimysin F** (**2**). Pale yellow oil; [α${]}_{D}^{24}$ +17.1 (*c* 0.10, EtOH); IR (liquid) *ν*_max_ 3274, 2923, 1771, 1731, 1700, 1615, 1597, 1580 cm^−1^; UV (EtOH) *λ*_max_ (log *ε*) 242 (3.93), 284 (3.17), 334 (2.68) nm; NMR data, see Table 1 and Table S2 (Supplementary Materials); FABMS *m*/*z* (rel int) 436 ([M + Na]^+^, 8.2); HRFABMS *m*/*z* 436.1369 [M + Na]^+^ (calcd for C_22_H_2__3_NO_7_Na 436.1372); CD (*c* 1.94 × 10^−4^ M, EtOH) *λ*_max_ (∆*ε*) 331 (−1.6), 295 (1.0), 235 (10.0) nm.

**Cephalimysin G** (**3**). Pale yellow oil; [α${]}_{D}^{24}$ +77.7 (*c* 0.15, EtOH); IR (liquid) *ν*_max_ 3330, 2937, 1769, 1729, 1682, 1625, 1597, 1579 cm^−1^; UV (EtOH) *λ*_max_ (log *ε*) 248 (3.86), 284 (3.43), 334 (3.28) nm; NMR data, see Table 1 and Table S3 (Supplementary Materials); FABMS *m*/*z* (rel int) 436 ([M + Na]^+^, 76.4), 382 ([M − OCH_3_]^+^, 100.0); HRFABMS *m*/*z* 436.1375 [M + Na]^+^ (calcd for C_22_H_23_NO_7_Na 436.1372); CD (*c* 1.97 × 10^−4^ M, EtOH) *λ*_max_ (∆*ε*) 336 (−1.2), 299 (1.8), 241 (10.3) nm.

**Cephalimysin H** (**4**). Pale yellow oil; [α${]}_{D}^{24}$ +110.1 (*c* 0.13, EtOH); IR (liquid) *ν*_max_ 3322, 2925, 1764, 1731, 1695, 1615, 1597, 1579 cm^−1^; UV (EtOH) *λ*_max_ (log *ε*) 241 (4.05), 285 (3.17), 332 (2.71) nm; NMR data, see Table 1 and Table S4 (Supplementary Materials); FABMS *m*/*z* (rel int) 414 ([M + H]^+^, 7.89), 382 ([M − OCH_3_]^+^, 39.85); HRFABMS *m*/*z* 414.1562 [M + H]^+^ (calcd for C_22_H_24_NO_7_ 414.1552); CD (*c* 1.09 × 10^−4^ M, EtOH) *λ*_max_ (∆*ε*) 323 (−2.2), 292 (1.2), 238 (9.5) nm.

**Cephalimysin I** (**5**). Pale yellow oil; [α${]}_{D}^{24}$ −45.7 (*c* 0.15, EtOH); IR (liquid) *ν*_max_ 3333, 2932, 1770, 1732, 1691, 1621, 1597, 1577 cm^−1^; UV (EtOH) *λ*_max_ (log *ε*) 241 (4.08), 283 (3.67), 349 (3.23) nm; NMR data, see Table 2 and Table S5 (Supplementary Materials); FABMS *m*/*z* (rel int) 414 ([M + H]^+^, 100.0), 382 ([M−OCH_3_]^+^, 7.87); HRFABMS *m*/*z* 414.1560 [M + H]^+^ (calcd for C_22_H_24_NO_7_ 414.1552); CD (*c* 1.99 × 10^−4^ M, EtOH) *λ*_max_ (∆*ε*) 335 (−1.6), 299 (−3.6), 234 (−9.5) nm.

**Cephalimysin J** (**6**). Pale yellow oil; [α${]}_{D}^{24}$ −36.9 (*c* 0.16, EtOH); IR (liquid) *ν*_max_ 3334, 2967, 1770, 1731, 1679, 1639, 1596, 1580 cm^−1^; UV (EtOH) *λ*_max_ (log *ε*) 248 (3.76), 289 (3.10), 337 (3.06) nm; NMR data, see Table 2 and Table S6 (Supplementary Materials); FABMS *m*/*z* (rel int) 436 ([M + Na]^+^, 100.0); HRFABMS *m*/*z* 436.1368 [M + Na]^+^ (calcd for C_22_H_23_NO_7_Na 436.1372); CD (*c* 1.22 × 10^−4^ M, EtOH) *λ*_max_ (∆*ε*) 335 (−3.2), 299 (−3.6), 236 (−8.4) nm.

**Cephalimysin K** (**7**). Pale yellow oil; [α${]}_{D}^{24}$ −76.5 (*c* 0.06, EtOH); IR (liquid) *ν*_max_ 3325, 2924, 1767, 1717, 1695, 1616, 1595, 1576 cm^−1^; UV (EtOH) *λ*_max_ (log *ε*) 240(4.07), 286 (3.41), 344 (3.02) nm; NMR data, see Table 2 and Table S7 (Supplementary Materials); FABMS *m*/*z* (rel int) 414 ([M + H]^+^, 6.14), 382 ([M − OCH_3_]^+^, 57.67); HRFABMS *m*/*z* 414.1553 [M + H]^+^ (calcd for C_22_H_24_NO_7_ 414.1552) ; CD (*c* 1.06 × 10^−4^ M, EtOH) *λ*_max_ (∆*ε*) 335 (−3.2), 303 (−5.2), 247 (−5.2) nm.

**Cephalimysin L** (**8**). Pale yellow oil; [α${]}_{D}^{24}$ −18.6 (*c* 0.03, EtOH); IR (liquid) *ν*_max_ 3304, 2938, 1768, 1722, 1698, 1624, 1597, 1579 cm^−1^; UV (EtOH) *λ*_max_ (log *ε*) 246 (3.96), 285 (3.54), 334 (3.53) nm; NMR data, see Table 2 and Table S8 (Supplementary Materials); FABMS *m*/*z* (rel int) 414 ([M + H]^+^, 26.6), 382 ([M − OCH_3_]^+^, 100.0); HRFABMS *m*/*z* 414.1562 [M + H]^+^ (calcd for C_22_H_24_NO_7_ 414.1552); CD (*c* 8.33 × 10^−5^ M, EtOH) *λ*_max_ (∆*ε*) 336 (−3.2), 300 (−4.8), 240 (−6.1) nm.

### 4.4. Epimerization at C-8 in the Natural Occurred Compounds

**Transformation of 1 to 5′:** To a solution of cephalimysin E (**1**) (5.3 mg, 0.013 mmol) in MeOH (1.0 mL) was added concd H_2_SO_4_ (0.01 mL), and the reaction mixture was left at room temperature for 7 h. The mixture was diluted with water and extracted with diethyl ether, and the extract was evaporated under reduced pressure, and then the residue was purified by HPLC using MeCN–H_2_O (38:62) as the eluent to afford **1** (2.7 mg, 50.9%) and **5**′ (0.4 mg, 7.5%).

**5**′**:** pale yellow oil; [α${]}_{D}^{24}$ +44.0 (*c* 0.03, EtOH); CD (*c* 3.23 × 10^−4^ M, EtOH) *λ*_max_ nm (∆*ε*) 336 (0.6), 298 (2.3), 234 (10.7).

**Transformation of 2 to 6′:** Using the same procedure as above with **1**, a solution of cephalimysin F (**2**) (2.5 mg, 0.006 mmol) in MeOH (1.0 mL) was treated with concd H_2_SO_4_ (0.01 mL) and purified by HPLC using MeCN–H_2_O (35:65) as the eluent to afford **2** (0.5 mg, 20.0%) and **6**′ (0.6 mg, 24.0%).

**6**′**:** pale yellow oil; [α${]}_{D}^{24}$ +36.4 (*c* 0.02, EtOH); CD (*c* 3.15 × 10^−5^ M, EtOH) *λ*_max_ nm (∆*ε*) 336 (2.9), 300 (3.8), 236 (8.0).

**Transformation of 3 to 7′:** Using the same procedure as above with **1**, a solution of cephalimysin G (**3**) (6.1 mg, 0.015 mmol) in MeOH (1.0 mL) was treated with concd H_2_SO_4_ (0.01 mL) and purified by HPLC using MeCN–H_2_O (43:57) as the eluent to afford **3** (3.1 mg, 50.8%) and **7**′ (1.5 mg, 24.6%).

**7**′**:** pale yellow oil; [α${]}_{D}^{24}$ +76.3 (*c* 0.11, EtOH); CD (*c* 1.96 × 10^−4^ M, EtOH) *λ*_max_ nm (∆*ε*) 334 (2.1), 305 (3.1), 245 (6.1).

**Transformation of 4 to 8′:** Using the same procedure as above with **1**, a solution of cephalimysin H (**4**) (4.4 mg, 0.011 mmol) in MeOH (1.0 mL) was treated with concd H_2_SO_4_ (0.01 mL) and purified by HPLC using MeCN–H_2_O (43:57) as the eluent to afford **4** (1.4 mg, 31.8%) and **8**′ (0.4 mg, 9.1%).

**8**′**:** pale yellow oil; [α${]}_{D}^{24}$ +19.6 (*c* 0.05, EtOH); CD (*c* 6.47 × 10^−5^ M, EtOH) *λ*_max_ nm (∆*ε*) 337 (2.2), 300 (4.3), 240 (6.4).

**Transformation of 5 to 1′:** Using the same procedure as above with **1**, a solution of cephalimysin I (**5**) (4.5 mg, 0.011 mmol) in MeOH (1.0 mL) was treated with concd H_2_SO_4_ (0.01 mL) and purified by HPLC using MeCN–H_2_O (40:60) as the eluent to afford **5** (1.1 mg, 24.4%) and **1**′ (0.4 mg, 8.9%).

**1**′**:** pale yellow oil; [α${]}_{D}^{24}$ −64.0 (*c* 0.003, EtOH); CD (*c* 3.05 × 10^−4^ M, EtOH) *λ*_max_ nm (∆*ε*) 338 (1.2), 292 (−1.9), 233 (10.2).

**Transformation of 6 to 2′:** Using the same procedure as above with **1**, a solution of cephalimysin J (**6**) (3.7 mg, 0.009 mmol) in MeOH (1.0 mL) was treated with concd H_2_SO_4_ (0.01 mL) and purified by HPLC using MeCN–H_2_O (36: 64) as the eluent to afford **6** (1.8 mg, 48.6%) and **2**′ (0.2 mg, 5.4%).

**2**′**:** pale yellow oil; [α${]}_{D}^{24}$ −17.9 (*c* 0.07, EtOH); CD (*c* 1.15 × 10^−4^ M, EtOH) *λ*_max_ nm (∆*ε*) 332 (1.8), 286 (−2.9), 234 (10.6).

**Transformation of 7 to 3′:** Using the same procedure as above with **1**, a solution of cephalimysin K (**7**) (3.3 mg, 0.008 mmol) in MeOH (1.0 mL) was treated with concd H_2_SO_4_ (0.01 mL) and purified by HPLC using MeCN–H_2_O (43:57) as the eluent to afford **7** (1.0 mg, 30.3%) and **3**′ (0.4 mg, 12.1%).

**3**′**:** pale yellow oil; [α${]}_{D}^{24}$ −78.7 (*c* 0.01, EtOH); CD (*c* 2.57 × 10^−4^ M, EtOH) *λ*_max_ nm (∆*ε*) 335 (1.4), 298 (−2.3), 241 (−9.9).

**Transformation of 8 to 4′:** Using the same procedure as above with **1**, a solution of cephalimysin L (**8**) (7.2 mg, 0.017 mmol) in MeOH (1.0 mL) was treated with concd H_2_SO_4_ (0.01 mL) and purified by HPLC using MeCN–H_2_O (45:55) as the eluent to afford **8** (1.2 mg, 16.7%) and **4**′ (1.1 mg, 15.3%).

**4**′**:** pale yellow oil; [α${]}_{D}^{24}$ −112.0 (*c* 0.13, EtOH); CD (*c* 1.91 × 10^−4^ M, EtOH) *λ*_max_ nm (∆*ε*) 329 (2.0), 289 (−2.4), 238 (10.6).

### 4.5. Assay for Cytotoxicity

Cytotoxic activities of cephalimysins E–L (**1**–**8**), and epimers (**1**′–**8**′) were examined with the 3-(4,5-dimethyl-2-thiazolyl)-2,5-diphenyl-2H-tetrazolium bromide (MTT) method. P388, HL-60, L1210, and KB cells were cultured in RPMI 1640 Medium (10% fetal calf serum) at 37 °C in 5% CO_2_. The test material was dissolved in DMSO to give a concentration of 10 mM, and the solution was diluted with the Essential Medium to yield concentrations of 200, 20, and 2 μM, respectively. Each sample solution (100 μL) was combined with each cell suspension (1 × 10^5^ cells/mL, 100 μL) in the medium to make finally concentrations of 100, 10, and 1 μM, respectively. After incubating at 37 °C for 72 h in 5% CO_2_, grown cells were labeled with 5 mg/mL MTT in phosphate buffered saline (PBS), and the absorbance of formazan dissolved in 20% sodium dodecyl sulfate (SDS) in 0.1 N HCl was measured at 540 nm with a microplate reader. Each absorbance value was expressed as percentage relative to that of the control cell suspension that was prepared without the test substance using the same procedure as that described above. All assays were performed three times, semilogarithmic plots were constructed from the averaged data, and the effective dose of the substance required to inhibit cell growth by 50% (IC_50_) was determined.

## Figures and Tables

**Table 1:** NMR Data for **1**–**4** in CDCl_3_.

Position	1			2			3			4		
	*δ*_H_ *^a^*		*δ*_C_	*δ*_H_ *^a^*		*δ*_C_	*δ*_H_ *^a^*		*δ*_C_	*δ*_H_ *^a^*		*δ*_C_
1												
2			88.7 (s)			87.2 (s)			89.2 (s)			89.8 (s)
3	2.68	q	45.3 (d)	2.87	q	45.8 (d)	2.91	q	46.2 (d)	3.03	q	46.1 (d)
4			207.9 (s)			206.7 (s)			203.4 (s)			204.8 (s)
5			84.1 (s)			87.2 (s)			89.1 (s)			86.1 (s)
6			167.6 (s)			168.7 (s)			168.6 (s)			169.8 (s)
7	7.25	br s		6.65	br s		7.14	br s		7.12	br s	
8			91.6 (s)			87.8 (s)			90.8 (s)			94.8 (s)
9	4.15	d	73.9 (d)	4.54	s	82.1 (d)	4.36	d	76.1 (d)	4.95	d	76.4 (d)
10			204.0 (s)			204.5 (s)			211.0 (s)			209.0 (s)
11	6.28	dd	131.7 (d)	6.25	dd	131.5 (d)	6.23	dd	131.0 (d)	6.20	dd	130.9 (d)
12	7.78	dd	164.1 (d)	7.76	dd	164.7 (d)	7.84	dd	168.0 (d)	7.81	dd	167.1 (d)
13	3.10	ddq	52.2 (d)	3.09	ddt	51.9 (d)	3.12	ddt	51.1 (d)	3.16	ddt	50.2 (d)
14A	1.41	ddq	22.2 (t)	1.45	ddq	22.4 (t)	1.52	ddq	21.3 (t)	1.56	ddq	21.3 (t)
14B	1.91	dqd		1.90	dqd		1.97	dqd		2.00	d quint	
15	1.22	t	12.2 (q)	1.21	t	12.2 (q)	1.25	t	12.1 (q)	1.26	t	12.1 (q)
16	1.09	d	9.1 (q)	1.06	d	8.8 (q)	1.00	d	9.2 (q)	1.00	d	9.1 (q)
17			194.0 (s)			197.0 (s)			194.9 (s)			193.5 (s)
18			133.1 (s)			134.3 (s)			132.4 (s)			133.9 (s)
19	8.30	d	130.6 (d)	8.07	d	129.0 (d)	8.49	d	131.2 (d)	8.27	d	129.8 (d)
20	7.48	t	128.5 (d)	7.50	t	128.9 (d)	7.47	t	128.4 (d)	7.49	t	128.6 (d)
21	7.63	t	134.4 (d)	7.63	t	134.0 (d)	7.62	t	134.4 (d)	7.61	t	133.8 (d)
22	7.48	t	128.5 (d)	7.50	t	128.9 (d)	7.47	t	128.4 (d)	7.49	t	128.6 (d)
23	8.30	d	130.6 (d)	8.07	d	129.0 (d)	8.49	d	131.2 (d)	8.27	d	129.8 (d)
8-OCH_3_	3.24	s	51.3 (q)	3.43	s	51.7 (q)	3.37	s	51.1 (q)	3.25	s	51.2 (q)
9-OH	3.53	d		5.05	br s		5.51	d		2.62	d	

*a* ^1^H chemical shift values (*δ* ppm from SiMe_4_) followed by multiplicity.

**Table 2:** NMR Data for **5**–**8** in CDCl_3._

Position	5			6			7			8		
	*δ*_H_ *^a^*		*δ*_C_	*δ*_H_ *^a^*		*δ*_C_	*δ*_H_ *^a^*		*δ*_C_	*δ*_H_ *^a^*		*δ*_C_
1												
2			87.0 (s)			86.3 (s)			88.5 (s)			89.4 (s)
3	2.88	q	44.0 (d)	2.90	q	45.6 (d)	3.09	q	47.2 (d)	3.19	q	46.2 (d)
4			202.7 (s)			205.0 (s)			209.4 (s)			205.8 (s)
5			85.1 (s)			88.9 (s)			83.0 (s)			85.0 (s)
6			168.7 (s)			167.7 (s)			170.5 (s)			169.4 (s)
7	7.32	br s		7.28	br s		7.14	br s		6.98	br s	
8			92.8 (s)			88.0 (s)			96.7 (s)			93.5 (s)
9	4.44	s	78.2 (d)	4.19	d	76.5 (d)	4.99	d	81.8 (d)	4.62	d	70.1 (d)
10			203.4 (s)			204.7 (s)			209.0 (s)			211.9 (s)
11	6.29	dd	131.6 (d)	6.27	dd	131.5 (d)	6.25	dd	131.1 (d)	6.27	dd	131.1 (d)
12	7.74	dd	164.4 (d)	7.74	dd	164.9 (d)	7.84	dd	167.4 (d)	7.84	dd	167.7 (d)
13	2.95	ddt	51.7 (d)	2.97	ddt	51.8 (d)	3.14	ddt	50.1 (d)	3.20	ddt	50.5 (d)
14A	1.54	ddq	22.3 (t)	1.43	ddq	22.4 (t)	1.54	ddq	21.2 (t)	1.56	ddq	21.0 (t)
14B	1.93	dqd		1.89	dqd		1.93	dqd		1.96	dqd	
15	1.14	t	12.3 (q)	1.15	t	12.2 (q)	1.22	t	12.2 (q)	1.25	t	12.1 (q)
16	1.10	d	8.7 (q)	1.06	d	8.6 (q)	0.97	d	8.6 (q)	0.96	d	8.8 (q)
17			192.2 (s)			194.1 (s)			192.7 (s)			194.1 (s)
18			133.9 (s)			132.9 (s)			133.9 (s)			132.7 (s)
19	8.20	d	129.5 (d)	8.18	d	130.2 (d)	8.25	d	129.7 (d)	8.48	d	131.2 (d)
20	7.49	t	128.7 (d)	7.48	t	128.7 (d)	7.48	t	128.6 (d)	7.49	t	128.4 (d)
21	7.62	t	134.0 (d)	7.63	t	134.3 (d)	7.61	t	133.8 (d)	7.63	t	134.4 (d)
22	7.49	t	128.7 (d)	7.48	t	128.7 (d)	7.48	t	128.6 (d)	7.49	t	128.4 (d)
23	8.20	d	129.5 (d)	8.18	d	130.2 (d)	8.25	d	129.7 (d)	8.48	d	131.2 (d)
8-OCH_3_	3.25	s	50.9 (q)	3.38	s	51.2 (q)	3.20	s	51.2 (q)	3.33	s	51.8 (q)
9-OH	2.96	br s		3.55	d		4.44	d		4.52	d	

*^a^* As in Table 1.

**Table 3:** Cytotoxicity assay against P388, HL-60, L1210, and KB cells.

Compounds	Cell line P388	Cell line HL-60	Cell line L1210	Cell line KB
	IC_50_ (µM) *^a^*	IC_50_ (µM) *^a^*	IC_50_ (µM) *^a^*	IC_50_ (µM) *^a^*
**1**	58.5	57.9	60.5	33.9
**2**	56.9	55.7	62.2	60.5
**3**	26.6	15.7	58.1	11.1
**4**	55.2	52.5	12.8	35.1
**5**	69.0	55.2	14.3	31.5
**6**	51.2	50.8	57.6	7.0
**7**	56.9	53.9	22.5	53.3
**8**	57.5	58.1	60.5	52.1
**1′**	56.4	54.9	59.3	118.6
**2′**	53.5	67.8	55.9	198.5
**3′**	>200	>200	64.4	>200
**4′**	50.8	53.1	20.5	42.4
**5′**	54.5	55.7	20.3	53.0
**6′**	>200	>200	92.0	>200
**7′**	52.1	52.9	14.5	26.6
**8′**	53.3	54.5	12.3	52.3
5-fluorouracil *^b^*	2.8	3.2	2.0	8.5

*^a^* DMSO was used as vehicle; *^b^* Positive control.

## Supplementary Materials

The following are available online at http://www.mdpi.com/1660-3397/16/7/223/s1, Table S1: Spectral data including 2D NMR data for **1**, Table S2: Spectral data including 2D NMR data for **2**, Table S3: Spectral data including 2D NMR data for **3**, Table S4: Spectral data including 2D NMR data for **4**, Table S5: Spectral data including 2D NMR data for **5**, Table S6: Spectral data including 2D NMR data for **6**, Table S7: Spectral data including 2D NMR data for **7**, Table S8: Spectral data including 2D NMR data for **8**, Figure S1: ^1^H and ^13^C NMR spectrum of **1** in CDCl_3_, Figure S2: ^1^H-^1^H COSY of **1**, Figure S3: NOESY of **1**, Figure S4: HMQC of **1**, Figure S5: HMBC of **1**, Figure S6: IR Spectrum of **1**, Figure S7: FABMS of **1**, Figure S8: ^1^H and ^13^C NMR spectrum of **2** in CDCl_3_, Figure S9: ^1^H-^1^H COSY of **2**, Figure S10: NOESY of **2**, Figure S11: HMQC of **2**, Figure S12: HMBC of **2**, Figure S13: IR Spectrum of **2**, Figure S14: FABMS of **2**, Figure S15: ^1^H and ^13^C NMR spectrum of **3** in CDCl_3_, Figure S16: ^1^H-^1^H COSY of **3**, Figure S17: NOESY of **3**, Figure S18: HMQC of **3**, Figure S19: HMBC of **3**, Figure S20: IR Spectrum of **3**, Figure S21: FABMS of **3**, Figure S22: ^1^H and ^13^C NMR spectrum of **4** in CDCl_3_, Figure S23: ^1^H-^1^H COSY of **4**, Figure S24: NOESY of **4**, Figure S25: HMQC of **4**, Figure S26: HMBC of **4**, Figure S27: IR Spectrum of **4**, Figure S28: FABMS of **4**, Figure S29: ^1^H and ^13^C NMR spectrum of **5** in CDCl_3_, Figure S30: ^1^H-^1^H COSY of **5**, Figure S31: NOESY of **5**, Figure S32: HMQC of **5**, Figure S33: HMBC of **5**, Figure S34: IR Spectrum of **5**, Figure S35: FABMS of **5**, Figure S36: ^1^H and ^13^C NMR spectrum of **6** in CDCl_3_, Figure S37: ^1^H-^1^H COSY of **6**, Figure S38: NOESY of **6**, Figure S39: HMQC of **6**, Figure S40: HMBC of **6**, Figure S41: IR Spectrum of **6**, Figure S42: FABMS of **6**, Figure S43: ^1^H and ^13^C NMR spectrum of **7** in CDCl_3_, Figure S44: ^1^H-^1^H COSY of **7**, Figure S45: NOESY of **7**, Figure S46: HMQC of **7**, Figure S47: HMBC of **7**, Figure S48: IR Spectrum of **7**, Figure S49: FABMS of **7**, Figure S50: ^1^H and ^13^C NMR spectrum of **8** in CDCl_3_, Figure S51: ^1^H-^1^H COSY of **8**, Figure S52: NOESY of **8**, Figure S53: HMQC of **8**, Figure S54: HMBC of **8**, Figure S55: IR Spectrum of **8**, Figure S56: FABMS of **8**, Figure S57: ^1^H NMR spectrum of **1**′ in CDCl_3_, Figure S58: ^1^H NMR spectrum of **2**′ in CDCl_3_, Figure S59: ^1^H NMR spectrum of **3**′ in CDCl_3_, Figure S60: ^1^H NMR spectrum of **4**′ in CDCl_3_, Figure S61: ^1^H NMR spectrum of **5**′ in CDCl_3_, Figure S62: ^1^H NMR spectrum of **6**′ in CDCl_3_, Figure S63: ^1^H NMR spectrum of **7**′ in CDCl_3_, Figure S64: ^1^H NMR spectrum of **8**′ in CDCl_3_, Figure S65: HPLC purification of Compound **1**, Figure S66: HPLC purification of Compound **2**, Figure S67: HPLC purification of Compound **3**, Figure S68: HPLC purification of Compound **4**, Figure S69: HPLC purification of Compound **5**, Figure S70: HPLC purification of Compound **6**, Figure S71: HPLC purification of Compound **7**, Figure S72: HPLC purification of Compound **8**, Figure S73: HPLC purification of Compound **5**′, Figure S74: HPLC purification of Compound **6**′, Figure S75: HPLC purification of Compound **7**′, Figure S76: HPLC purification of Compound **8**′, Figure S77: HPLC purification of Compound **1**′, Figure S78: HPLC purification of Compound **2**′, Figure S79: HPLC purification of Compound **3**′, Figure S80: HPLC purification of Compound **4**′, Figure S81: The CD spectra of the 16 stereoisomers **1**–**8** and **1**′–**8**′, symmetrical Cotton effects between enantiomers.
