# Supplementary material for: Elucidation of the Relationship between CD Cotton Effects and the Absolute Configuration of Sixteen Stereoisomers of Spiroheterocyclic-Lactams
Source: Mar Drugs. 2018 Jun 29;16(7):223. doi: 10.3390/md16070223 (PMC6071047; doi:10.3390/md16070223)

Supplementary Materials

Elucidation of the relationship between CD Cotton effects and the absolute configuration of sixteen stereoisomers of spiroheterocyclic-lactams

Takeshi Yamada, Tetsuya Kajimoto, Takashi Kikuchi and Reiko Tanaka

The Table of Contents

**Table S1**	**Spectral data including 2D NMR data for 1**	**3**
**Table S2**	**Spectral data including 2D NMR data for 2**	**4**
**Table S3**	**Spectral data including 2D NMR data for 3**	**5**
**Table S4**	**Spectral data including 2D NMR data for 4**	**6**
**Table S5**	**Spectral data including 2D NMR data for 5**	**7**
**Table S6**	**Spectral data including 2D NMR data for 6**	**8**
**Table S7**	**Spectral data including 2D NMR data for 7**	**9**
**Table S8**	**Spectral data including 2D NMR data for 8**	**10**
**Figure S1**	**^{1}H and ^{13}C NMR spectrum of 1 in $CDCl_3$**	**11**
**Figure S2**	**^{1}H-^{1}H COSY of 1**	**12**
**Figure S3**	**NOESY of 1**	**13**
**Figure S4**	**HMQC of 1**	**15**
**Figure S5**	**HMBC of 1**	**16**
**Figure S6**	**IR Spectrum of 1**	**17**
**Figure S7**	**FABMS of 1**	**18**
**Figure S8**	**^{1}H and ^{13}C NMR spectrum of 2 in $CDCl_3$**	**19**
**Figure S9**	**^{1}H-^{1}H COSY of 2**	**20**
**Figure S10**	**NOESY of 2**	**21**
**Figure S11**	**HMQC of 2**	**22**
**Figure S12**	**HMBC of 2**	**23**
**Figure S13**	**IR Spectrum of 2**	**24**
**Figure S14**	**FABMS of 2**	**25**
**Figure S15**	**^{1}H and ^{13}C NMR spectrum of 3 in $CDCl_3$**	**26**
**Figure S16**	**^{1}H-^{1}H COSY of 3**	**27**
**Figure S17**	**NOESY of 3**	**28**
**Figure S18**	**HMQC of 3**	**30**

**Figure S19**	**HMBC of 3**	**31**
**Figure S20**	**IR Spectrum of 3**	**32**
**Figure S21**	**FABMS of 3**	**33**
**Figure S22**	**^{1}H and ^{13}C NMR spectrum of 4 in $CDCl_3$**	**34**
**Figure S23**	**^{1}H-^{1}H COSY of 4**	**35**
**Figure S24**	**NOESY of 4**	**36**
**Figure S25**	**HMQC of 4**	**37**
**Figure S26**	**HMBC of 4**	**38**
**Figure S27**	**IR Spectrum of 4**	**39**
**Figure S28**	**FABMS of 4**	**40**
**Figure S29**	**^{1}H and ^{13}C NMR spectrum of 5 in $CDCl_3$**	**41**
**Figure S30**	**^{1}H-^{1}H COSY of 5**	**42**
**Figure S31**	**NOESY of 5**	**43**
**Figure S32**	**HMQC of 5**	**44**
**Figure S33**	**HMBC of 5**	**45**
**Figure S34**	**IR Spectrum of 5**	**46**
**Figure S35**	**FABMS of 5**	**47**
**Figure S36**	**^{1}H and ^{13}C NMR spectrum of 6 in $CDCl_3$**	**48**
**Figure S37**	**^{1}H-^{1}H COSY of 6**	**49**
**Figure S38**	**NOESY of 6**	**50**
**Figure S39**	**HMQC of 6**	**51**
**Figure S40**	**HMBC of 6**	**52**
**Figure S41**	**IR Spectrum of 6**	**53**
**Figure S42**	**FABMS of 6**	**54**
**Figure S43**	**^{1}H and ^{13}C NMR spectrum of 7 in $CDCl_3$**	**55**
**Figure S44**	**^{1}H-^{1}H COSY of 7**	**56**
**Figure S45**	**NOESY of 7**	**57**
**Figure S46**	**HMQC of 7**	**58**
**Figure S47**	**HMBC of 7**	**59**
**Figure S48**	**IR Spectrum of 7**	**60**
**Figure S49**	**FABMS of 7**	**61**
**Figure S50**	**^{1}H and ^{13}C NMR spectrum of 8 in $CDCl_3$**	**62**
**Figure S51**	**^{1}H-^{1}H COSY of 8**	**63**
**Figure S52**	**NOESY of 8**	**64**
**Figure S53**	**HMQC of 8**	**65**
**Figure S54**	**HMBC of 8**	**66**

Figure S55 **IR Spectrum of 8** **67**

Figure S56 **FABMS of 8** **68**

Figure S57 **^{1}H NMR spectrum of 1' in $CDCl_3$** **69**

Figure S58 **^{1}H NMR spectrum of 2' in $CDCl_3$** **69**

Figure S59 **^{1}H NMR spectrum of 3' in $CDCl_3$** **70**

Figure S60 **^{1}H NMR spectrum of 4' in $CDCl_3$** **70**

Figure S61 **^{1}H NMR spectrum of 5' in $CDCl_3$** **71**

Figure S62 **^{1}H NMR spectrum of 6' in $CDCl_3$** **71**

Figure S63 **^{1}H NMR spectrum of 7'in $CDCl_3$** **72**

Figure S64 **^{1}H NMR spectrum of 8' in $CDCl_3$** **72**

Figure S65 **HPLC purification of Compound 1** **73**

Figure S66 **HPLC purification of Compound 2** **74**

Figure S67 **HPLC purification of Compound 3** **75**

Figure S68 **HPLC purification of Compound 4** **76**

Figure S69 **HPLC purification of Compound 5** **77**

Figure S70 **HPLC purification of Compound 6** **78**

Figure S71 **HPLC purification of Compound 7** **79**

Figure S72 **HPLC purification of Compound 8** **80**

Figure S73 **HPLC purification of Compound 5'** **81**

Figure S74 **HPLC purification of Compound 6'** **82**

Figure S75 **HPLC purification of Compound 7'** **83**

Figure S76 **HPLC purification of Compound 8'** **84**

Figure S77 **HPLC purification of Compound 1'** **85**

Figure S78 **HPLC purification of Compound 2'** **86**

Figure S79 **HPLC purification of Compound 3'** **87**

Figure S80 **HPLC purification of Compound 4'** **88**

Figure S81 **the CD spectra of the 16 stereoisomers 1–8 and 1′–8′,**
symmetrical Cotton effects between enantiomers **89**

Table S1 NMR spectral data of **1** in $CDCl_3$

Position	δ_H[a]		J/Hz	^{1}H-^{1}H COSY	NOE	δ_C		HMBC (C)[b]
1								
2						88.7	(s)	
3	2.68	q	6.9 (16)	16	9, 14, 15, 16	45.3	(d)	2, 4, 10, 13, 16
4						207.9	(s)	
5						84.1	(s)	
6						167.6	(s)	5
7	7.25	br s			19, 23, 8-OCH_3			
8						91.6	(s)	
9	4.15	d	12.6 (9-OH)	9-OH	3, 19, 23, 9-OH	73.9	(d)	4, 17
10						204.0	(s)	
11	6.28	dd	6.2 (12), 2.0 (13)	12	12, 16	131.7	(d)	2, 10, 12, 13
12	7.78	dd	6.2 (11), 2.0 (13)	11, 13	11, 13, 14, 15	164.1	(d)	2, 10, 11, 13
13	3.10	ddt	12.8 (14A), 5.0 (14B), 2.0 (11, 12)	11, 12, 14	12, 14, 15, 9-OH	52.2	(d)	
14A	1.41	ddq	14.0 (14B), 12.8 (13), 7.3 (15)	13, 15	3, 12, 13, 15, 16	22.2	(t)	12, 13, 15
14B	1.91	dqd	14.0 (14A), 7.3 (15), 5.0 (14)					
15	1.22	t	7.3 (14)	14	3, 12, 13, 14	12.2	(q)	13, 14
16	1.09	d	6.9 (3)	3	3, 11, 14	9.1	(q)	2, 3, 4
17						194.0	(s)	
18						133.1	(s)	
19	8.30	d	7.3 (20)	20	7, 9, 20, 8-CH_3, 9-OH	130.6	(d)	17, 21
20	7.48	t	7.3 (19, 21)	19, 21	19, 21	128.5	(d)	18, 19, 21
21	7.63	t	7.3 (20, 22)	20, 22	20, 22	134.4	(d)	19
22	7.48	t	7.3 (21, 23)	21, 23	21, 23	128.5	(d)	18, 21, 23
23	8.30	d	7.3 (22)	22	7, 9, 22, 8-CH_3, 9-OH	130.6	(d)	17, 21
8-OCH_3	3.24	s			7, 19, 23	51.3	(q)	8
9-OH	3.53	d	12.6 (9)	9	9, 13, 19, 23			9

a ^{1}H chemical shift values (δ ppm from $SiMe_4$) followed by multiplicity and then the coupling constants (J/Hz). Figures in parentheses indicate the proton coupling with that position. *b* Long range ^{1}H-^{13}C correlations from H to C observed in the HMBC experiment.

Table S2 NMR spectral data of **2** in $CDCl_3$

Position	δ_H[a]		J/Hz	^1H-^1H COSY	NOE	δ_C		HMBC (C)[b]
1								
2						87.2	(s)	
3	2.87	q	6.9 (16)	16	9, 14, 16	45.8	(d)	2, 4, 10, 13, 16
4						206.7	(s)	
5						87.2	(s)	
6						168.7	(s)	
7	6.65	br s						
8						87.8	(s)	
9	4.54	s			3, 15, 19, 23, 8-OH_3	82.1	(d)	8
10						204.5	(s)	
11	6.25	dd	6.2 (12), 2.0 (13)	12, 13	12, 13	131.5	(d)	2, 10, 13
12	7.76	dd	6.2 (11), 2.0 (13)	11, 13	11, 13, 15	164.7	(d)	10, 11, 13
13	3.09	ddt	10.8 (14A), 6.2 (14B), 2.0 (11, 12)	11, 12, 14	12, 14, 15	51.9	(d)	
14A	1.45	ddq	14.0 (14B), 10.8 (13), 7.3 (15)	13, 15	3, 13, 15, 16	22.4	(t)	13, 15
14B	1.90	dqd	14.0 (14A), 7.3 (15), 6.2 (13)					
15	1.21	t	7.3 (14)	14	9, 12, 13, 14	12.2	(q)	13, 14
16	1.06	d	6.9 (3)	3	3, 11, 14	8.8	(q)	2, 3, 4
17						197.0	(s)	
18						134.3	(s)	
19	8.07	d	7.3 (20)	20	9, 20, 8-OCH_3	129.0	(d)	17, 21
20	7.50	t	7.3 (19, 21)	19, 21	19, 21	128.9	(d)	18, 19, 21
21	7.63	t	7.3 (20, 22)	20, 22	20, 22	134.0	(d)	19
22	7.50	t	7.3 (21, 23)	21, 23	21, 23	128.9	(d)	18, 21, 23
23	8.07	d	7.3 (22)	22	9, 22, 8-OCH_3	129.0	(d)	17, 21
8-OCH_3	3.43	s			9, 19, 23	51.7	(q)	8
9-OH	5.05	br s						

a, b As in Table S1

Table S3 NMR spectral data of **3** in $CDCl_3$

Position	δ_H[a]		J/Hz	^1H-^1H COSY	NOE	δ_C		HMBC (C)[b]
1								
2						89.2	(s)	
3	2.91	q	6.9 (16)	16	14, 15, 16	46.2	(d)	2, 4, 10, 13, 16
4						203.4	(s)	
5						89.1	(s)	
6						168.6	(s)	
7	7.14	br s			9, 19, 23, 8-OCH_3			
8						90.8	(s)	
9	4.36	d	3.2 (9-OH)	9-OH	7, 19, 23, 8-OCH_3, 9-OH	76.1	(d)	4, 17
10						211.0	(s)	
11	6.23	dd	6.2 (12), 2.0 (13)	12	12, 16	131.0	(d)	2, 10, 12, 13
12	7.83	dd	6.2 (11), 2.0 (13)	11, 13	11, 13, 15, 16	168.0	(d)	2, 10, 11, 13
13	3.12	ddt	11.8 (14A), 6.5 (14B), 2.0 (11, 12)	11, 12, 14	12, 14, 15	51.1	(d)	2, 14
14A	1.52	ddq	14.0 (14B), 11.8 (13), 7.3 (15)	13, 15	3, 12, 13, 15, 16	21.3	(t)	12, 13, 15
14B	1.97	dqd	14.0 (14A), 7.3 (15), 6.5 (13)					
15	1.25	t	7.3 (14)	14	3, 12, 13, 14, 16	12.1	(q)	13, 14
16	1.00	d	6.9 (3)	3	3, 11, 12, 14, 15, 9-OH	9.2	(q)	2, 3, 4
17						194.9	(s)	
18						132.4	(s)	
19	8.49	d	7.3 (20)	20	7, 9, 20, 8-OCH_3, 9-OH	131.2	(d)	17, 21
20	7.47	t	7.3 (19, 21)	19, 21	19, 21	128.4	(d)	19, 21, 23
21	7.62	t	7.3 (20, 22)	20, 22	20, 22	134.4	(d)	19
22	7.47	t	7.3 (21, 23)	21, 23	21, 23	128.4	(d)	19, 21, 23
23	8.49	d	7.3 (22)	22	7, 9, 22, 8-OCH_3, 9-OH	131.2	(d)	17, 21
8-OCH_3	3.37	s			7, 9, 19, 23, 9-OH	51.7	(q)	8
9-OH	5.51	d	3.2 (9)	9	9, 16, 19, 23, 8-OCH_3			8, 9

a, b As in Table S1

Table S4 NMR spectral data of **4** in $CDCl_3$

Position	δ_H[a]		J/Hz	^1H-^1H COSY	NOE	δ_C		HMBC (C)[b]
1								
2						89.8	(s)	
3	3.03	q	6.9 (16)	16	14, 15, 16	46.1	(d)	2, 4, 10, 13, 16
4						204.8	(s)	
5						86.1	(s)	
6						169.8	(s)	
7	7.12	br s			8-OCH_3			5, 9
8						94.8	(s)	
9	4.95	d	1.8 (9-OH)	9-OH	16, 19, 23, 8-OCH_3, 9-OH	76.4	(d)	4, 6, 8
10						209.0	(s)	
11	6.20	dd	6.2 (12), 2.0 (13)	12, 13	12, 16	130.9	(d)	2, 10, 12, 13
12	7.81	dd	6.2 (11), 2.0 (13)	11, 13	11, 13, 14, 15	167.1	(d)	2, 10, 11, 13
13	3.16	ddt	10.0 (14A), 7.3 (14B), 2.0 (11, 12)	11, 12, 14	12, 14, 15	50.2	(d)	14
14A	1.56	ddq	14.0 (14B), 10.0 (13), 7.3 (15)	13, 15	3, 12, 13, 15, 16	21.3	(t)	2, 12, 13, 15
14B	2.00	d quint	14.0 (14A), 7.3 (13, 15)					
15	1.26	t	7.3 (14)	14	3, 12, 13, 14	12.1	(q)	13, 14
16	1.00	d	6.9 (3)	3	3, 9, 11, 14	9.1	(q)	2, 3, 4
17						193.5	(s)	
18						133.9	(s)	
19	8.27	d	7.3 (20)	20	9, 20, 8-OCH_3	129.8	(d)	17, 21
20	7.49	t	7.3 (19, 21)	19, 21	19, 21	128.6	(d)	18, 19, 21
21	7.61	t	7.3 (20, 22)	20, 22	20, 22	133.8	(d)	19
22	7.49	t	7.3 (21, 23)	21, 23	21, 23	128.6	(d)	18, 21, 23
23	8.27	d	7.3 (22)	22	9, 22, 8-CH_3	129.8	(d)	17, 21
8-OCH_3	3.25	s			7, 9, 19, 23	51.2	(q)	8
9-OH	2.62	d	1.8 (9)	9	9			5, 8, 9

a, b As in Table S1

Table S5 NMR spectral data of **5** in $CDCl_3$

Position	δ_H[a]		J/Hz	^1H-^1H COSY	NOE	δ_C		HMBC (C)[b]
1								
2						87.0	(s)	
3	2.88	q	6.9 (16)	16	9, 14, 15, 16	44.0	(d)	2, 4, 10, 13, 16
4						202.7	(s)	
5						85.1	(s)	
6						168.7	(s)	
7	7.32	br s			8-OCH_3			
8						92.8	(s)	
9	4.44	s			3, 14, 19, 23, 8-OH_3, 9-OH	78.2	(d)	4, 5, 6, 8
10						203.4	(s)	
11	6.29	dd	6.2 (12), 2.0 (13)	12, 13	12, 16	131.6	(d)	2, 10, 12, 13
12	7.74	dd	6.2 (11), 2.0 (13)	11, 13	11, 13, 14, 15	164.4	(d)	2, 10, 11, 13
13	2.95	ddt	10.8 (14A), 6.2 (14B), 2.0 (11, 12)	11, 12, 14	12, 14, 15	51.7	(d)	
14A	1.54	ddq	14.0 (14B), 10.8 (13), 7.3 (15)	13, 15	3, 9, 12, 13, 15, 16	22.3	(t)	2, 12, 13, 15
14B	1.93	dqd	14.0 (14A), 7.3 (15), 6.2 (13)					
15	1.14	t	7.3 (14)	14	3, 12, 13, 14, 9-OH	12.3	(q)	13, 14
16	1.10	d	6.9 (3)	3	3, 11, 14	8.7	(q)	2, 3, 4
17						192.2	(s)	
18						133.9	(s)	
19	8.20	d	7.3 (20)	20	9, 20, 8-OCH_3	129.5	(d)	17, 21
20	7.49	t	7.3 (19, 21)	19, 21	19, 21	128.7	(d)	18, 19, 21
21	7.62	t	7.3 (20, 22)	20, 22	20, 22	134.0	(d)	19
22	7.49	t	7.3 (21, 23)	21, 23	21, 23	128.7	(d)	18, 21, 23
23	8.20	d	7.3 (22)	22	9, 22, 8-OCH_3	129.5	(d)	17, 21
8-OCH_3	3.25	s			7, 9, 19, 23	50.9	(q)	8
9-OH	2.96	br s			9, 15			

a, *b* As in Table S1

Table S6 NMR spectral data of **6** in $CDCl_3$

Position	δ_H[a]		J/Hz	^1H-^1H COSY	NOE	δ_C		HMBC (C)[b]
1								
2						86.3	(s)	
3	2.90	q	6.9 (16)	16	14, 15, 16, 9-OH	45.6	(d)	4, 5, 13, 16
4						205.0	(s)	
5						88.9	(s)	
6						167.7	(s)	
7	7.28	br s			9, 8-OCH_3			5
8						88.0	(s)	
9	4.19	d	11.0 (9-OH)	9-OH	7, 15, 19, 23, 9-OH	76.5	(d)	4, 5, 17
10						204.7	(s)	
11	6.27	dd	6.4 (12), 2.0 (13)	12	12, 16	131.5	(d)	2, 10, 12, 13
12	7.74	dd	6.4 (11), 2.0 (13)	11, 13	11, 13, 14, 15	164.9	(d)	2, 10, 11, 13
13	2.97	ddt	12.2 (14A), 6.6 (14B), 2.0 (11, 12)	11, 12, 14	12, 14, 15	51.8	(d)	
14A	1.43	ddq	14.0 (14B), 12.2 (13), 7.3 (15)	13, 15	3, 12, 13, 15, 16	22.4	(t)	2, 12, 13, 15
14B	1.89	dqd	14.0 (14A), 7.3 (15), 6.6 (13)					
15	1.15	t	7.3 (14)	14	3, 9, 12, 13, 14	12.2	(q)	13, 14
16	1.06	d	6.9 (3)	3	3, 11, 14	8.6	(q)	2, 3, 4
17						194.1	(s)	
18						132.9	(s)	
19	8.18	d	7.3 (20)	20	9, 20, 8-OCH_3, 9-OH	130.2	(d)	17, 20, 21
20	7.48	t	7.3 (19, 21)	19, 21	19, 21	128.7	(d)	18, 19, 21, 22
21	7.63	t	7.3 (20, 22)	20, 22	20, 22	134.3	(d)	19, 23
22	7.48	t	7.3 (21, 23)	21, 23	21, 23	128.7	(d)	18, 20, 21, 23
23	8.18	d	7.3 (22)	22	9, 22, 8-OCH_3, 9-OH	130.2	(d)	17, 21, 22
8-OCH_3	3.38	s			7, 19, 23	51.2	(q)	8
9-OH	3.55	d	11.0 (9)	9	3, 9, 19, 23			5, 9, 8-OCH_3

a, b As in Table 1

Table S7 NMR spectral data of **7** in $CDCl_3$

Position	δ_H[a]		J/Hz	^1H-^1H COSY	NOE	δ_C		HMBC (C)[b]
1								
2						88.5	(s)	
3	3.09	q	6.9 (16)	16	14, 15, 16	47.2	(d)	2, 4, 10, 13, 16
4						209.4	(s)	
5						83.0	(s)	
6						170.5	(s)	
7	7.14	br s			19, 23, 8-OCH_3			
8						96.7	(s)	
9	4.99	d	2.6 (9-OH)	9-OH	19, 23, 9-OH	81.8	(d)	4, 17
10						209.0	(s)	
11	6.25	dd	6.2 (12), 2.0 (13)	12	12, 16	131.1	(d)	2, 10, 12, 13
12	7.84	dd	6.2 (11), 2.0 (13)	11, 13	11, 13, 14, 15	167.4	(d)	2, 10, 11, 13
13	3.14	ddt	10.8 (14A), 6.2 (14B), 2.0 (11, 12)	11, 12, 14	12, 14, 15	50.1	(d)	
14A	1.54	ddq	14.0 (14B), 10.8 (13), 7.3 (15)	13, 15	3, 12, 13, 15, 16	21.2	(t)	2, 12, 13, 15
14B	1.93	dqd	14.0 (14A), 7.3 (15), 6.2 (13)					
15	1.22	t	7.3 (14)	14	3, 12, 13, 14	12.2	(q)	13, 14
16	0.97	d	6.9 (3)	3	3, 11, 14	8.6	(q)	2, 3, 4
17						192.7	(s)	
18						133.9	(s)	
19	8.25	d	7.3 (20)	20	7, 9, 20, 8-OCH_3, 9-OH	129.7	(d)	17, 21
20	7.48	t	7.3 (19, 21)	19, 21	19, 21	128.6	(d)	18, 19, 21
21	7.61	t	7.3 (20, 22)	20, 22	20, 22	133.8	(d)	19
22	7.48	t	7.3 (21, 23)	21, 23	21, 23	128.6	(d)	18, 21, 23
23	8.25	d	7.3 (22)	22	7, 9, 22, 8-OCH_3, 9-OH	129.7	(d)	17, 21
8-OCH_3	3.20	s			7, 19, 23	51.2	(q)	8
9-OH	4.44	d	2.6 (9)	9	9, 19, 23			9

a, *b* As in Table S1

Table S8 NMR spectral data of **8** in $CDCl_3$

Position	δ_H[a]		J/Hz	^1H-^1H COSY	NOE	δ_C		HMBC (C)[b]
1								
2						89.4	(s)	
3	3.19	q	7.1 (16)	16	14, 15, 16	46.2	(d)	2, 4, 10, 16
4						205.8	(s)	
5						85.0	(s)	
6						169.4	(s)	
7	6.98	br s			8, 9, 8-CH_3			5, 8, 9
8						93.5	(s)	
9	4.62	d	2.3 (9-OH)	9-OH	7, 16, 19, 23, 8-OCH_3, 9-OH	70.1	(d)	4, 17
10						211.9	(s)	
11	6.27	dd	6.2 (12), 2.0 (13)	12	12, 16	131.1	(d)	2, 10, 12, 13
12	7.84	dd	6.2 (11), 2.0 (13)	11, 13	11, 13, 14, 15	167.7	(d)	2, 10, 13
13	3.20	ddt	12.0 (14A), 6.8 (14B), 2.0 (11, 12)	11, 12, 14	12, 14, 15	50.5	(d)	2, 10, 12, 14
14A	1.56	ddq	14.0 (14B), 12.0 (13), 7.3 (15)	13, 15	3, 12, 13, 15, 16	21.0	(t)	2, 12, 13, 15
14B	1.96	dqd	14.0 (14A), 7.3 (15), 6.8 (13)					
15	1.25	t	7.3 (14)	14	3, 12, 13, 14	12.1	(q)	13, 14
16	0.96	d	7.1 (3)	3	3, 9, 11, 14	8.8	(q)	2, 3, 4
17						194.1	(s)	
18						132.7	(s)	
19	8.48	d	7.3 (20)	20	9, 20, 8-OCH_3, 9-OH	131.2	(d)	17, 21, 23
20	7.49	t	7.3 (19, 21)	19, 21	19, 21	128.4	(d)	18, 19, 21, 22
21	7.63	t	7.3 (20, 22)	20, 22	20, 22	134.4	(d)	19, 20, 22, 23
22	7.49	t	7.3 (21, 23)	21, 23	21, 23	128.4	(d)	18, 21, 23
23	8.48	d	7.3 (22)	22	9, 22, 8-OCH_3, 9-OH	131.2	(d)	17, 19, 21
8-OCH_3	3.33	s			7, 9, 19, 23, 9-OH	51.8	(q)	8
9-OH	4.52	d	2.3 (9)	9	9, 19, 23, 8-OCH_3			8, 9

a, b As in Table S1

Figure S1 ^{1}H and ^{13}C NMR spectra of **1** in $CDCl_3$

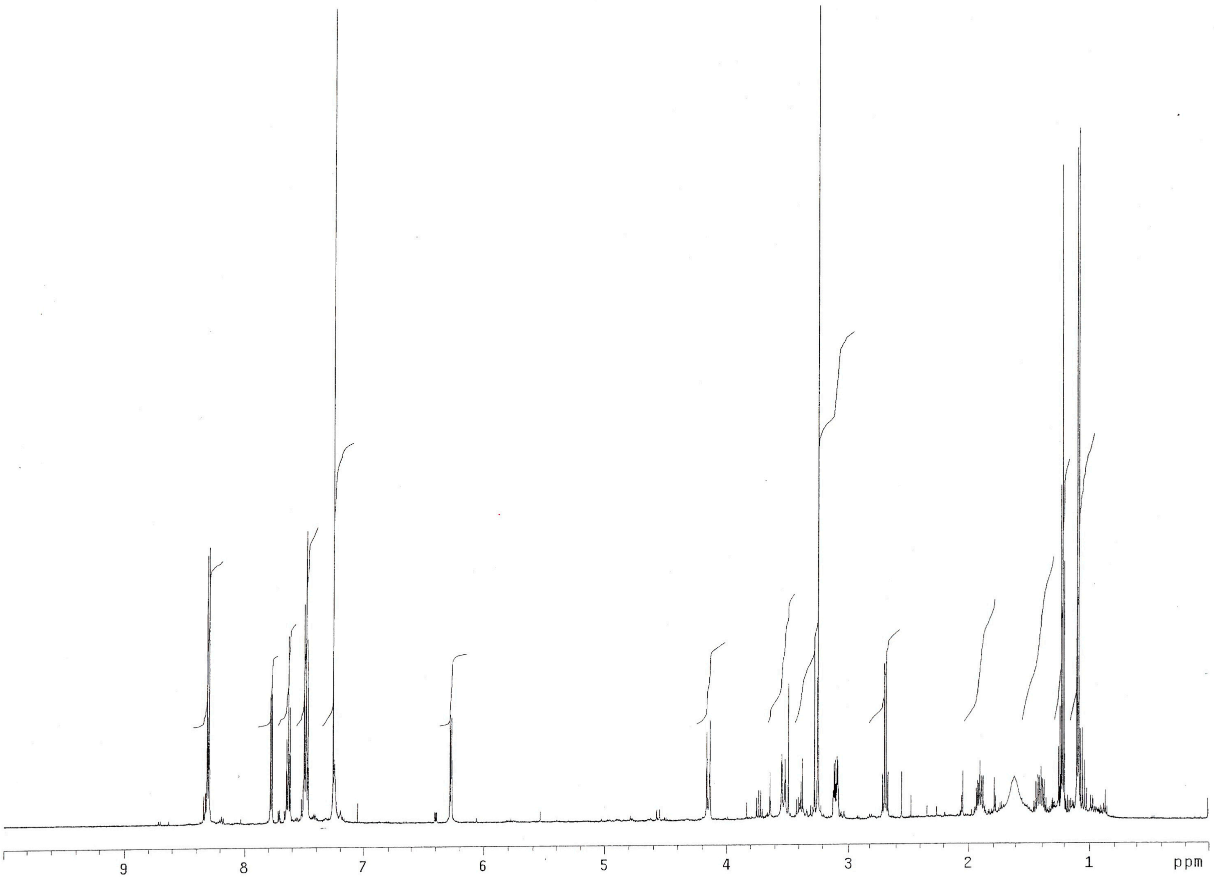

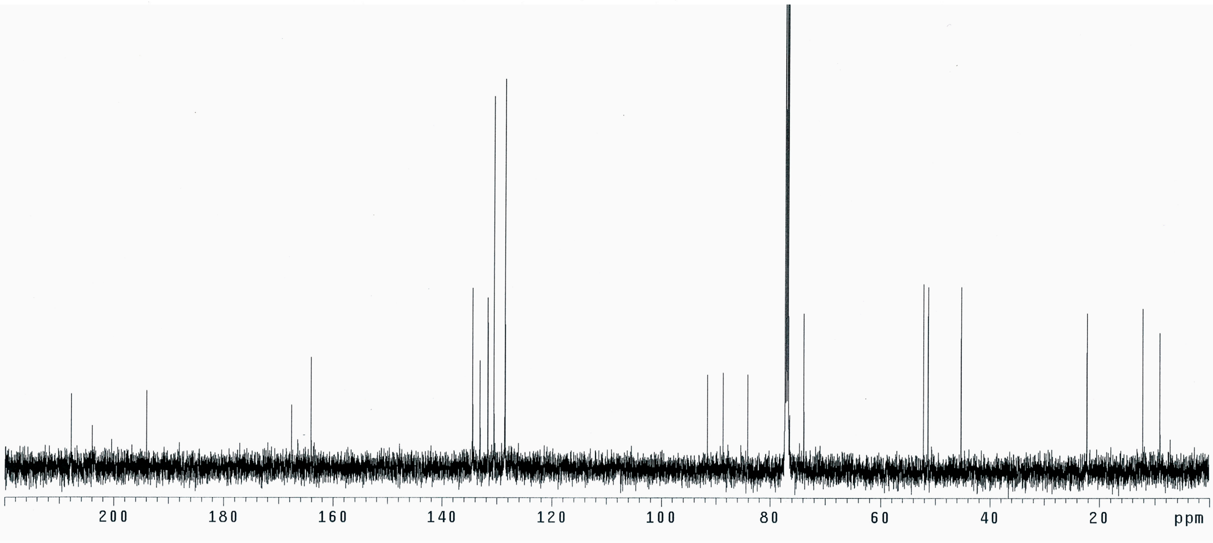

Figure S2 ^1H-^1H COSY of **1**

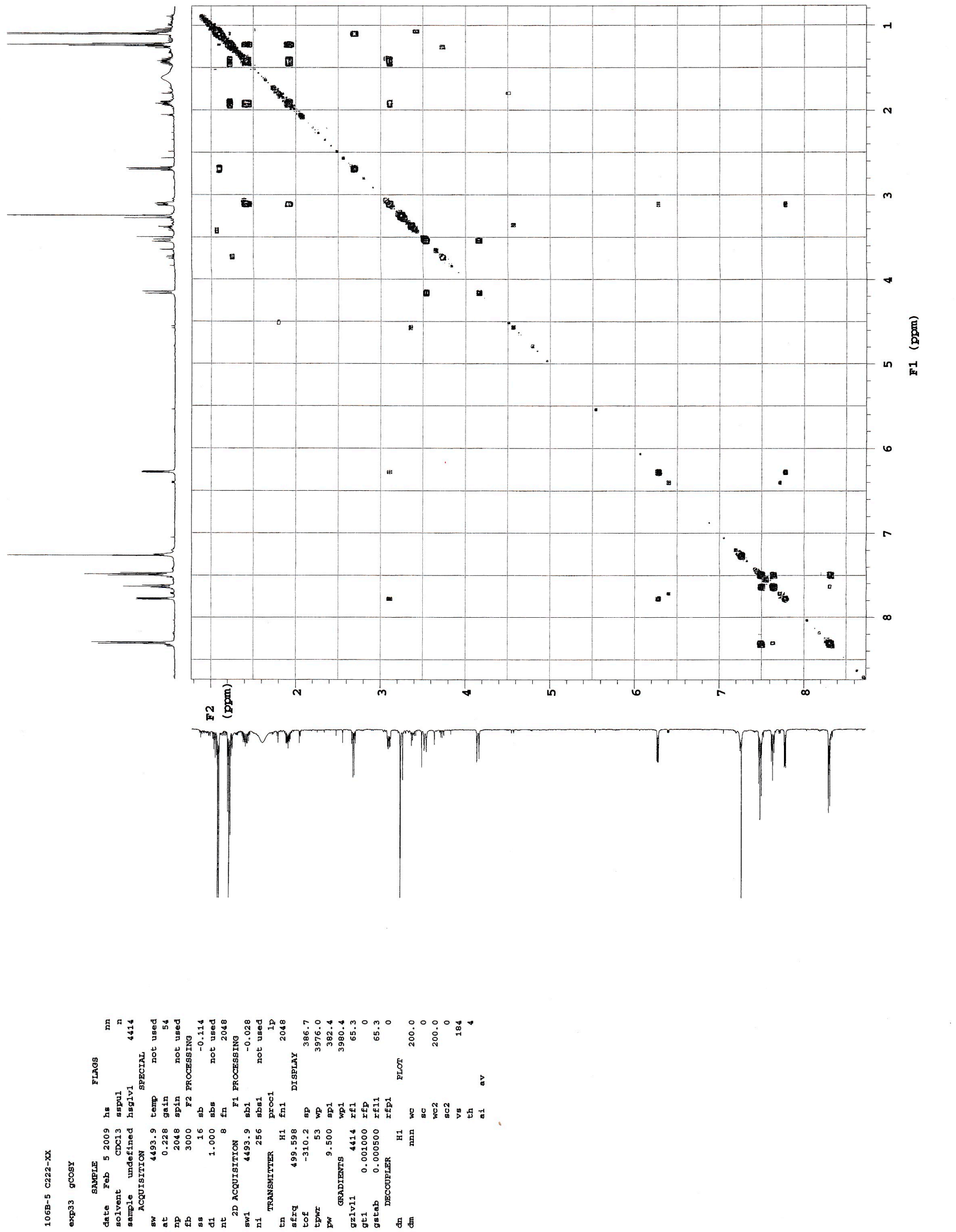

Figure S3 NOESY of **1**

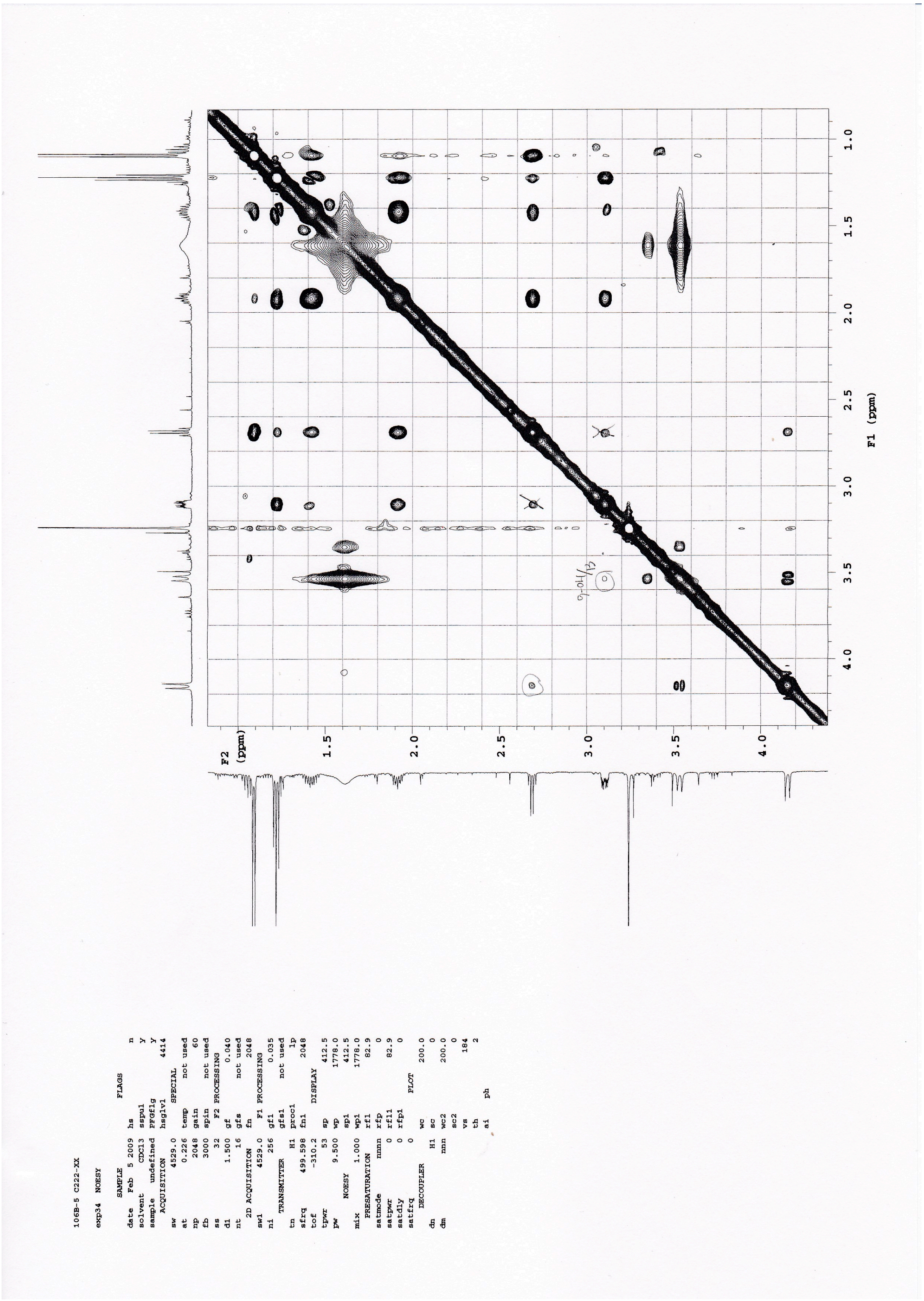

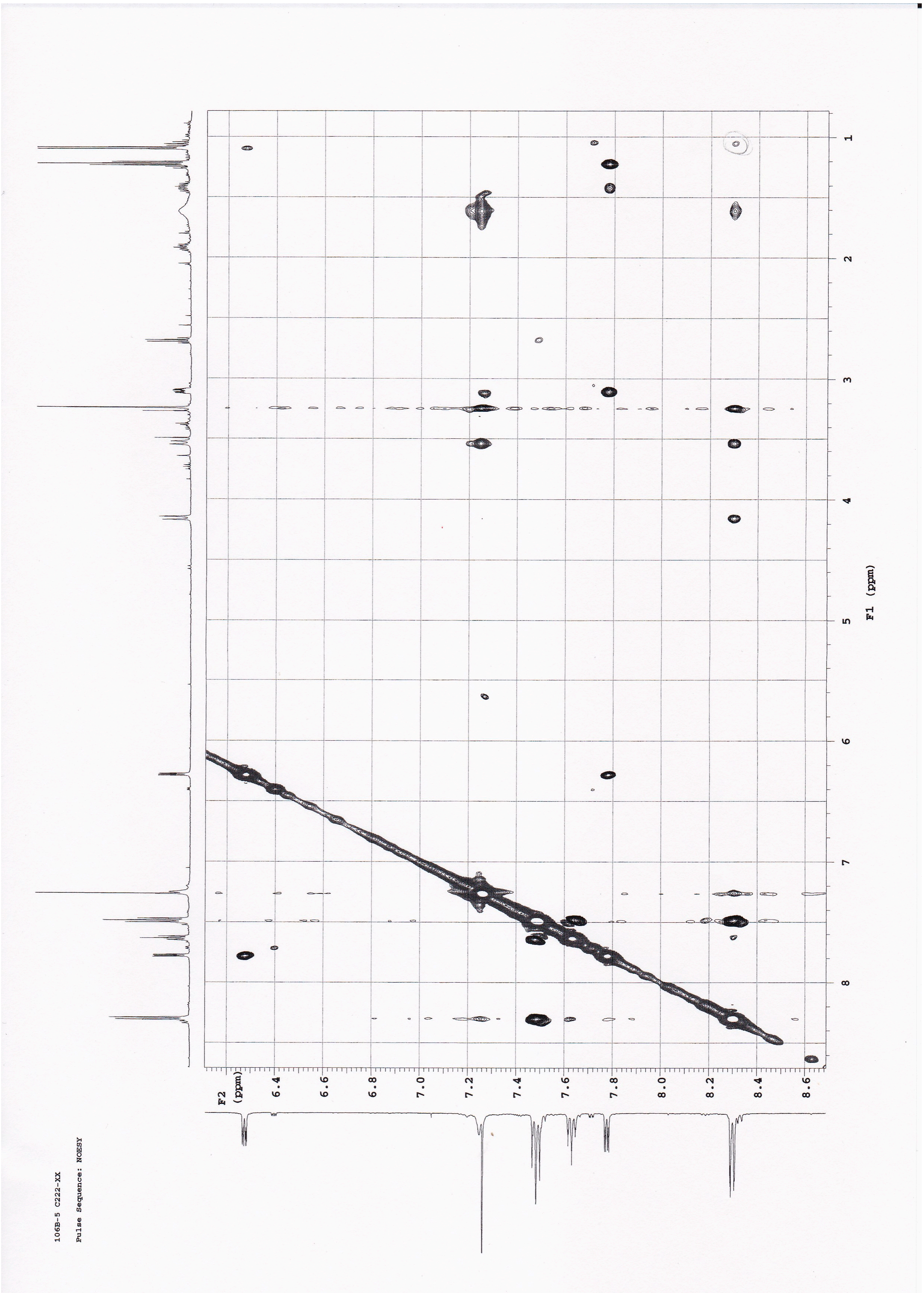
106B-5 C222-XX
Pulse Sequence: NOESY
F2 (ppm)
F1 (ppm)

Figure S4 HMQC of **1**

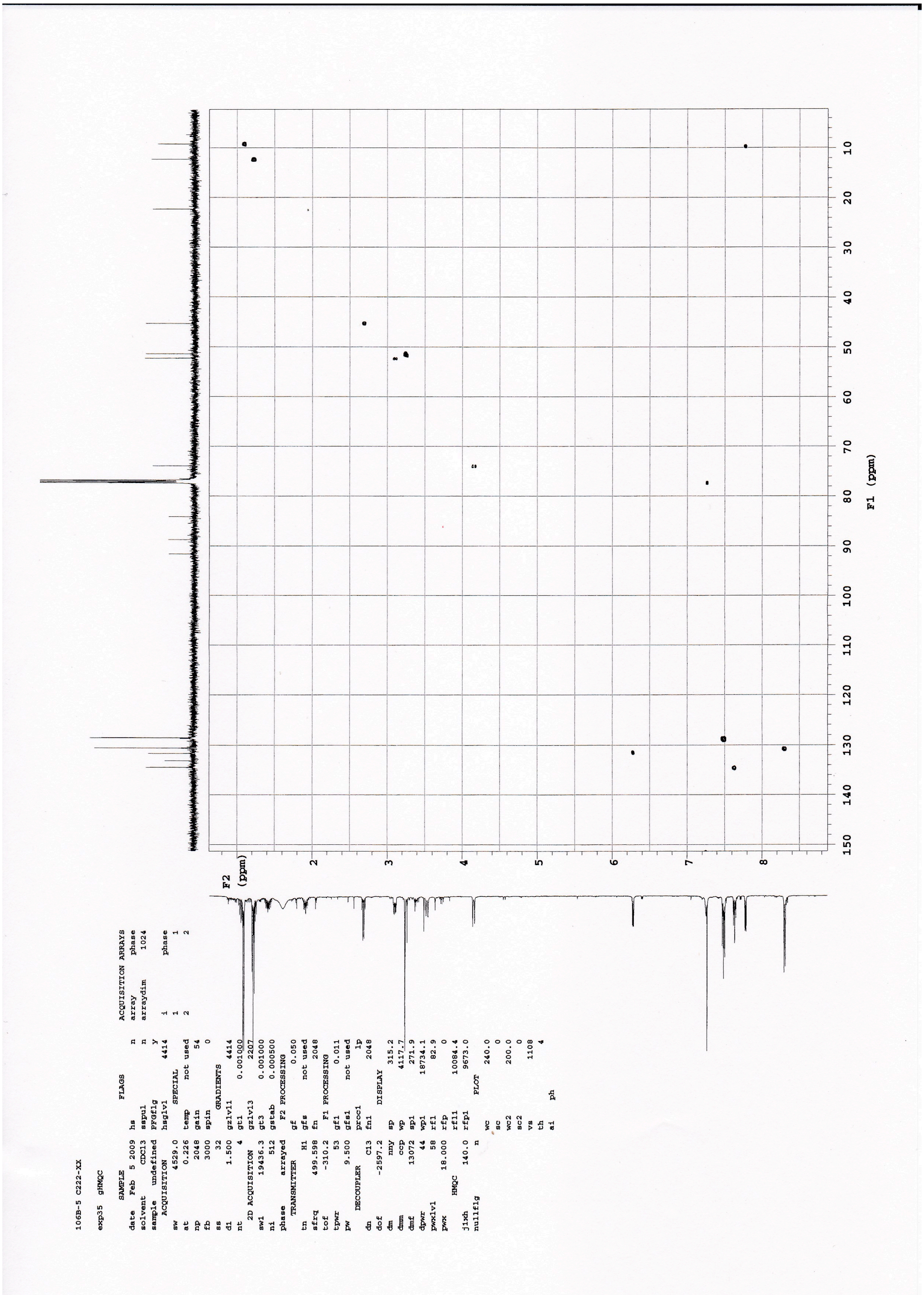

Figure S5 HMBC of **1**

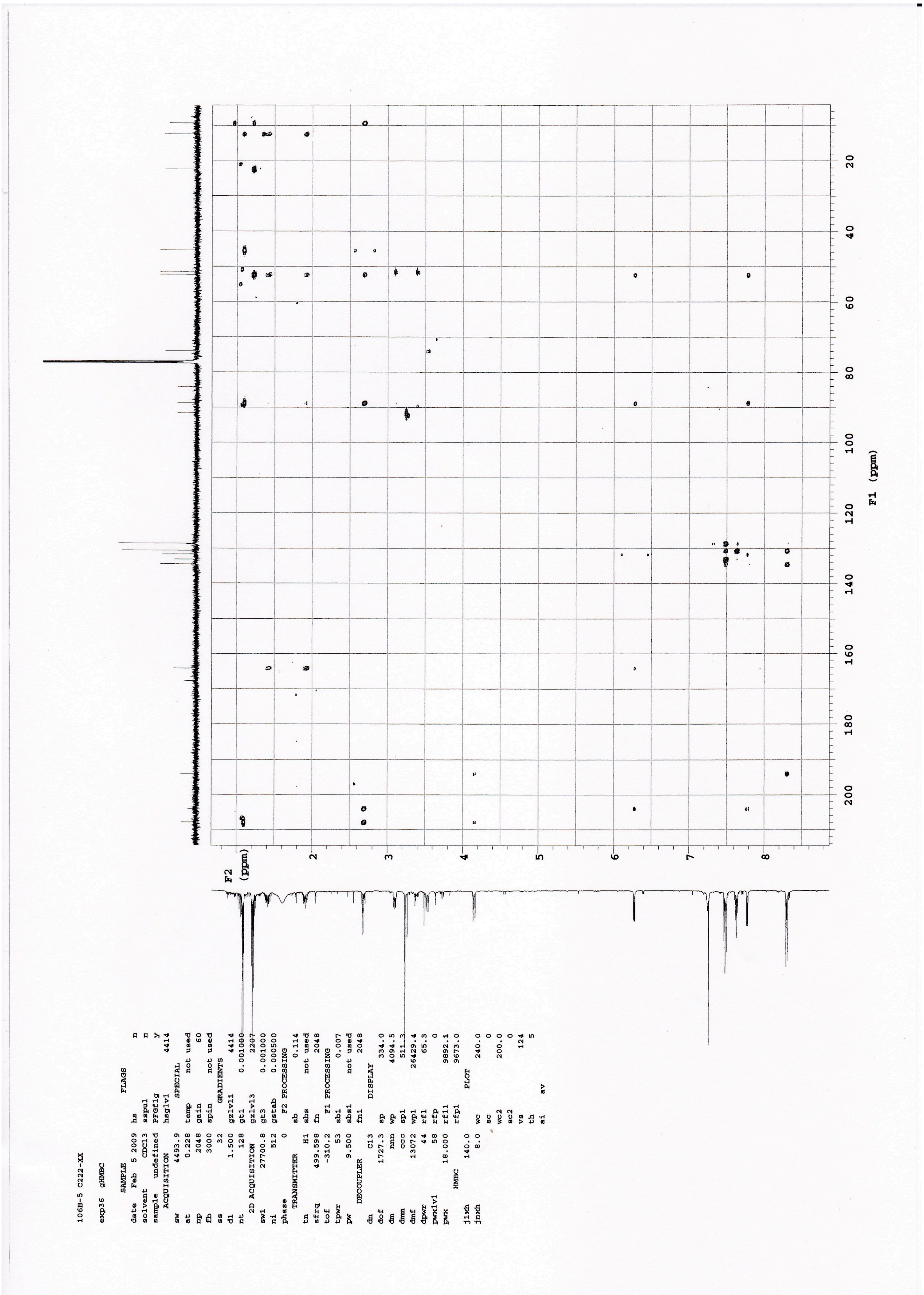

Figure S6 IR spectrum of **1**

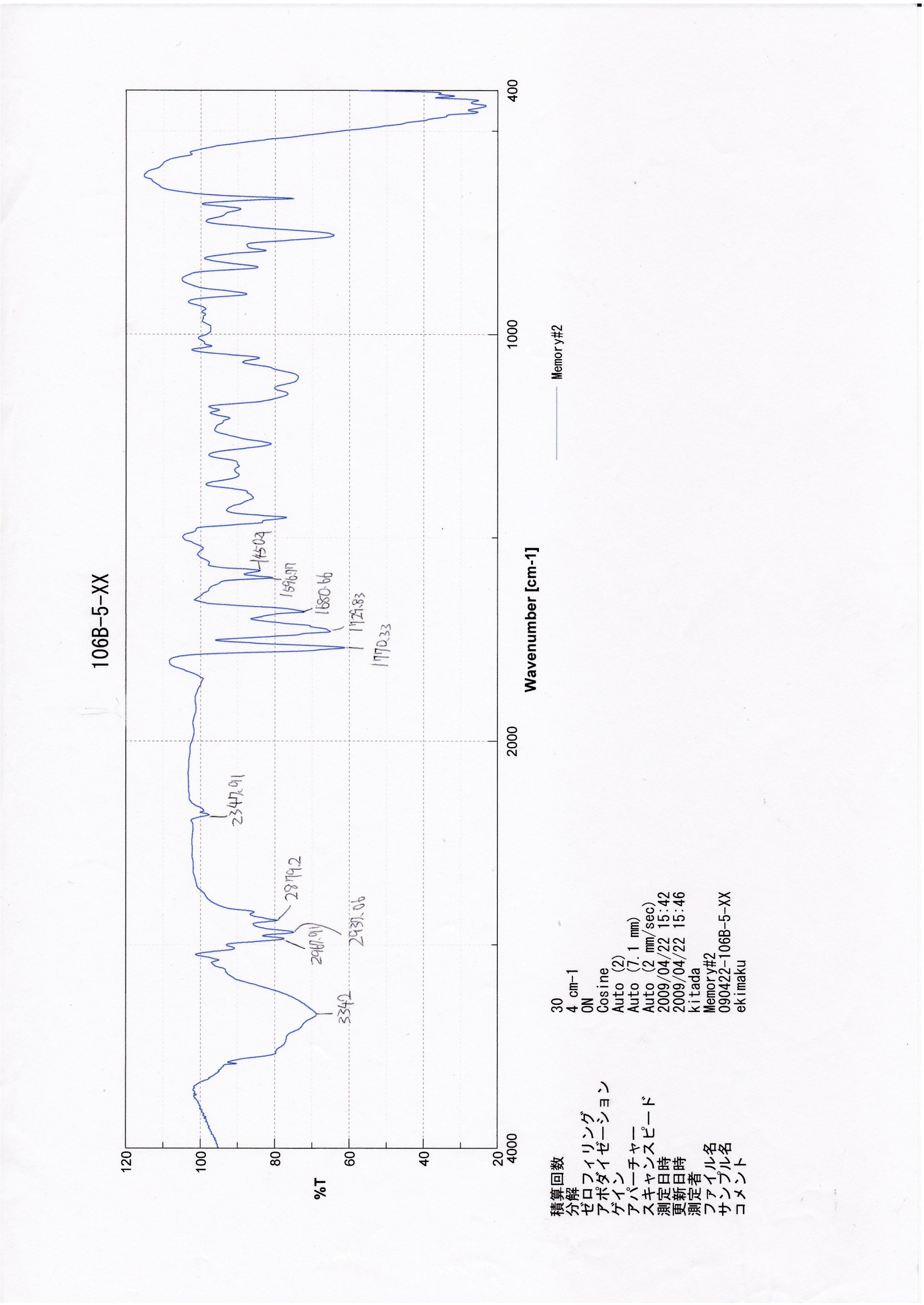

Figure S7 FABMS of **1**

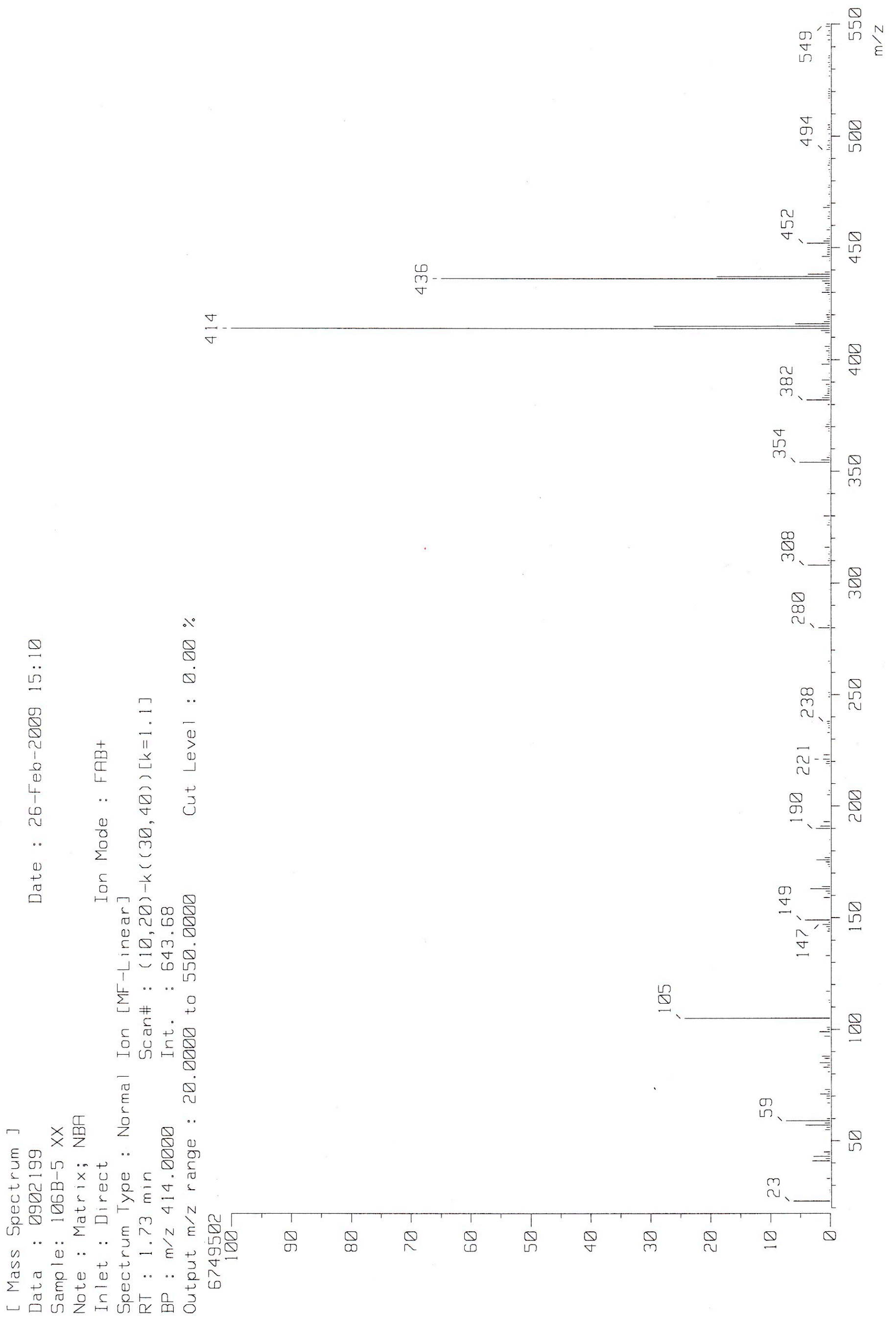

Figure S8 ^{1}H and ^{13}C NMR spectra of **2** in $CDCl_3$

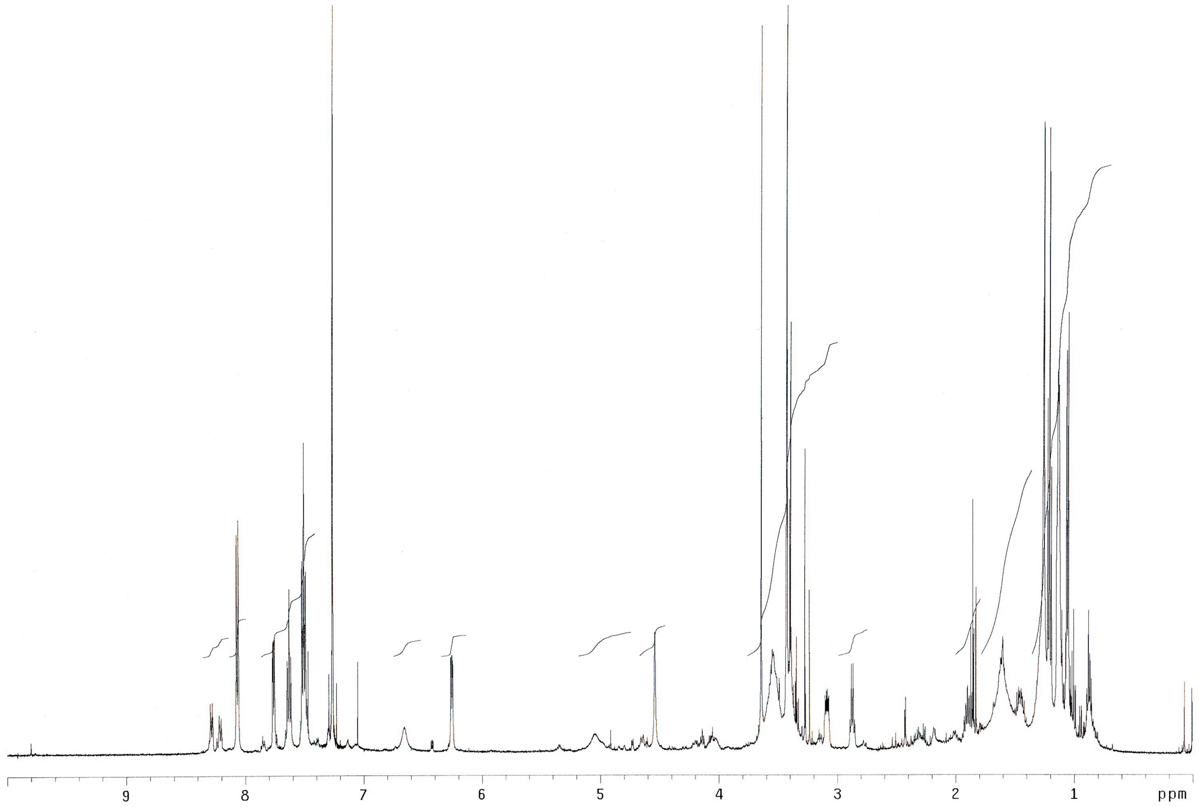

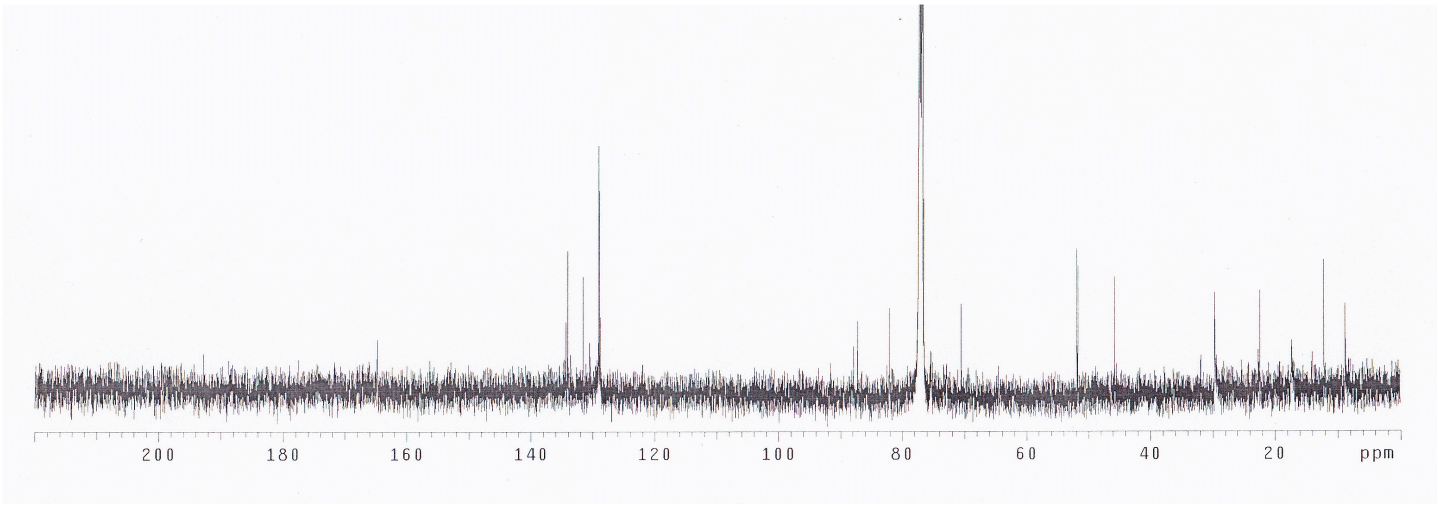

Figure S9 H-^{1}H COSY of **2**

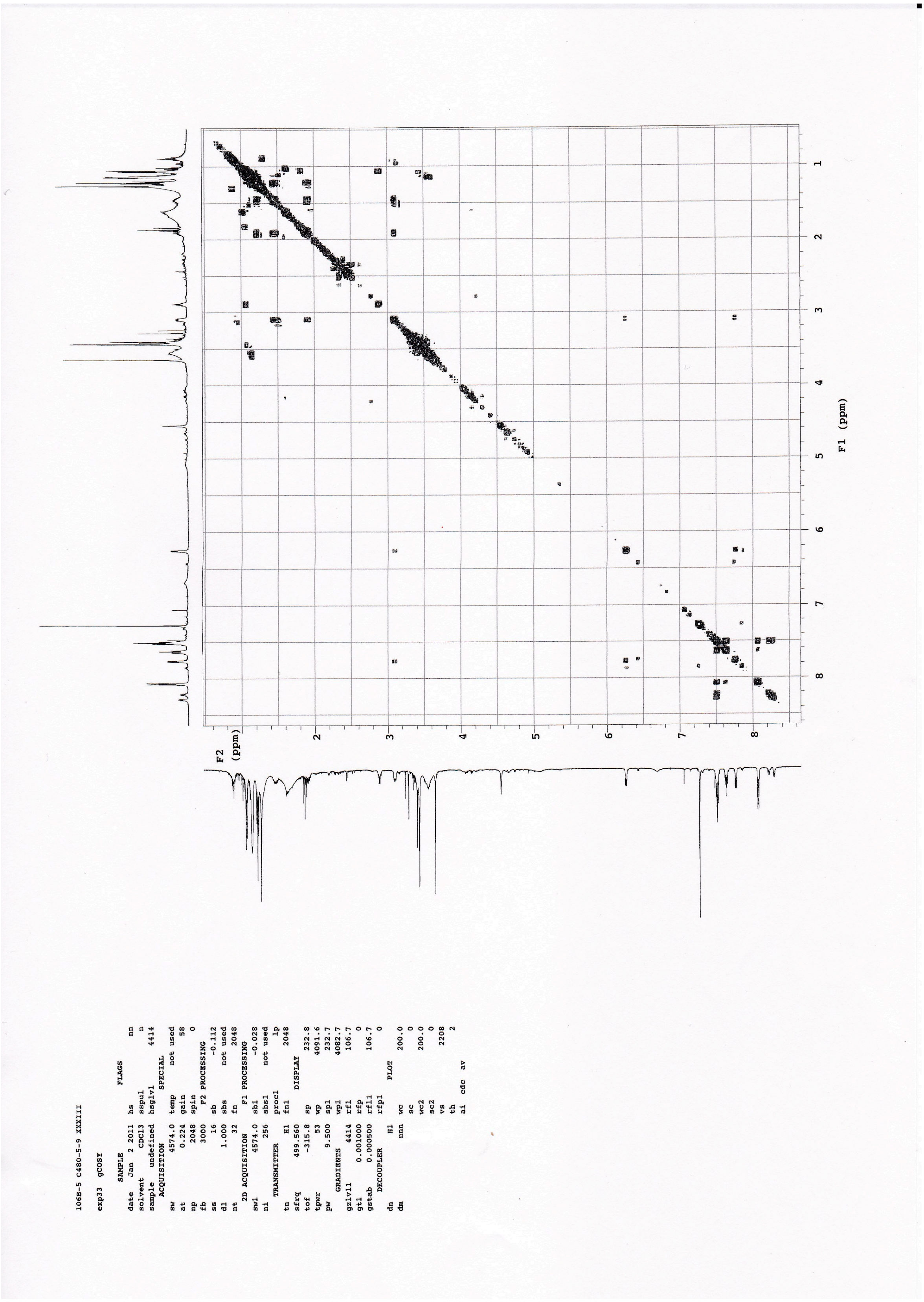

Figure S10 NOESY of **2**

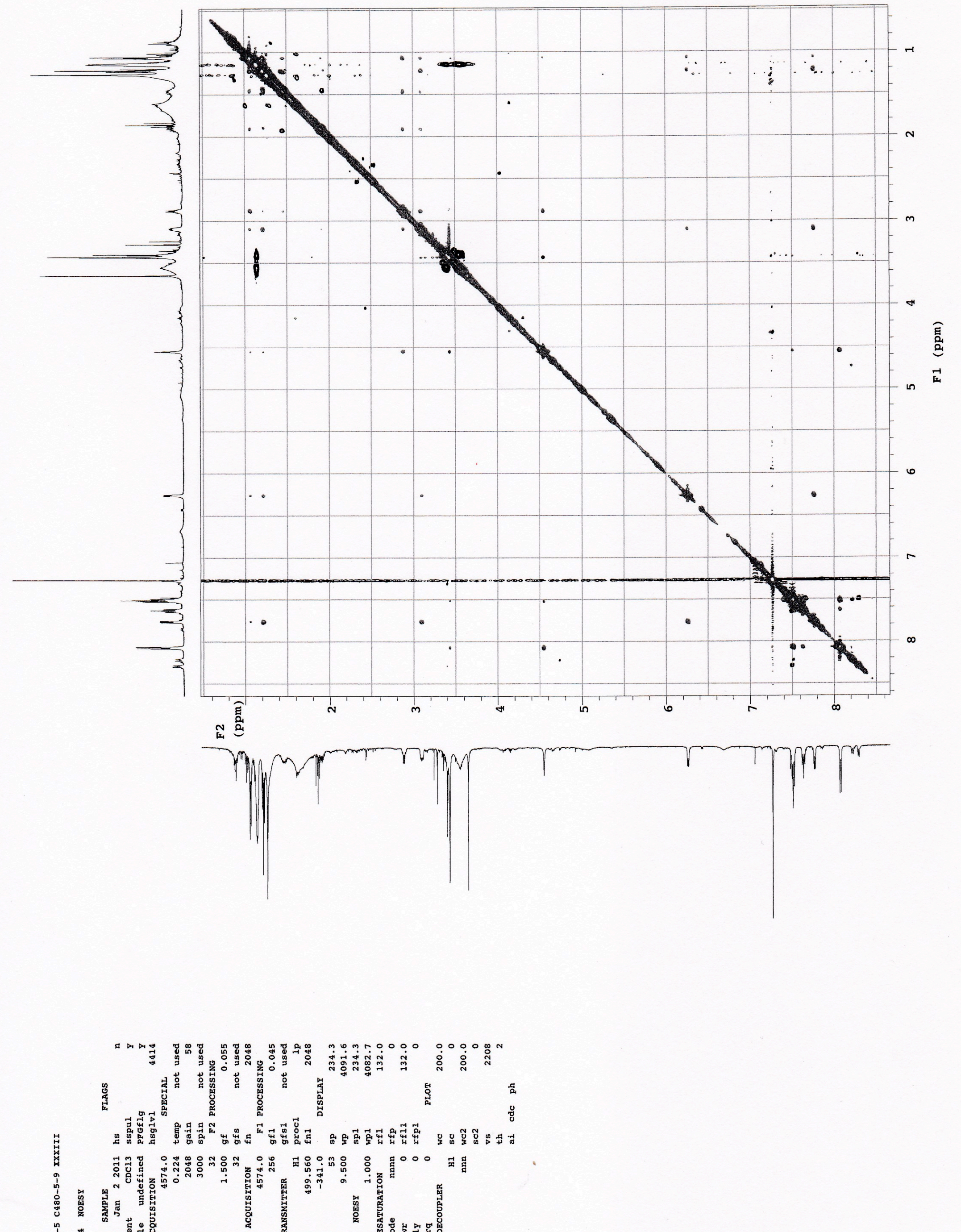

Figure S11 HMQC of **2**

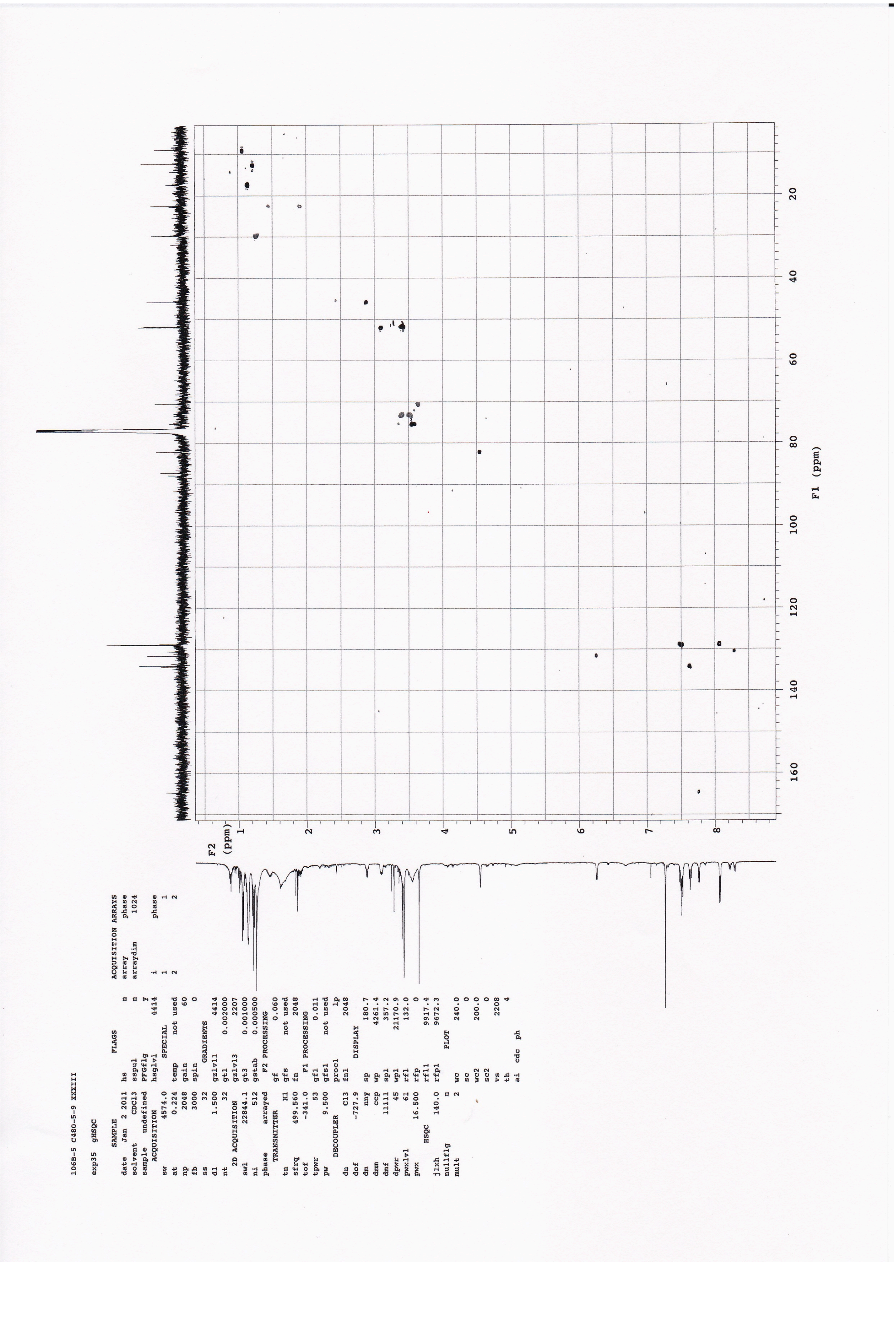

Figure S12 HMBC of **2**

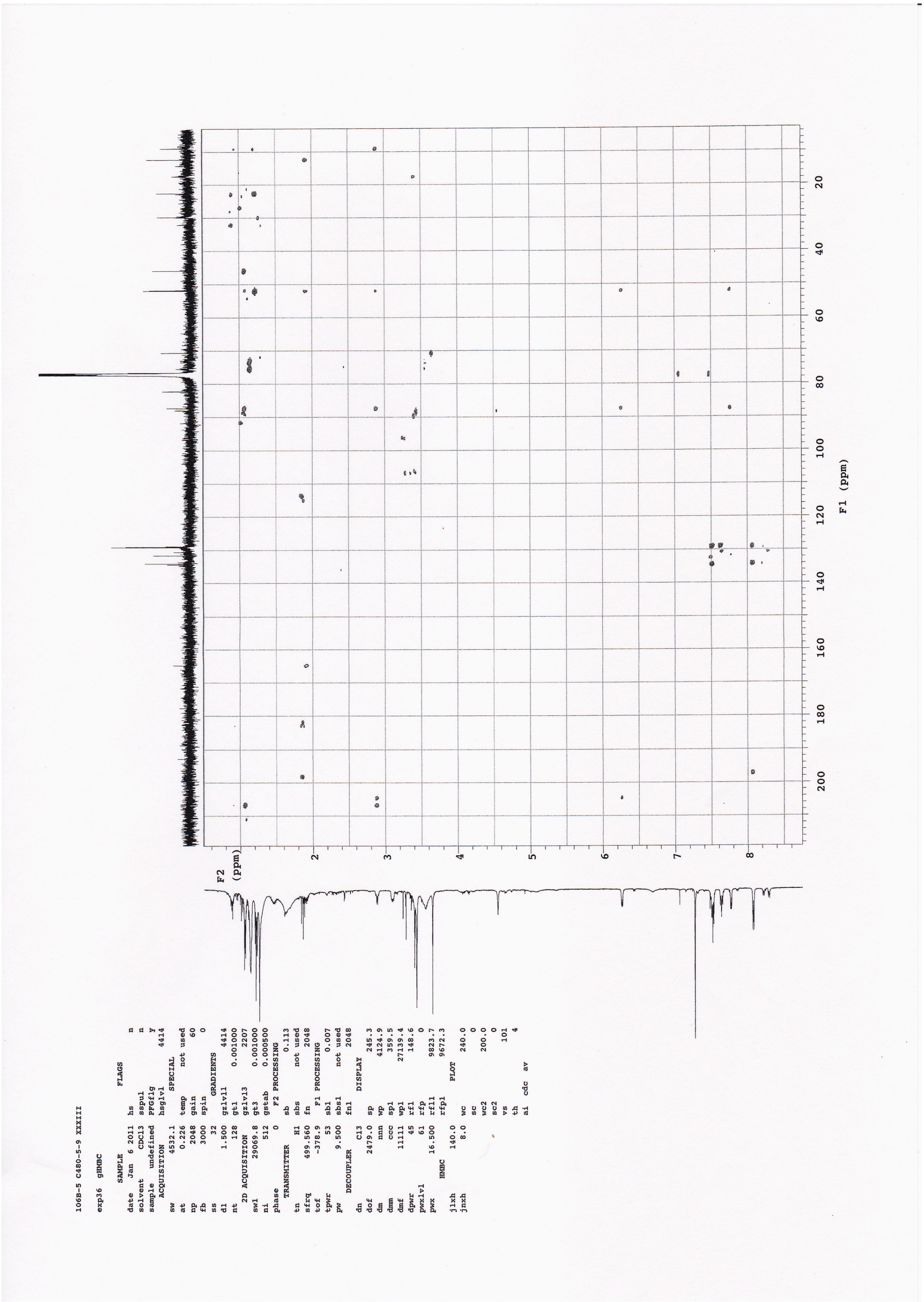

Figure S13 IR spectrum of **2**

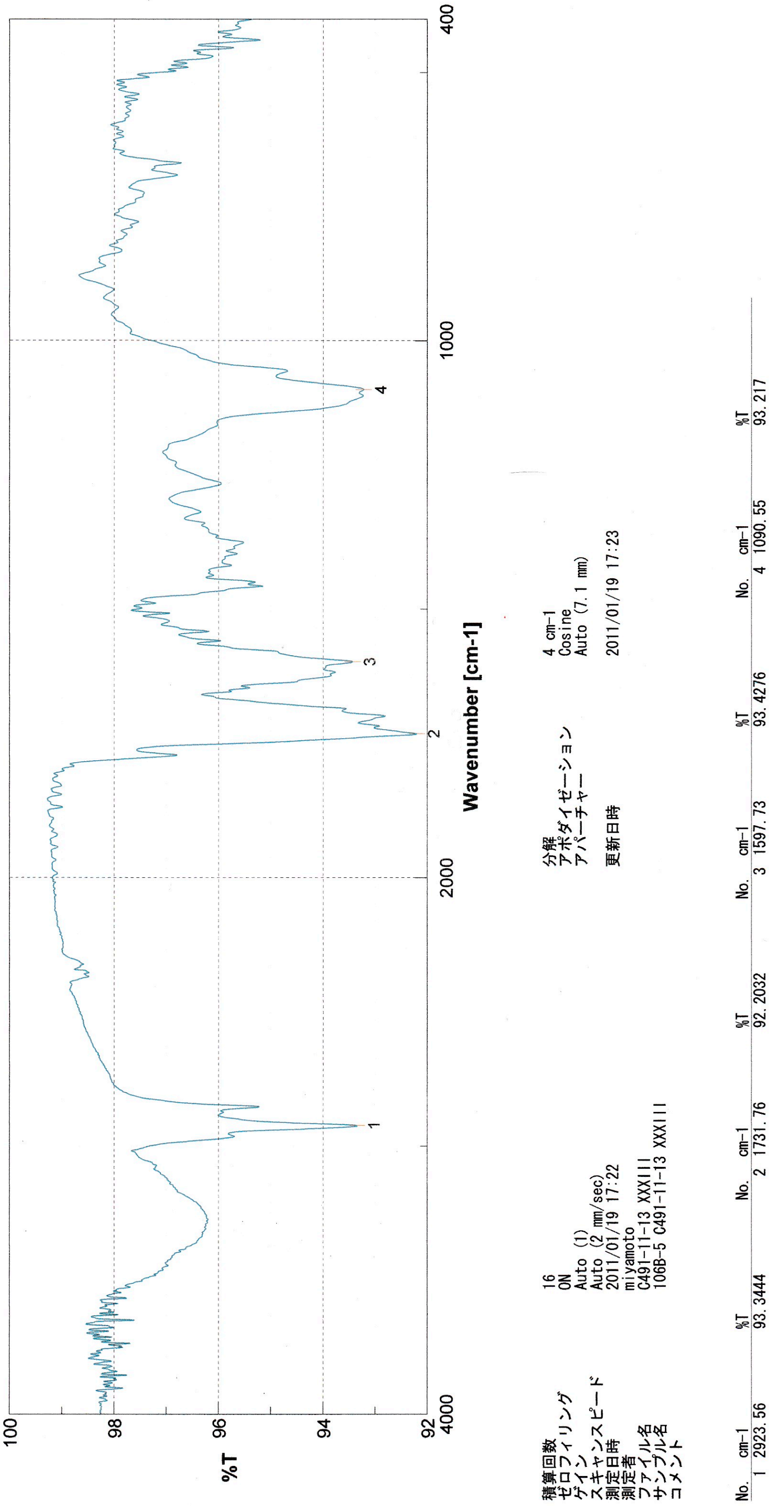

Figure S14 FABMS of **2**

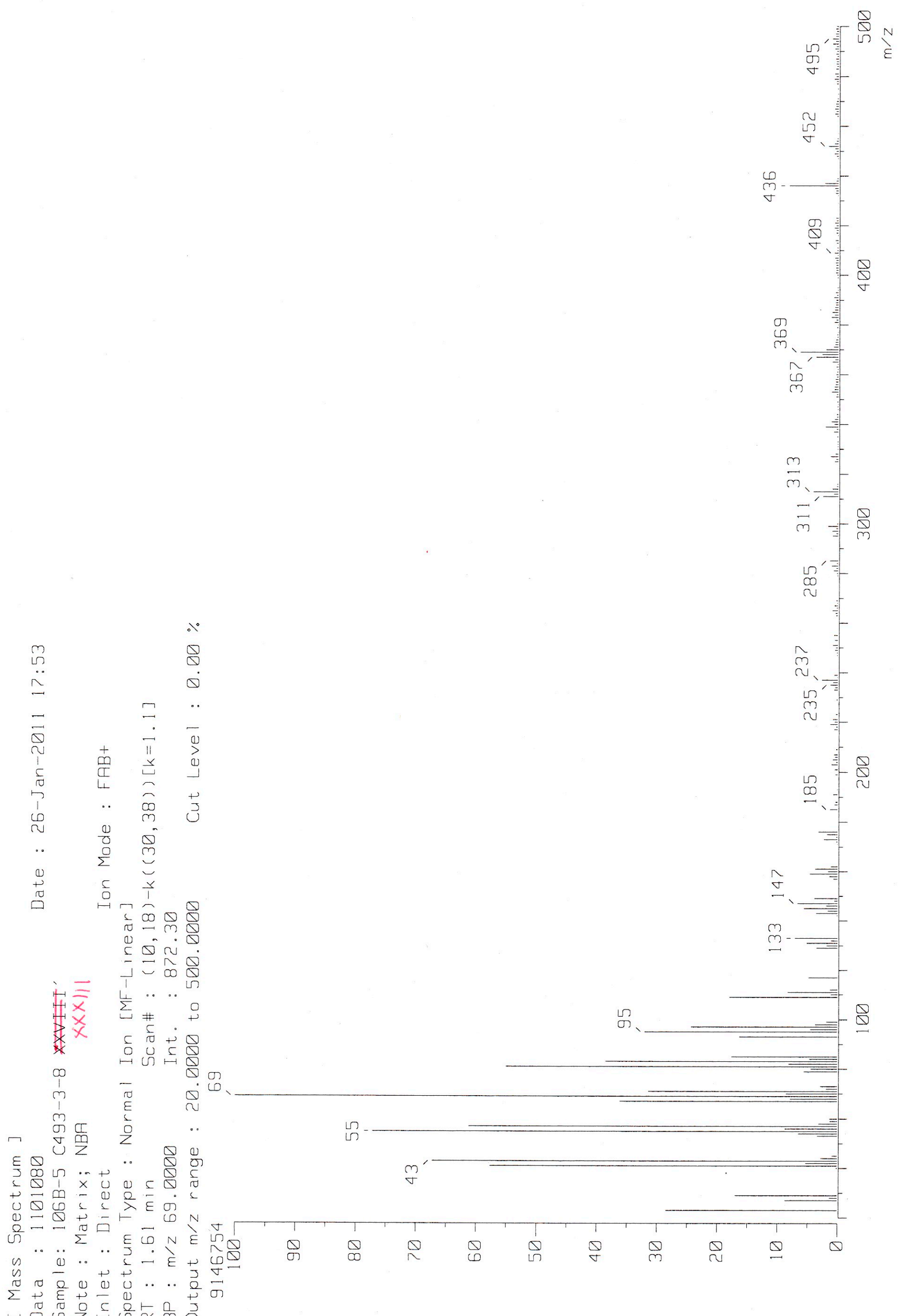

Figure S15 ^{1}H and ^{13}C NMR spectra of **3** in $CDCl_3$

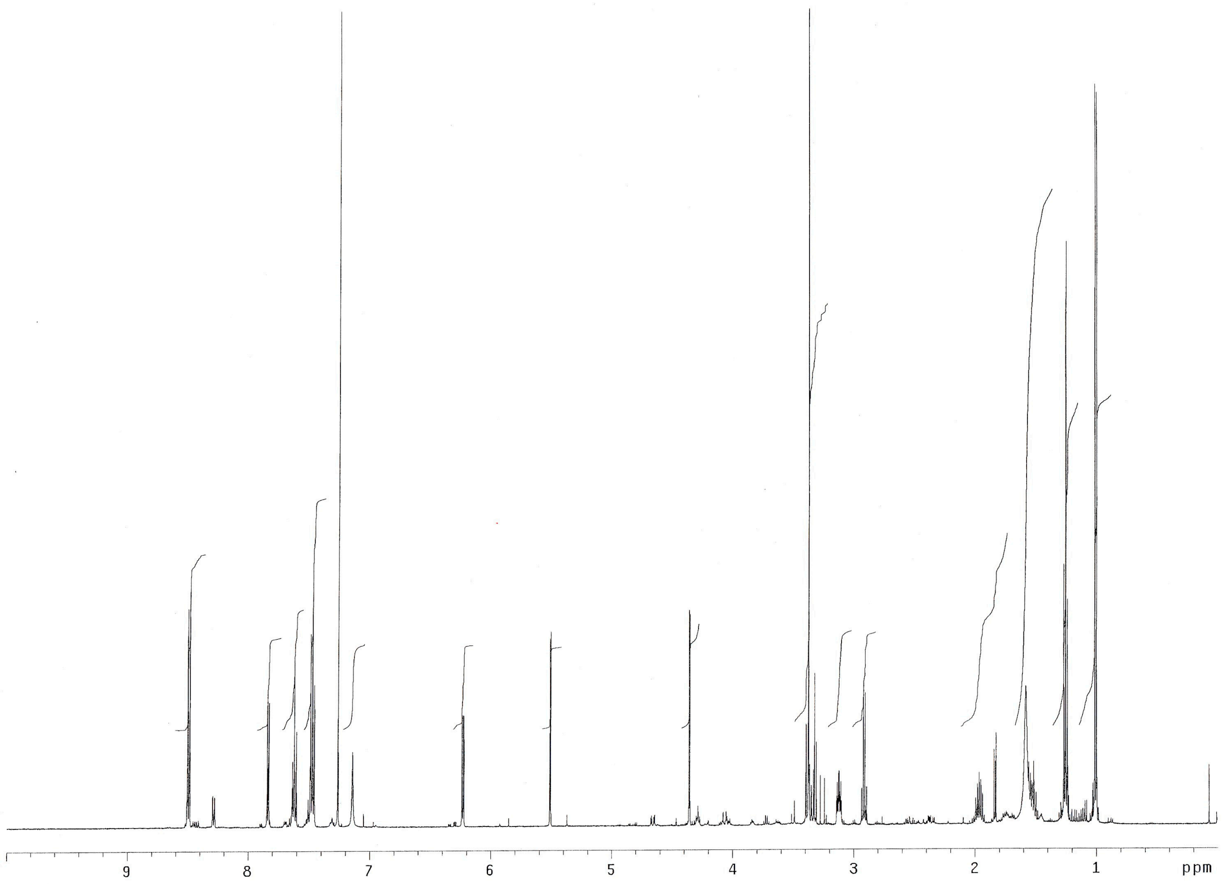

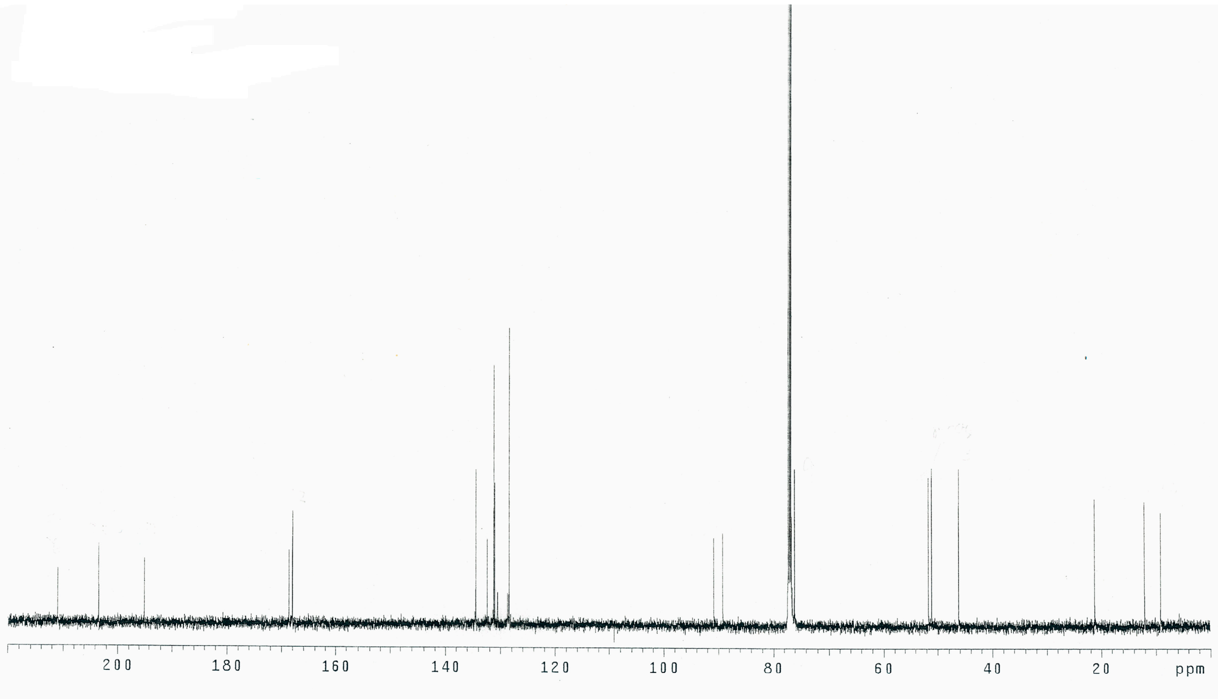

Figure S16 ^{1}H-^{1}H COSY of **3**

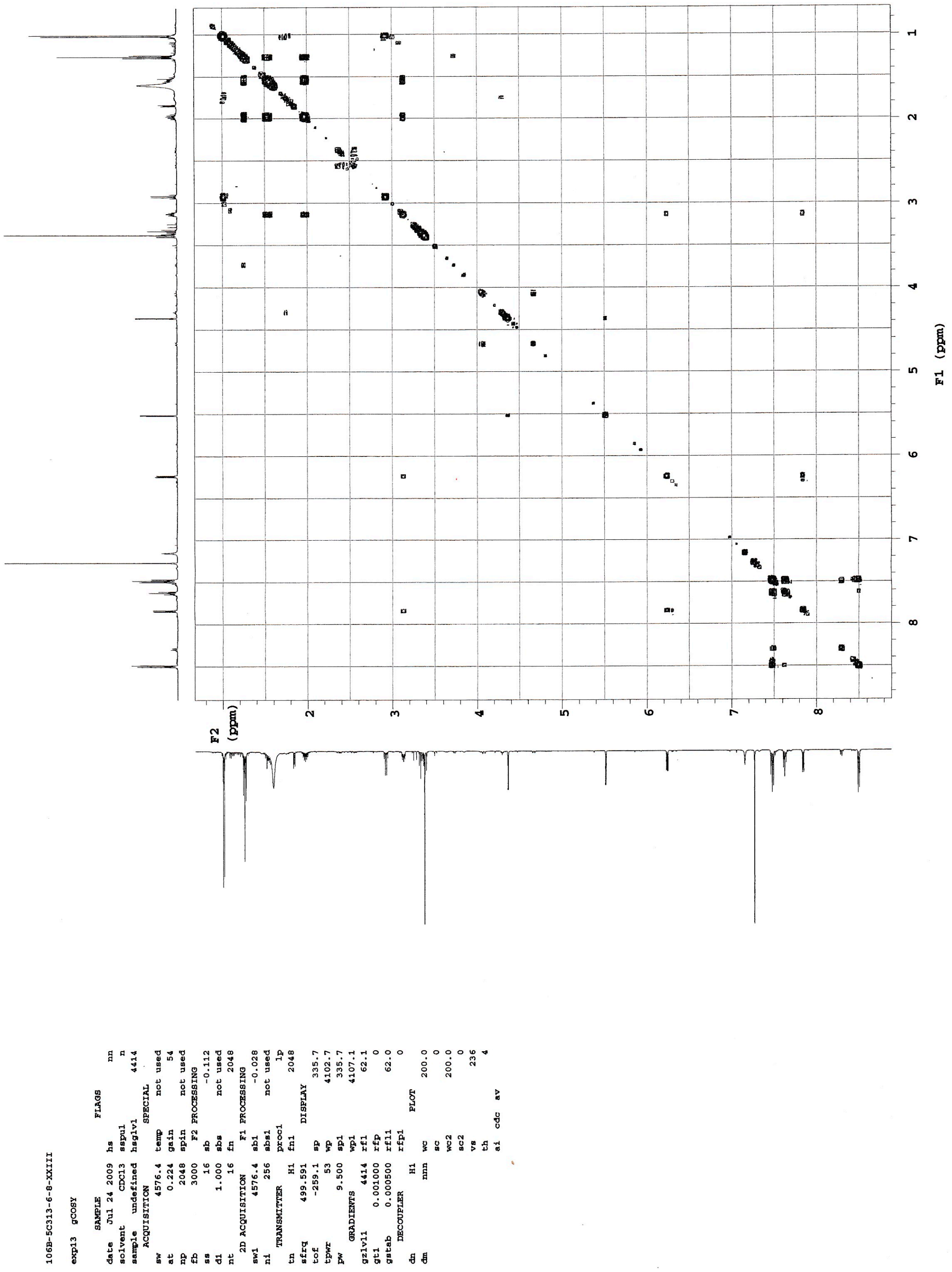

Figure S17 NOESY of **3**

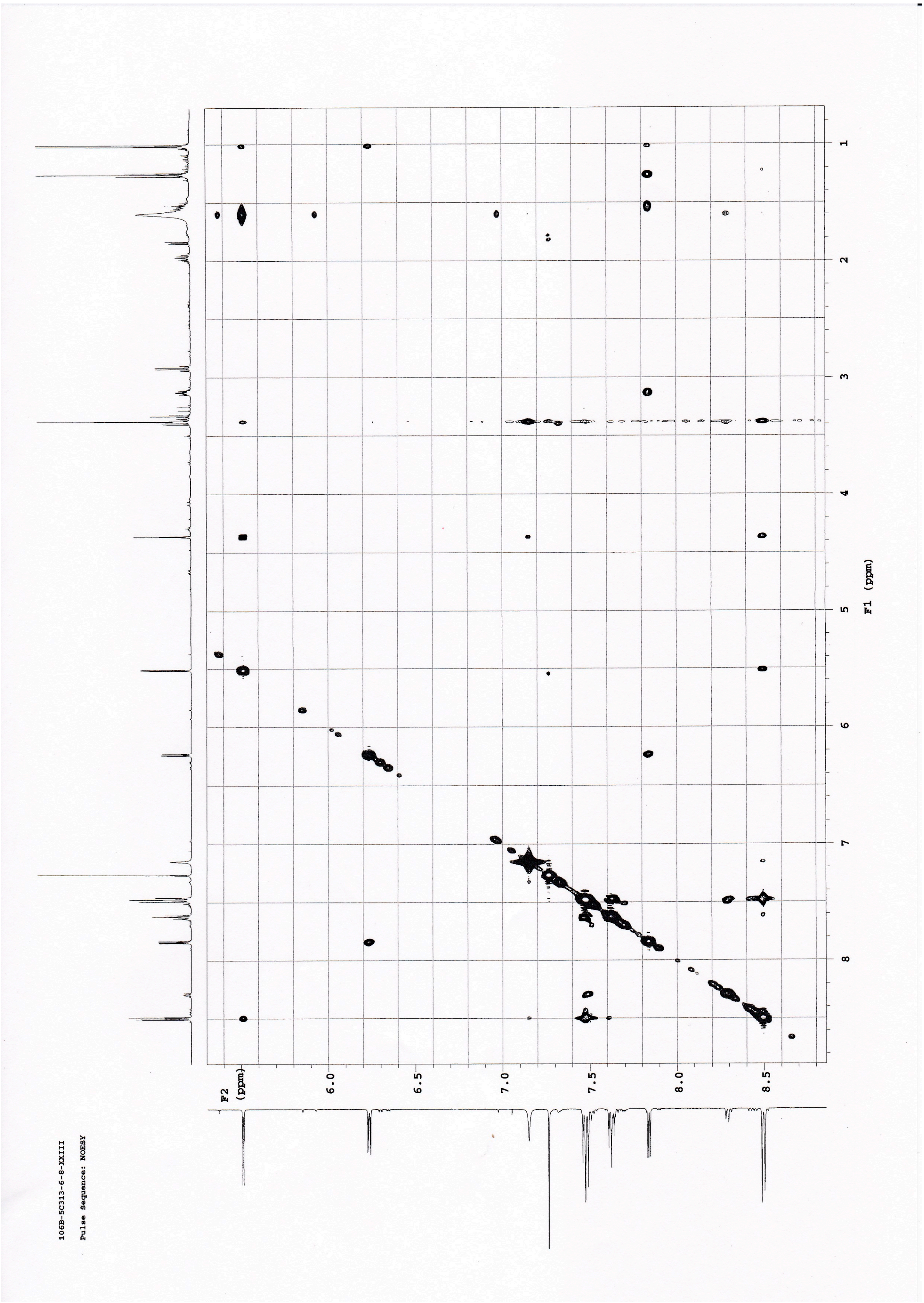

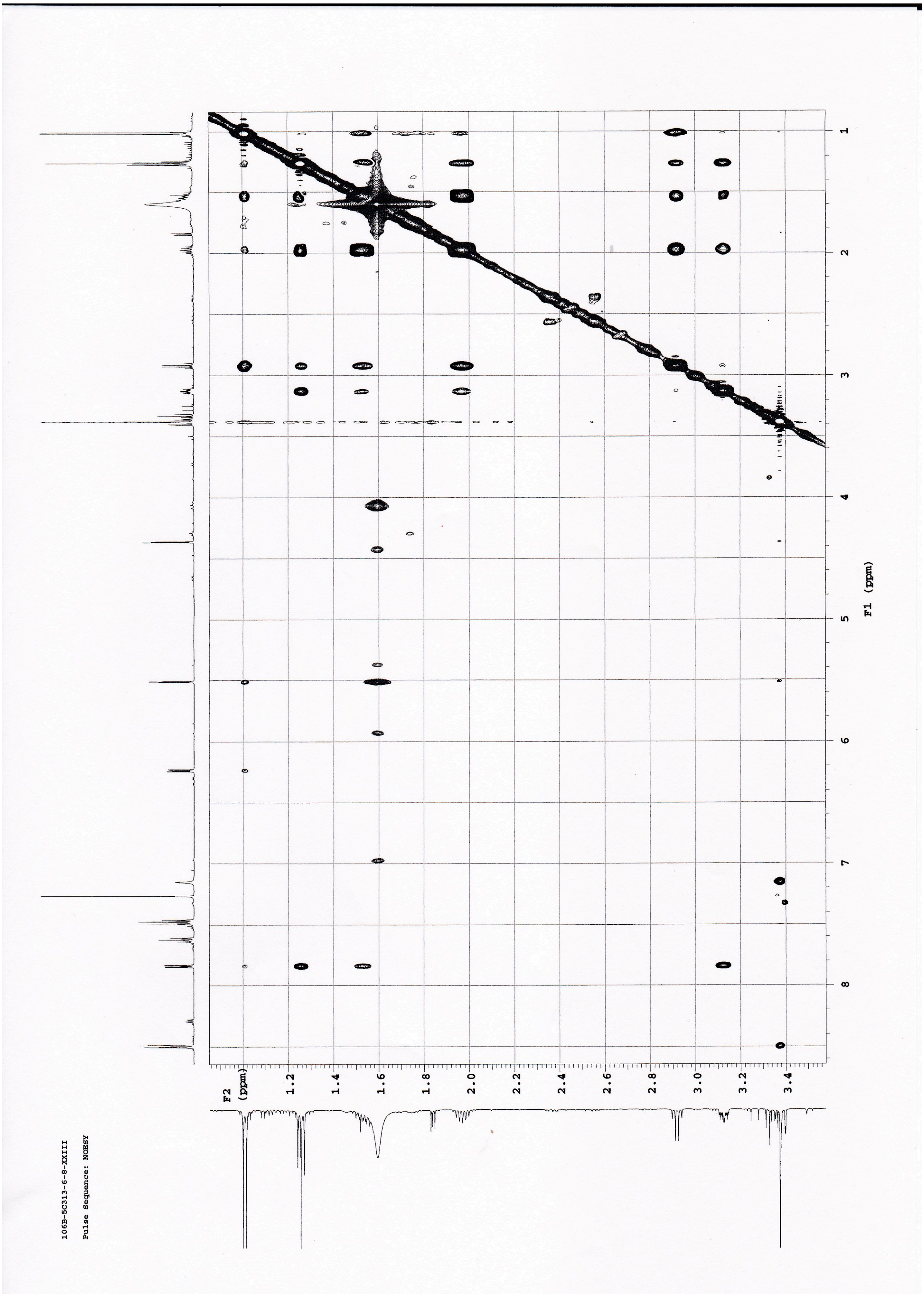
106B-5C313-6-8-XXIII
Pulse Sequence: NOESY
F2 (ppm)
F1 (ppm)

Figure S18 HMQC of **3**

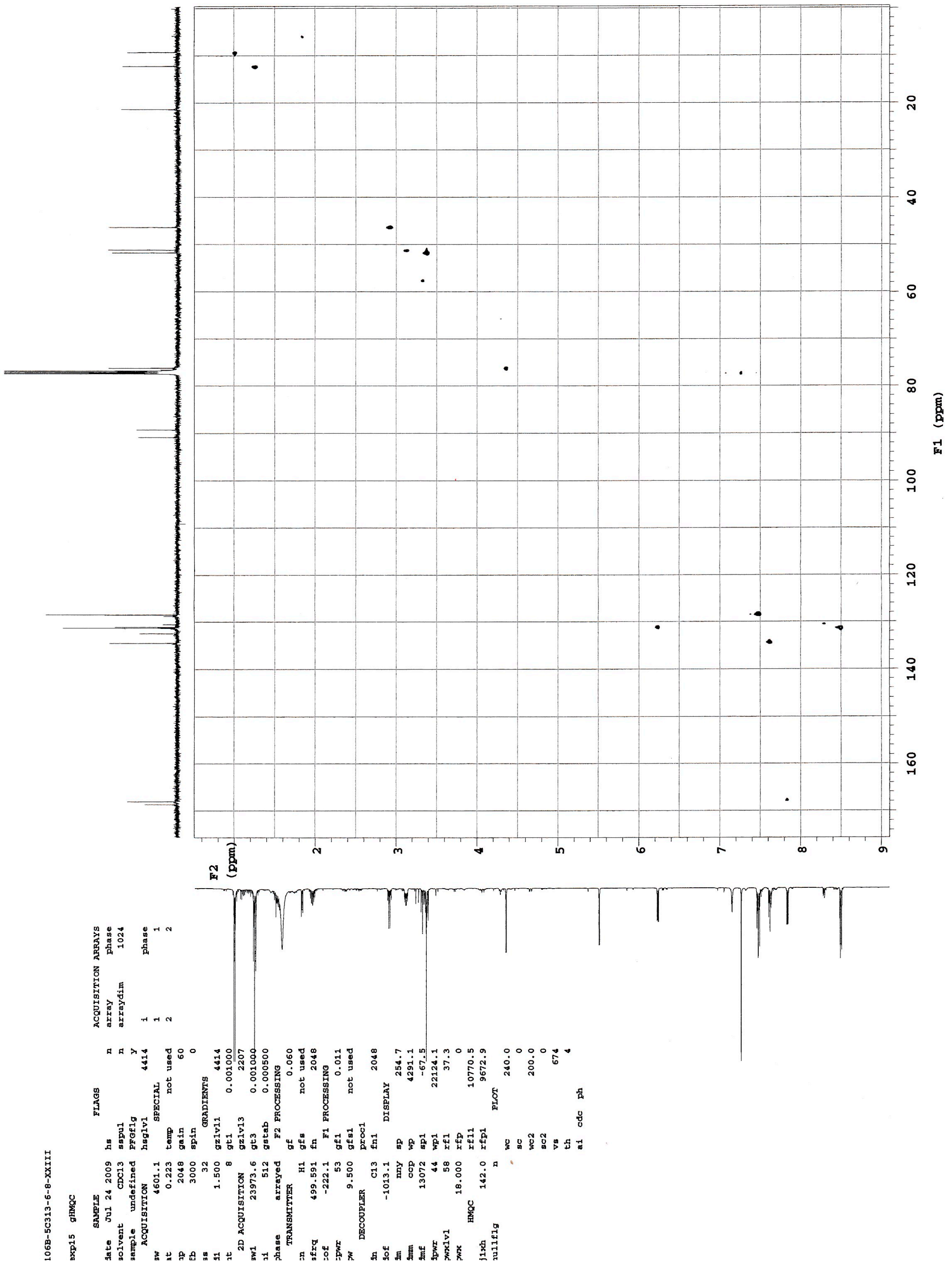

Figure S19 HMBC of **3**

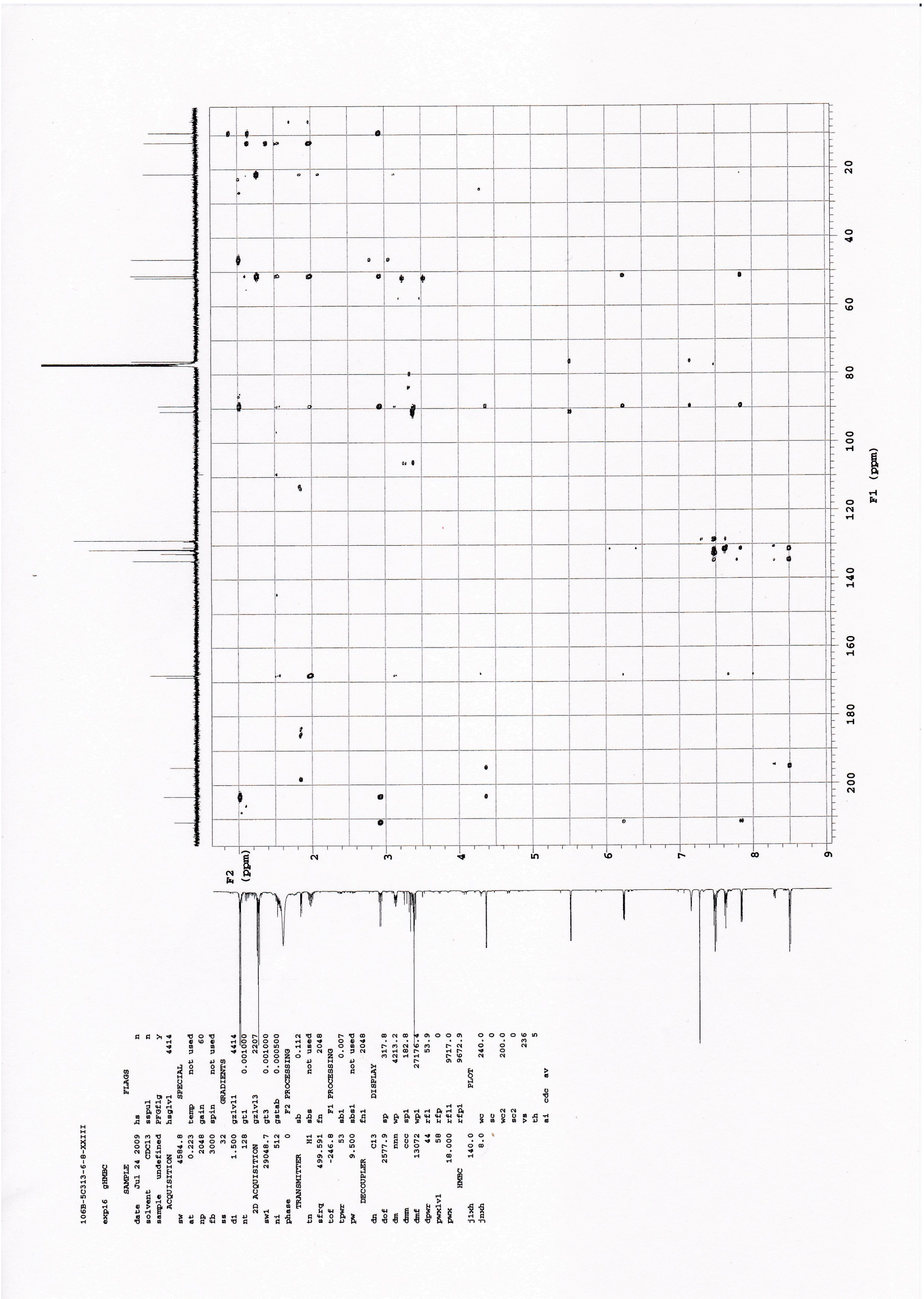

Figure S20 IR spectrum of **3**

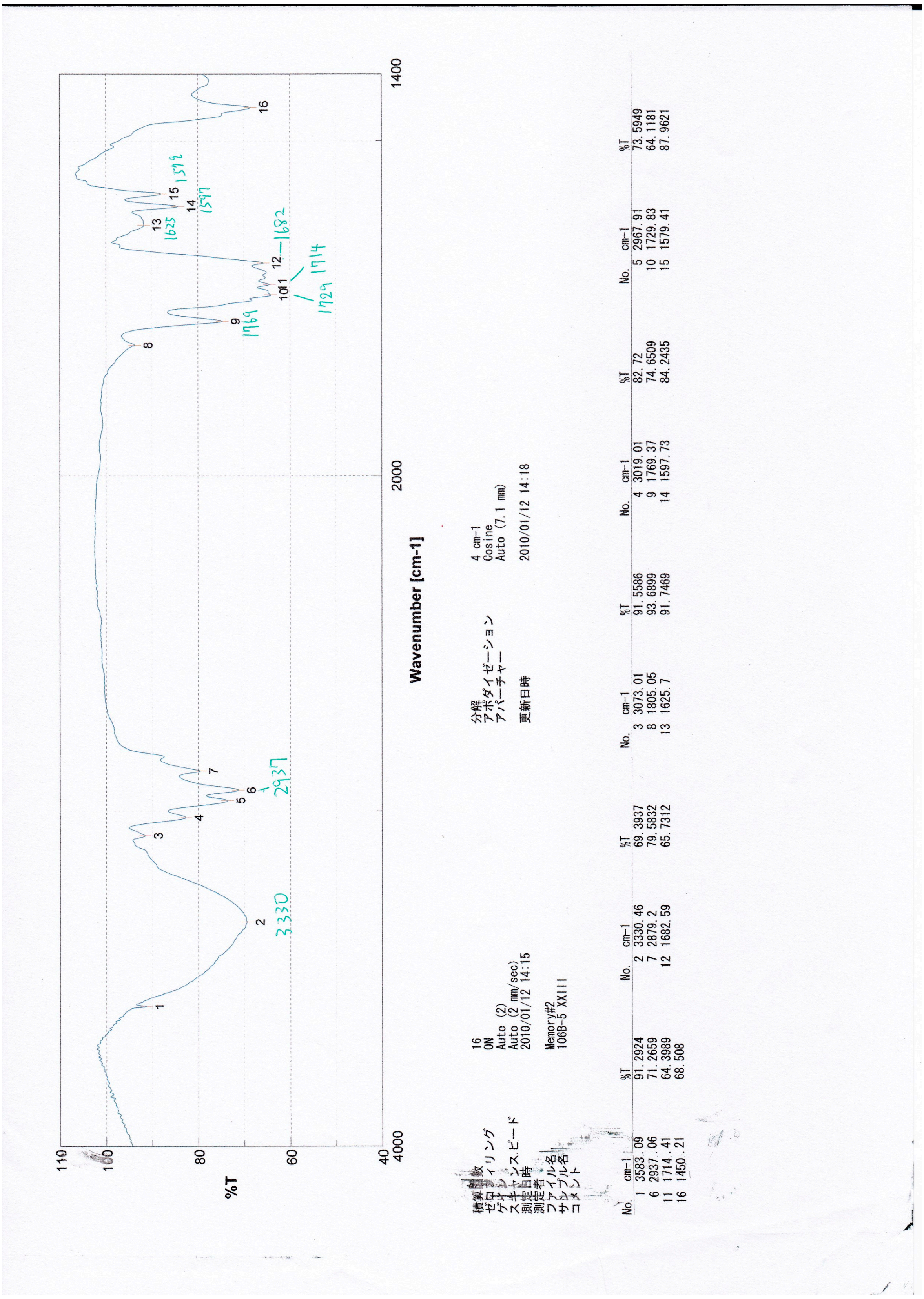

Figure S21 FABMS of **3**

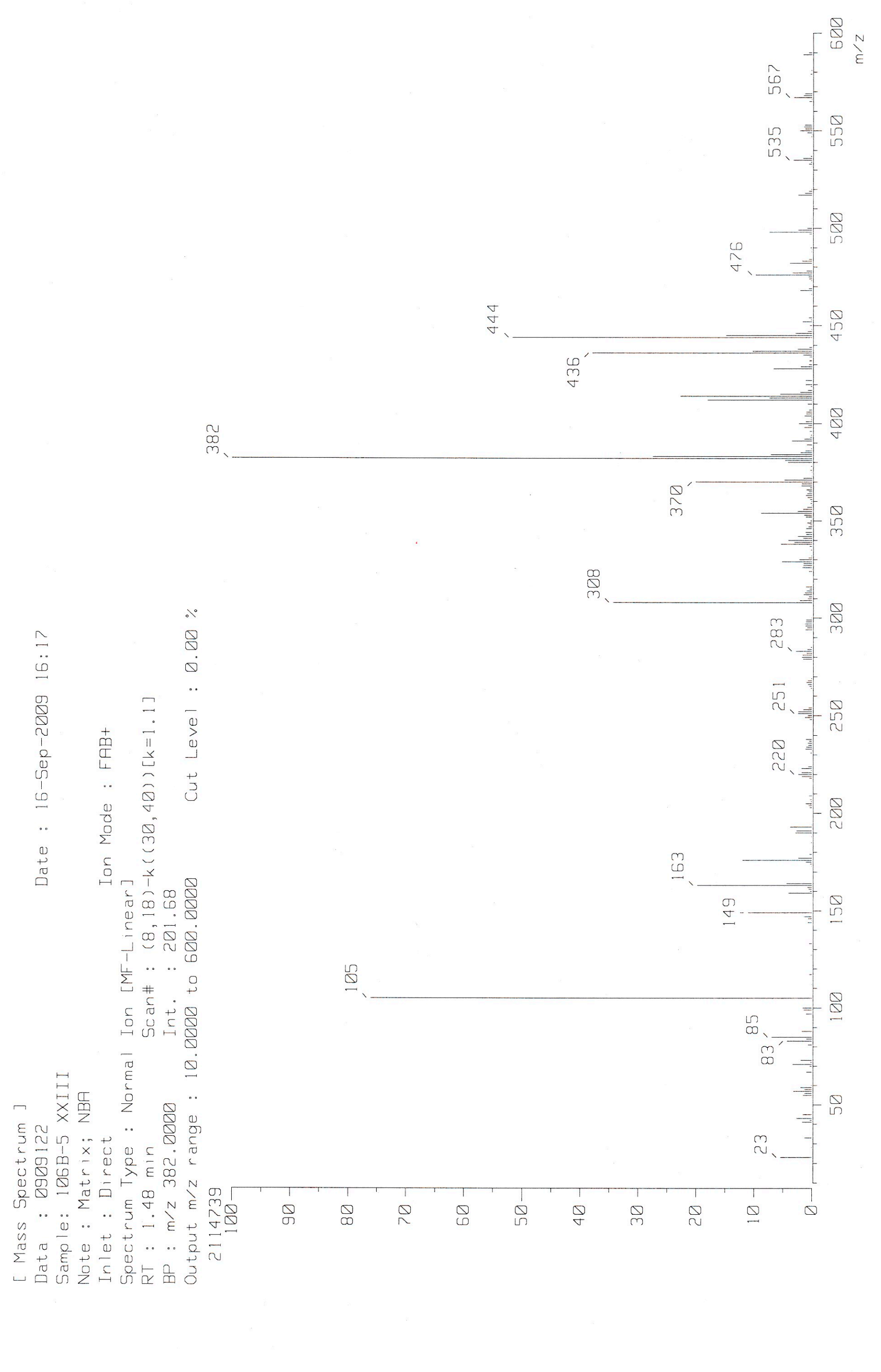

Figure S22 ^{1}H and ^{13}C NMR spectra of **4** in $CDCl_3$

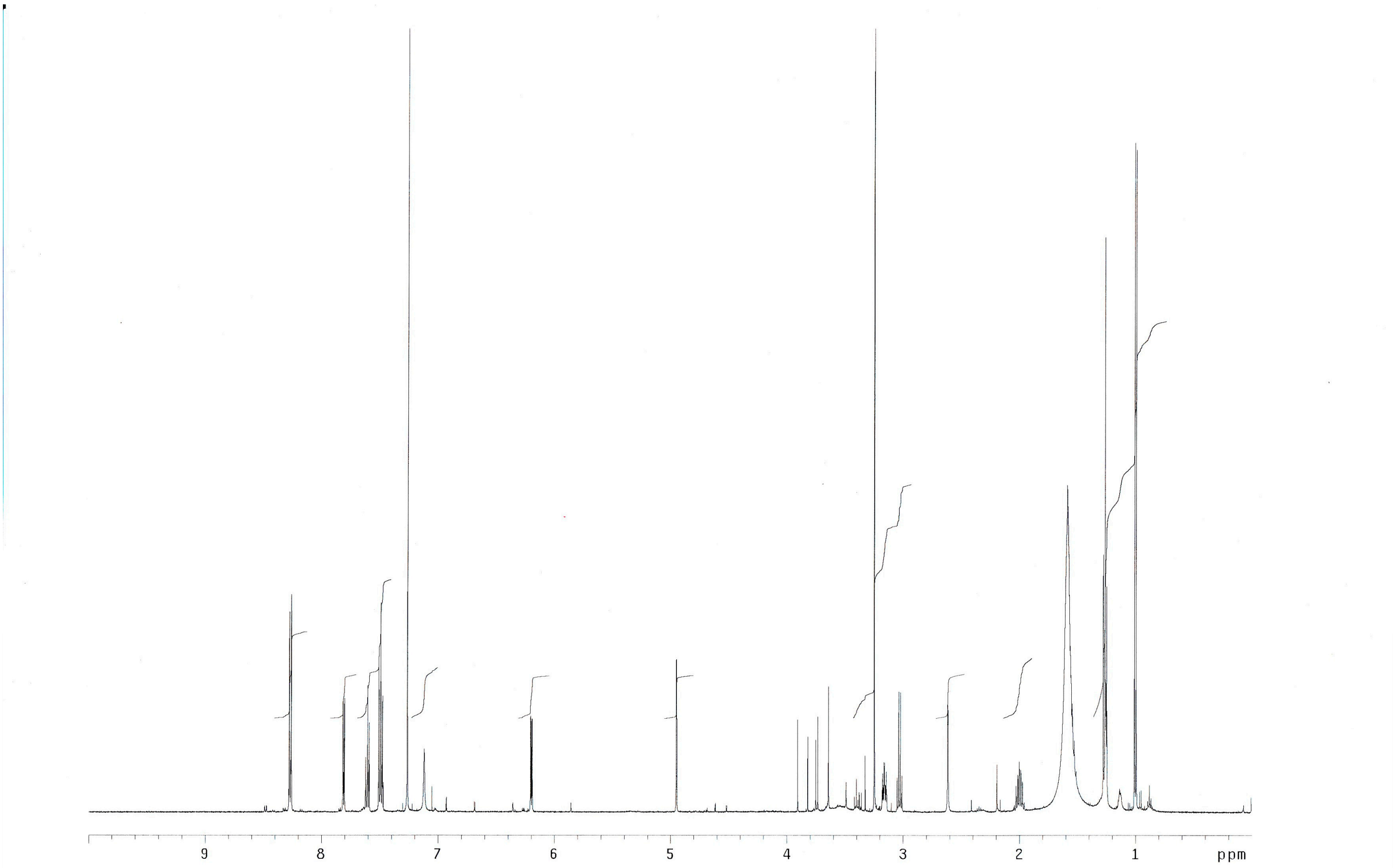

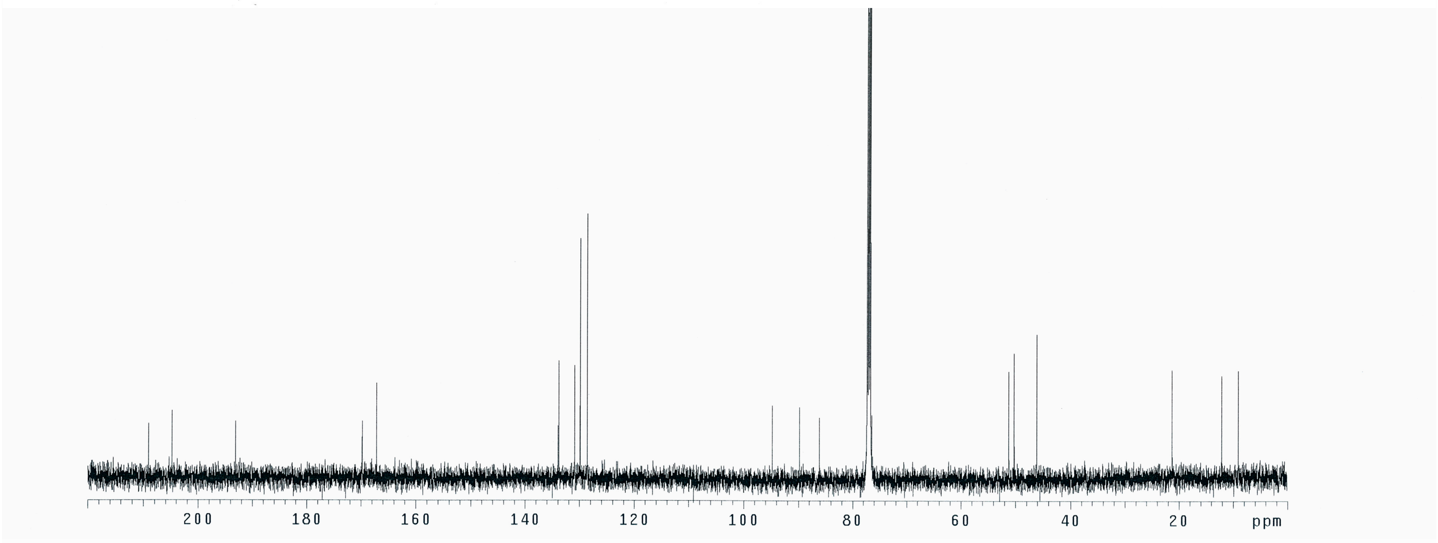

Figure S23 ^{1}H-^{1}H COSY of **4**

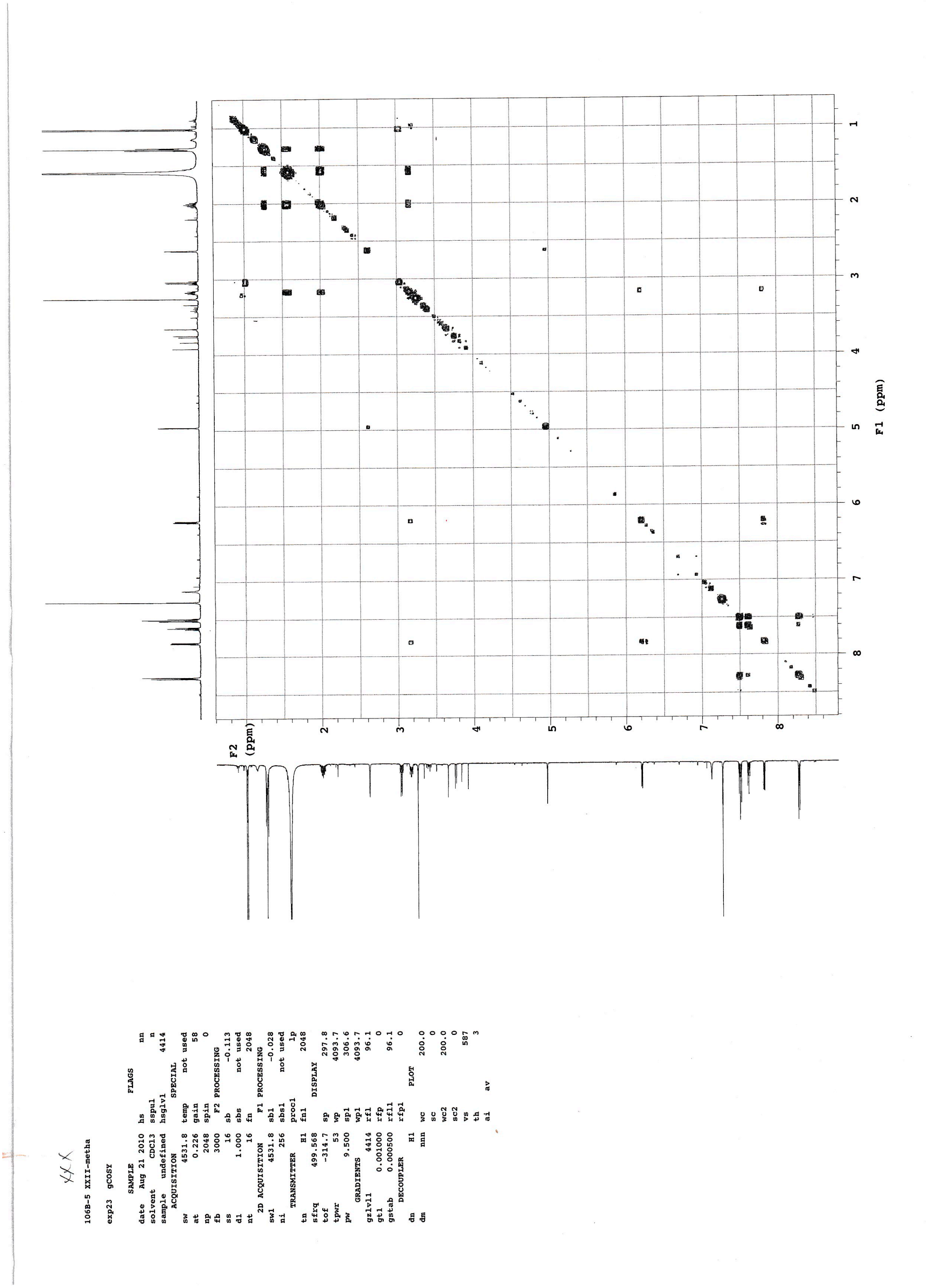

Figure S24 NOESY of **4**

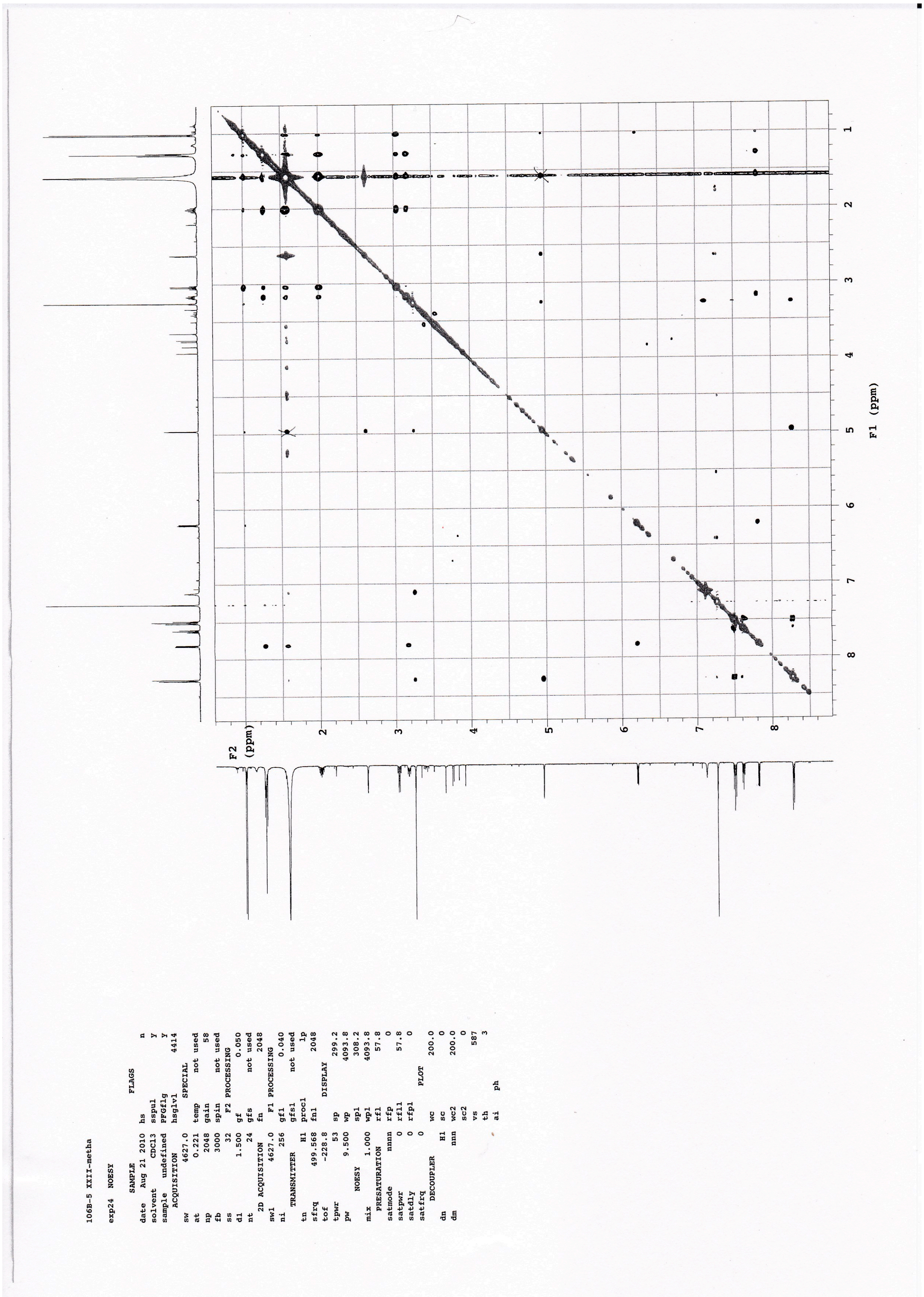

Figure S25 HMQC of **4**

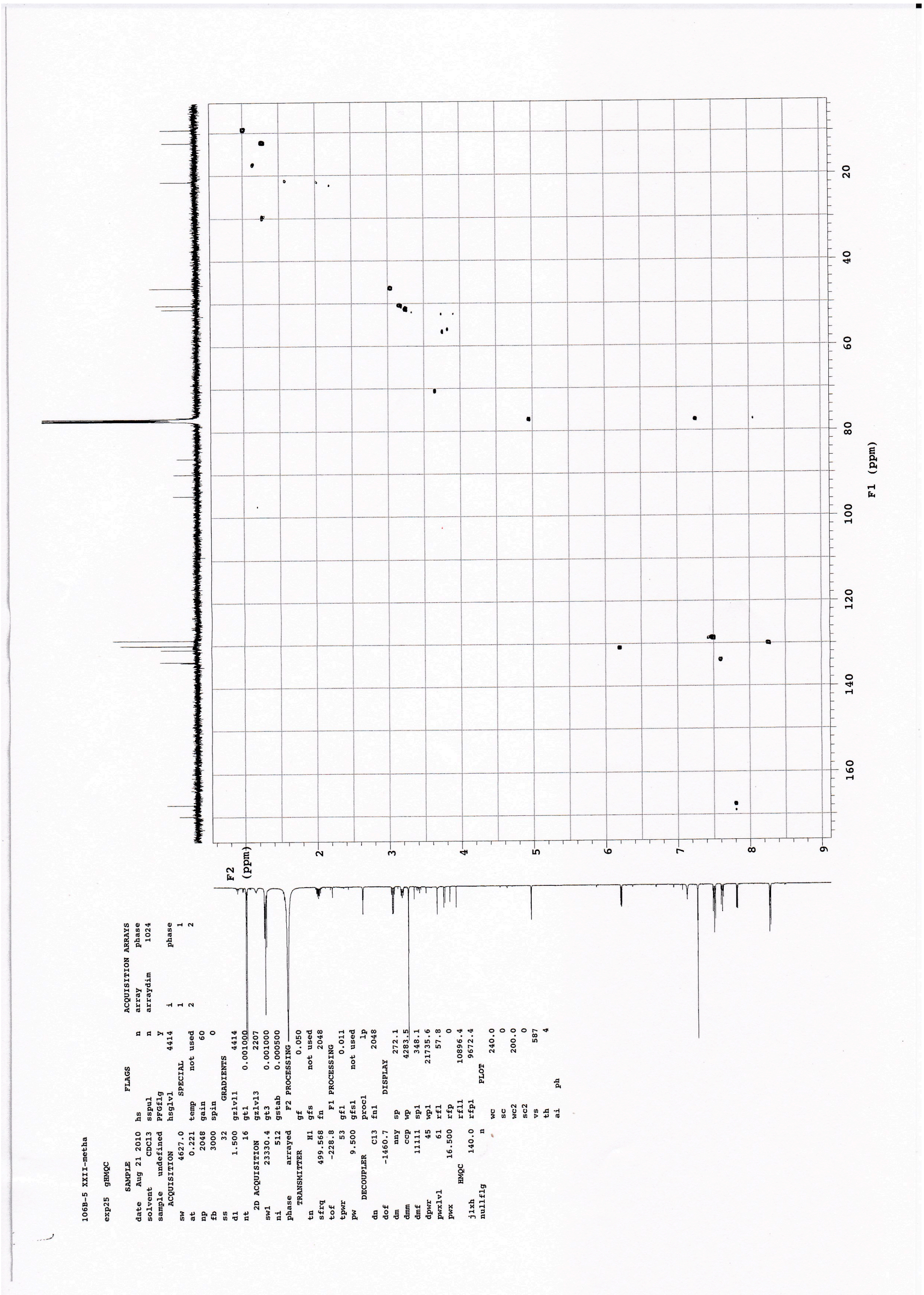

Figure S26 HMBC of **4**

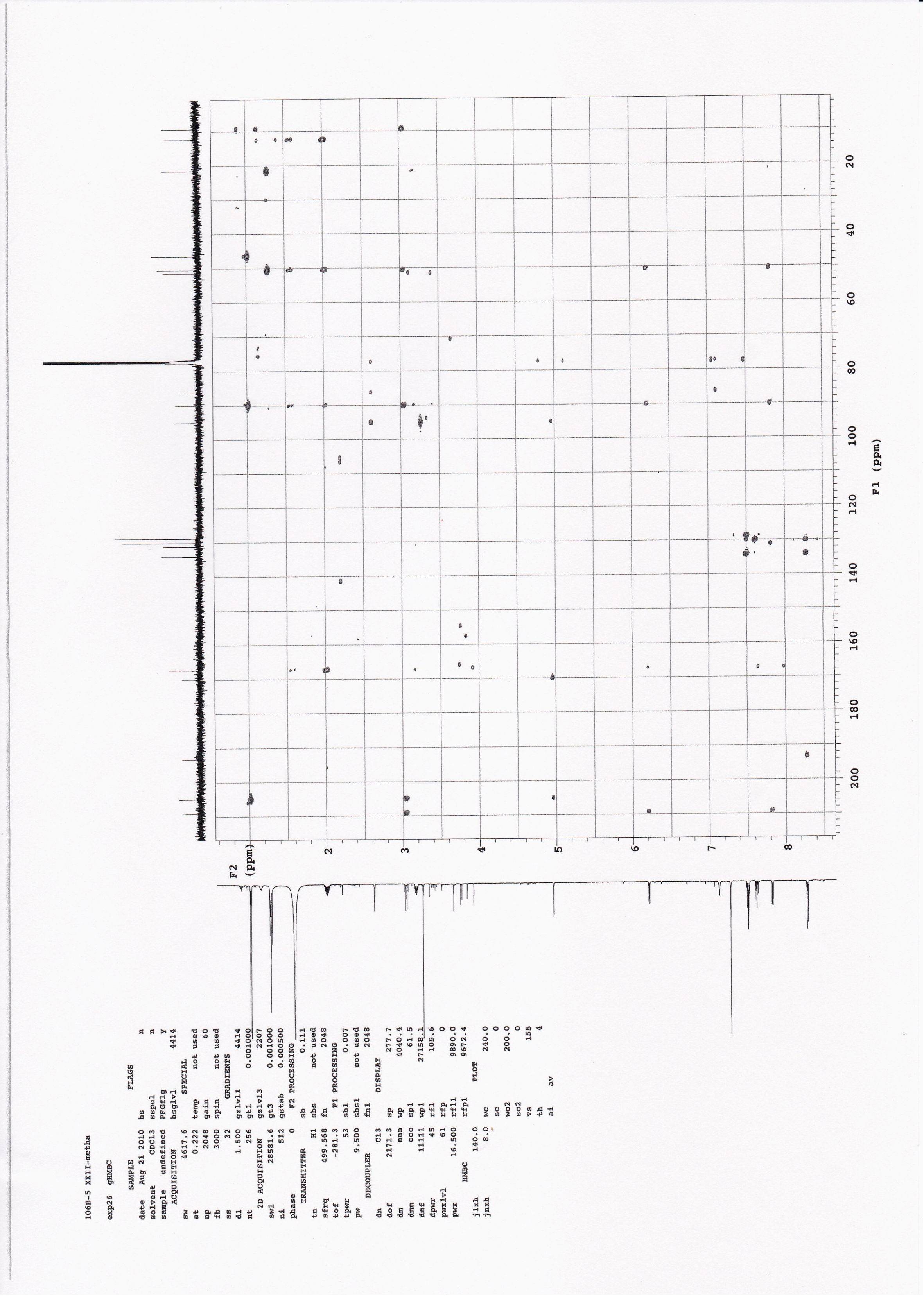

Figure S27 IR spectrum of **4**

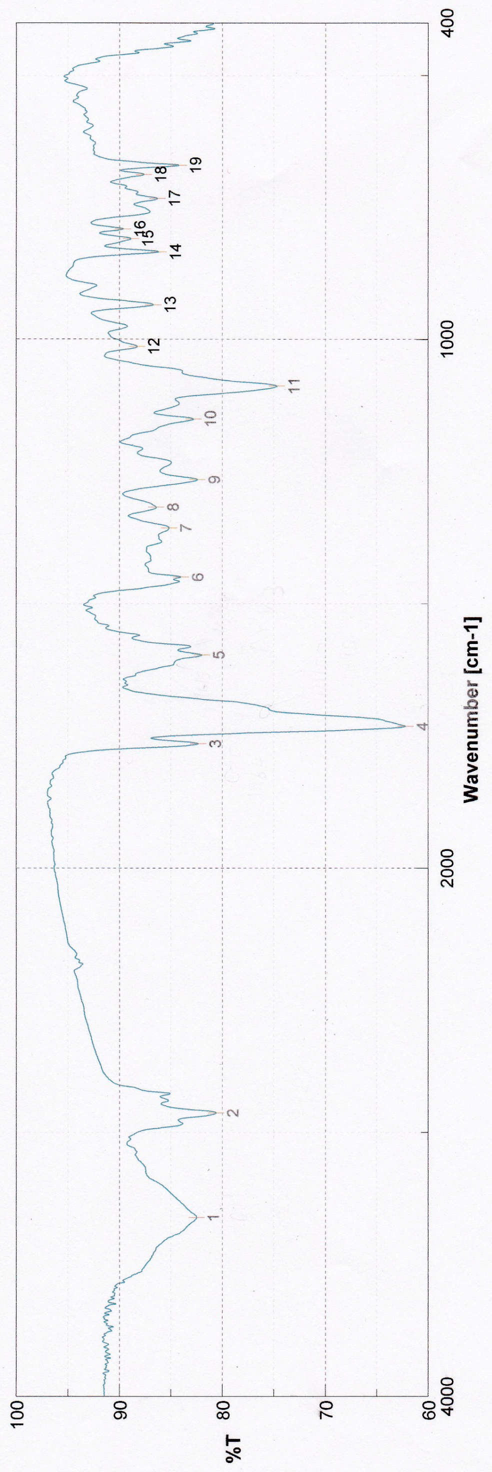

積算回数	16	分解	4 cm-1
ゼロフィリング	ON	アポダイゼーション	Cosine
ゲイン	Auto (2)	アパーチャー	Auto (7.1 mm)
スキャンスピード	Auto (2 mm/sec)		
測定日時	2011/01/19 15:20	更新日時	2011/01/19 16:02
測定者	miyamoto		
ファイル名	C479-7-9 XXX		
サンプル名	106B-5 C479-7-9 XXX		
コメント			

No.	cm-1	%T	No.	cm-1	%T	No.	cm-1	%T	No.	cm-1	%T	No.	cm-1	%T
1	3321.78 OH	82.4847	2	2925.48 NH	80.6128	3	1764.55 C=O	82.3144	4	1731.76 C=O	62.2001	5	1596.77	81.9741
6	1449.24	84.019	7	1356.68	85.1626	8	1317.14	86.3872	9	1265.07	82.3958	10	1150.33	82.7664
11	1087.66	74.667	12	1013.41	88.2701	13	934.342	86.6659	14	834.062	86.166	15	808.992	88.8595
16	790.671	89.647	17	732.817	86.28	18	687.498	87.6224	19	670.142	84.2458			

Figure S28 FABMS of **4**

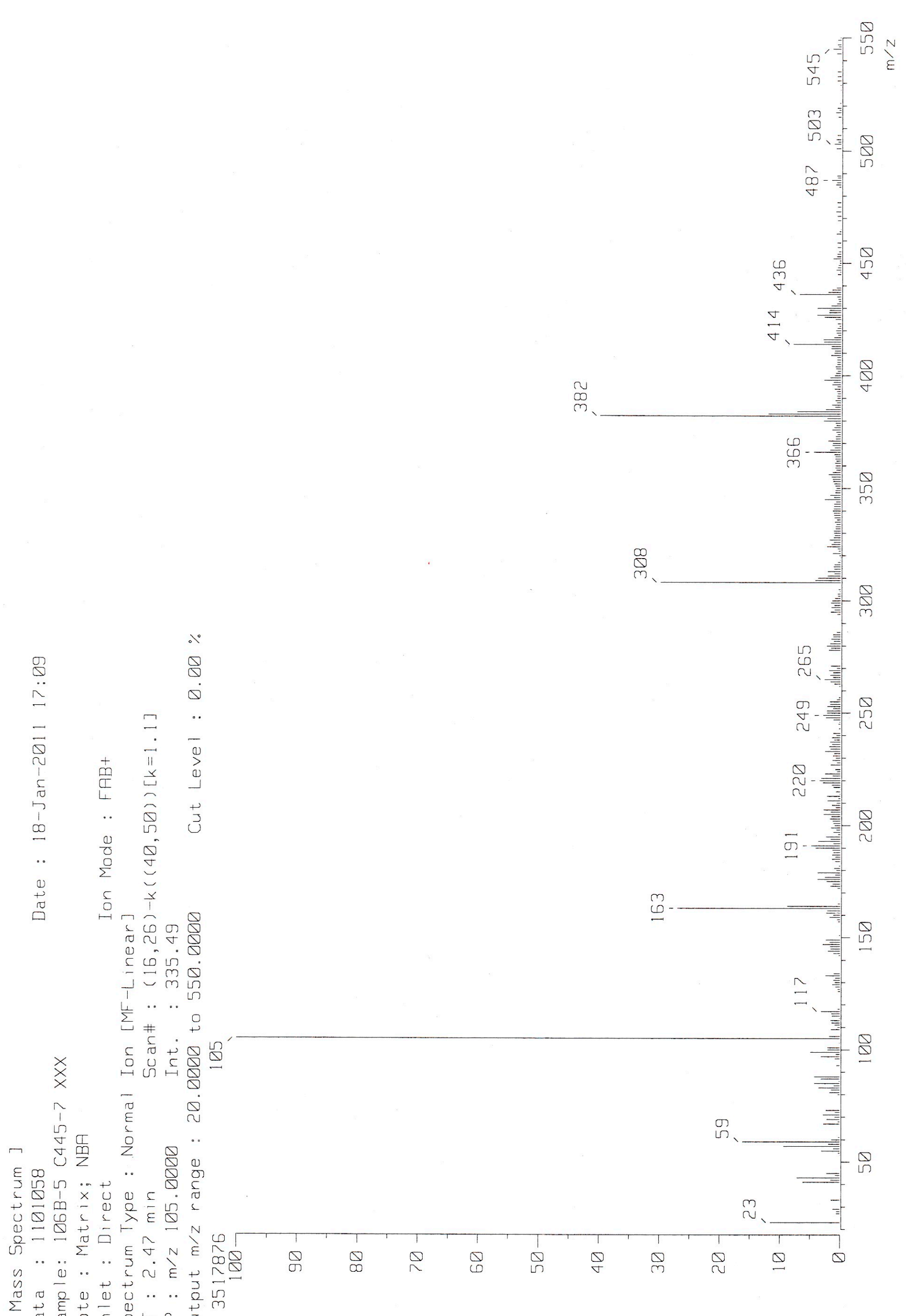

Figure S29 ^{1}H and ^{13}C NMR spectra of **5** in $CDCl_3$

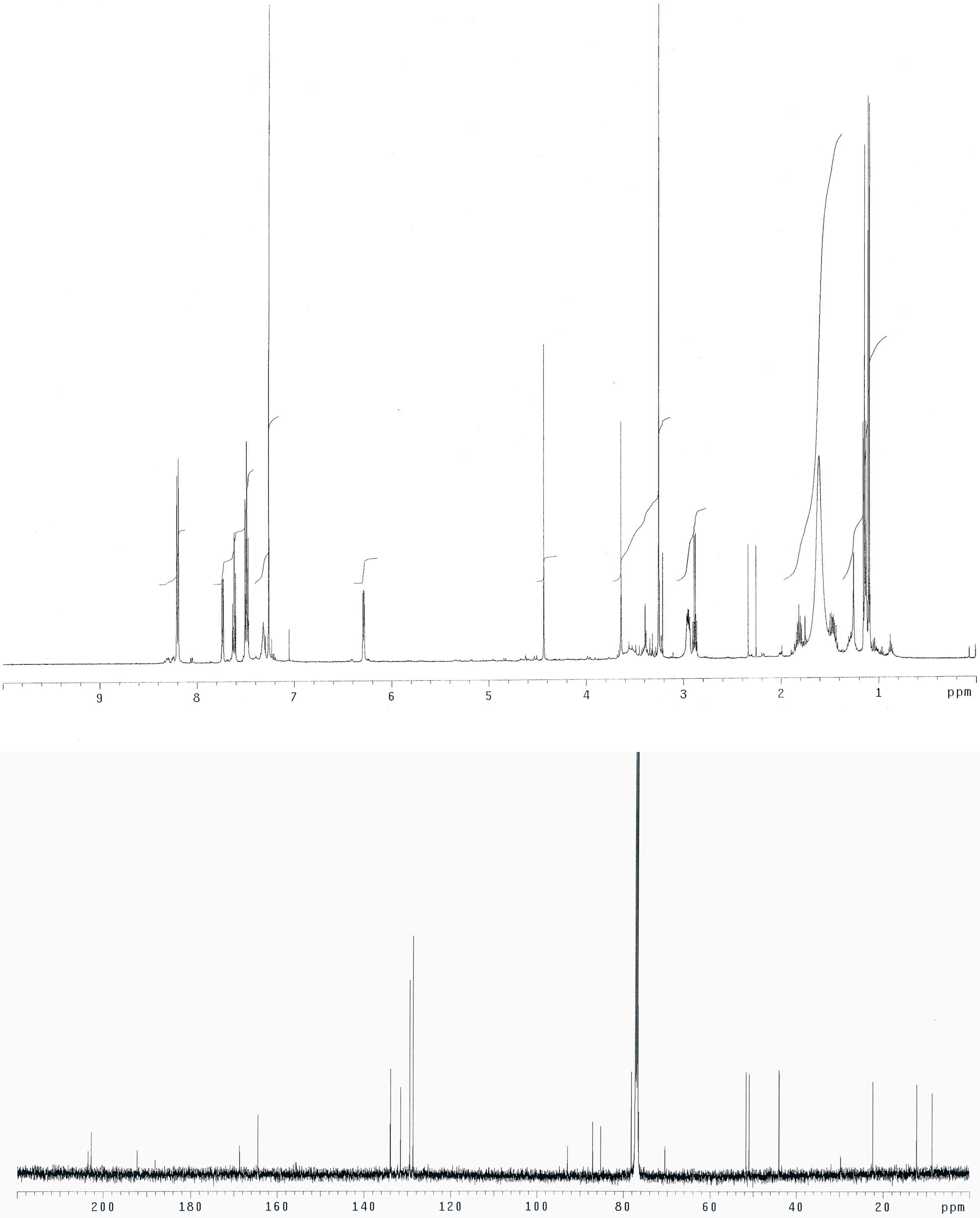

Figure S30 ^{1}H-^{1}H COSY of **5**

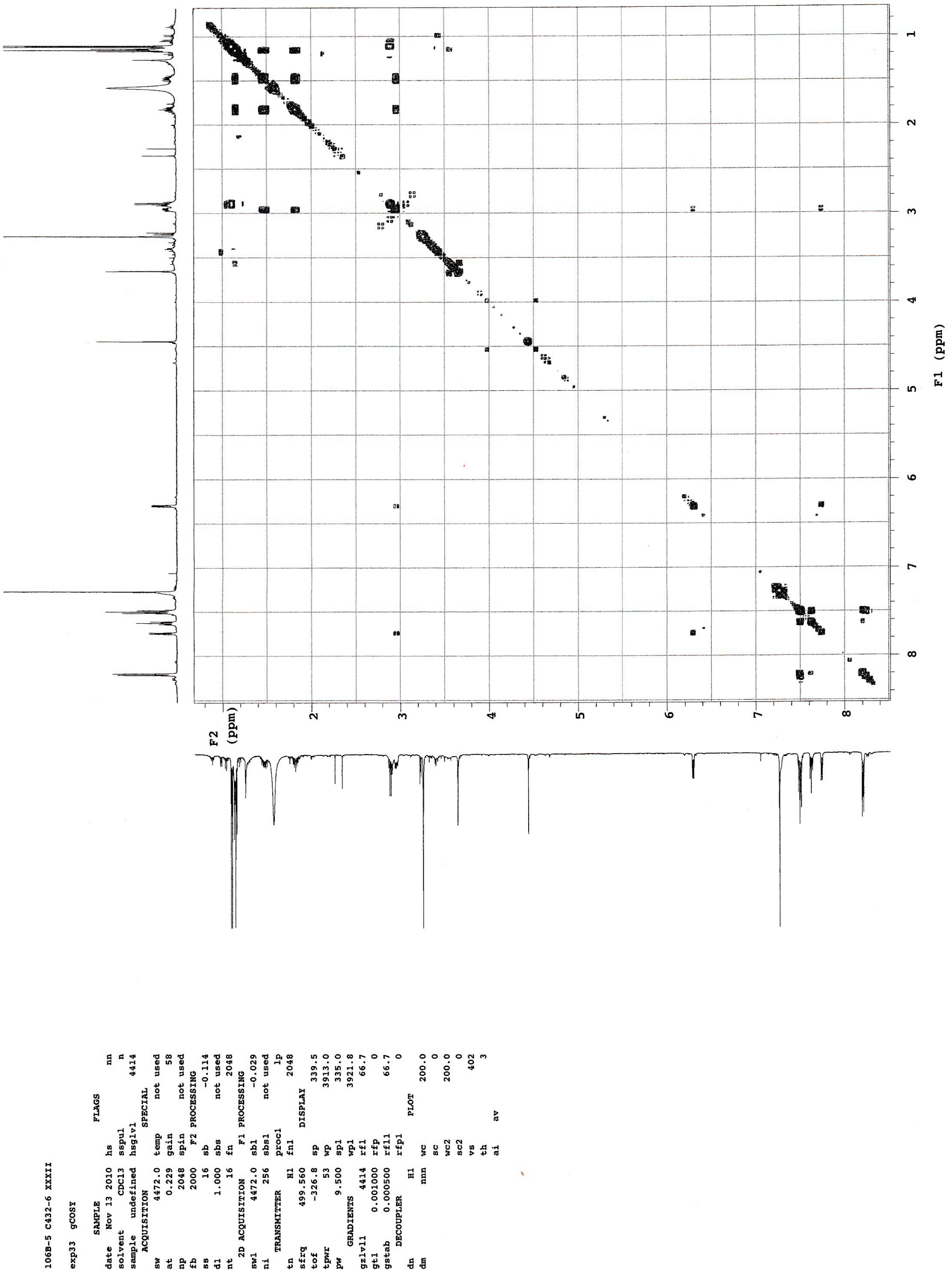

Figure S31 NOESY of **5**

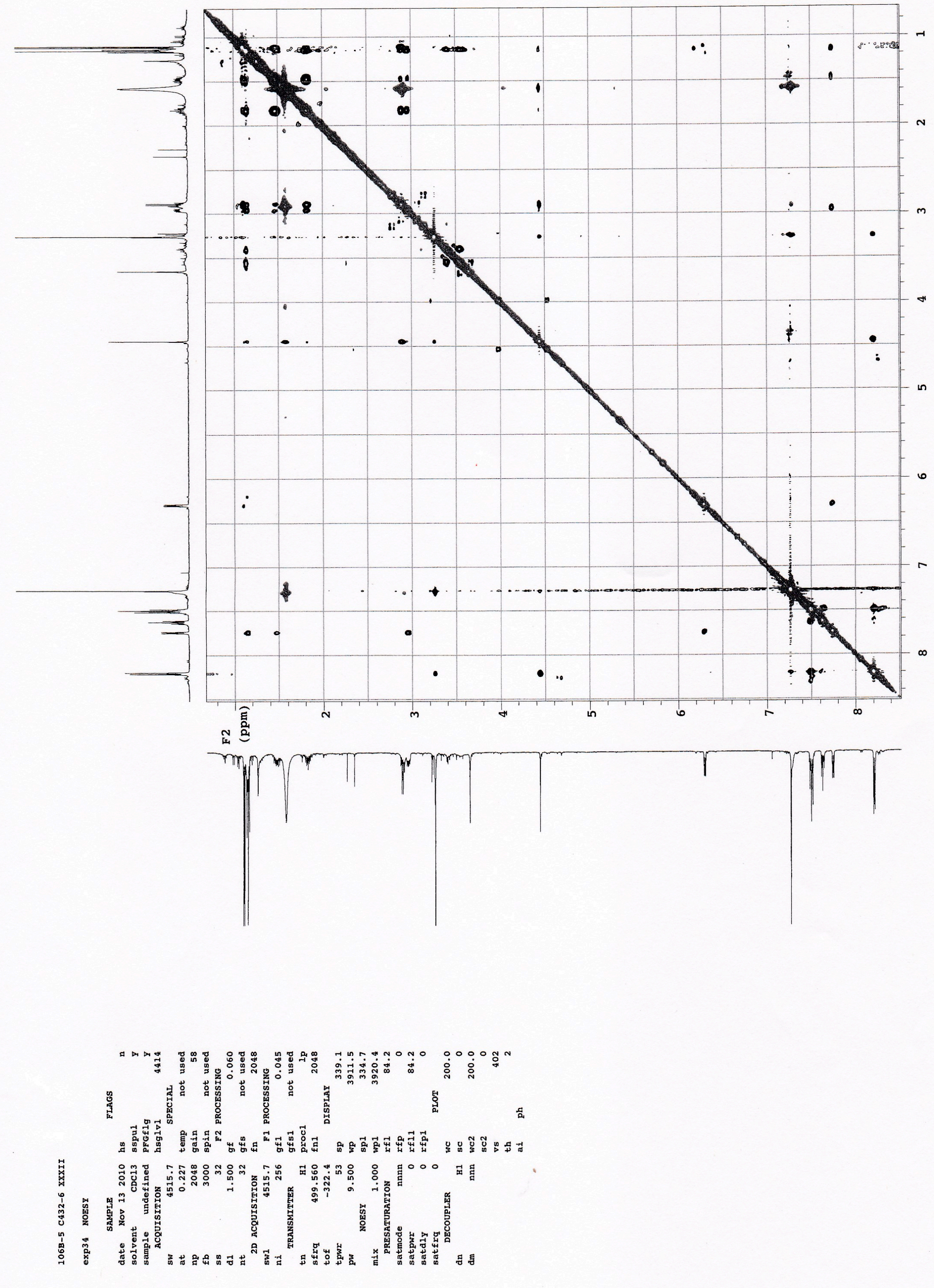

Figure S32 HMQC of **5**

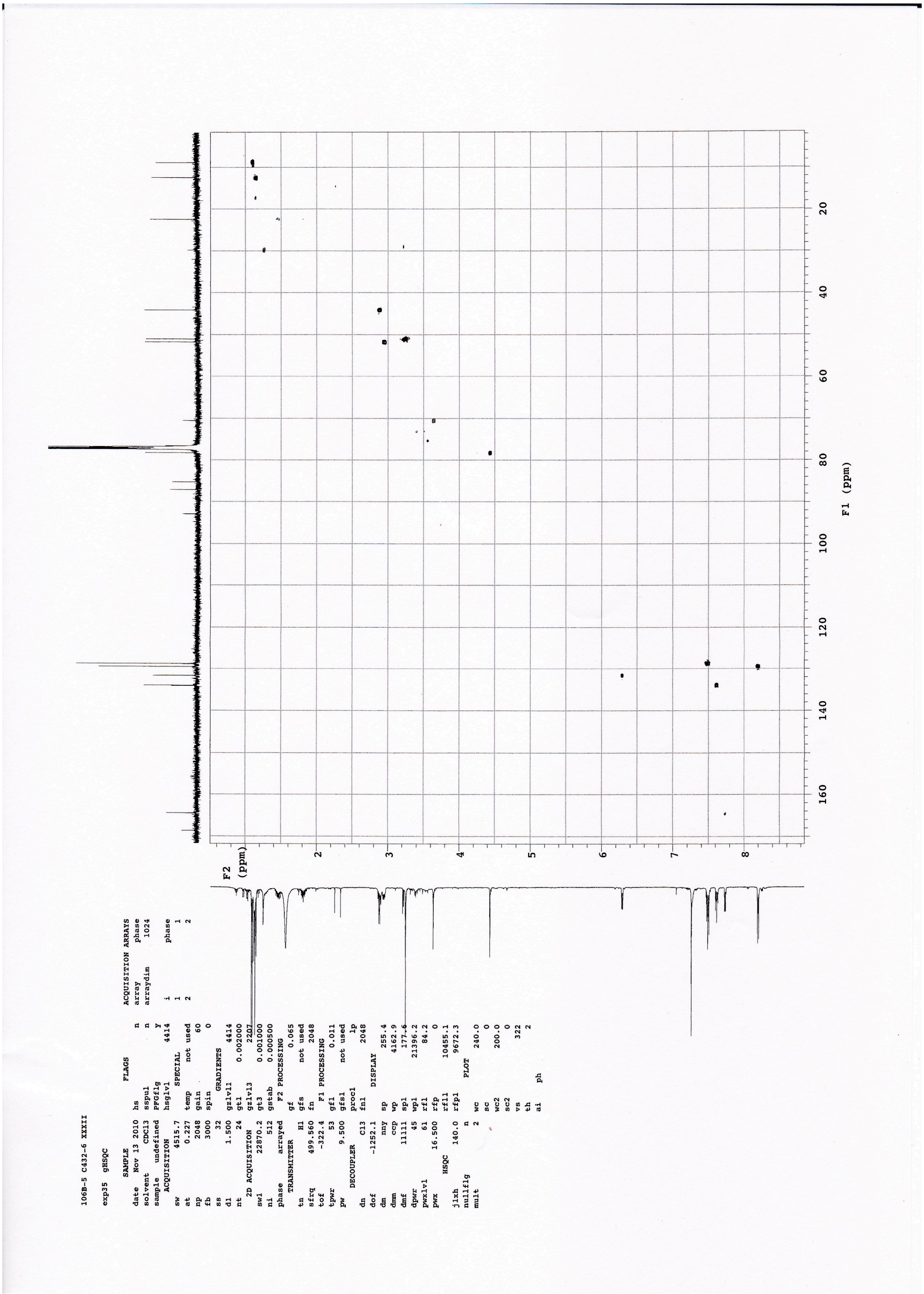

Figure S33 HMBC of **5**

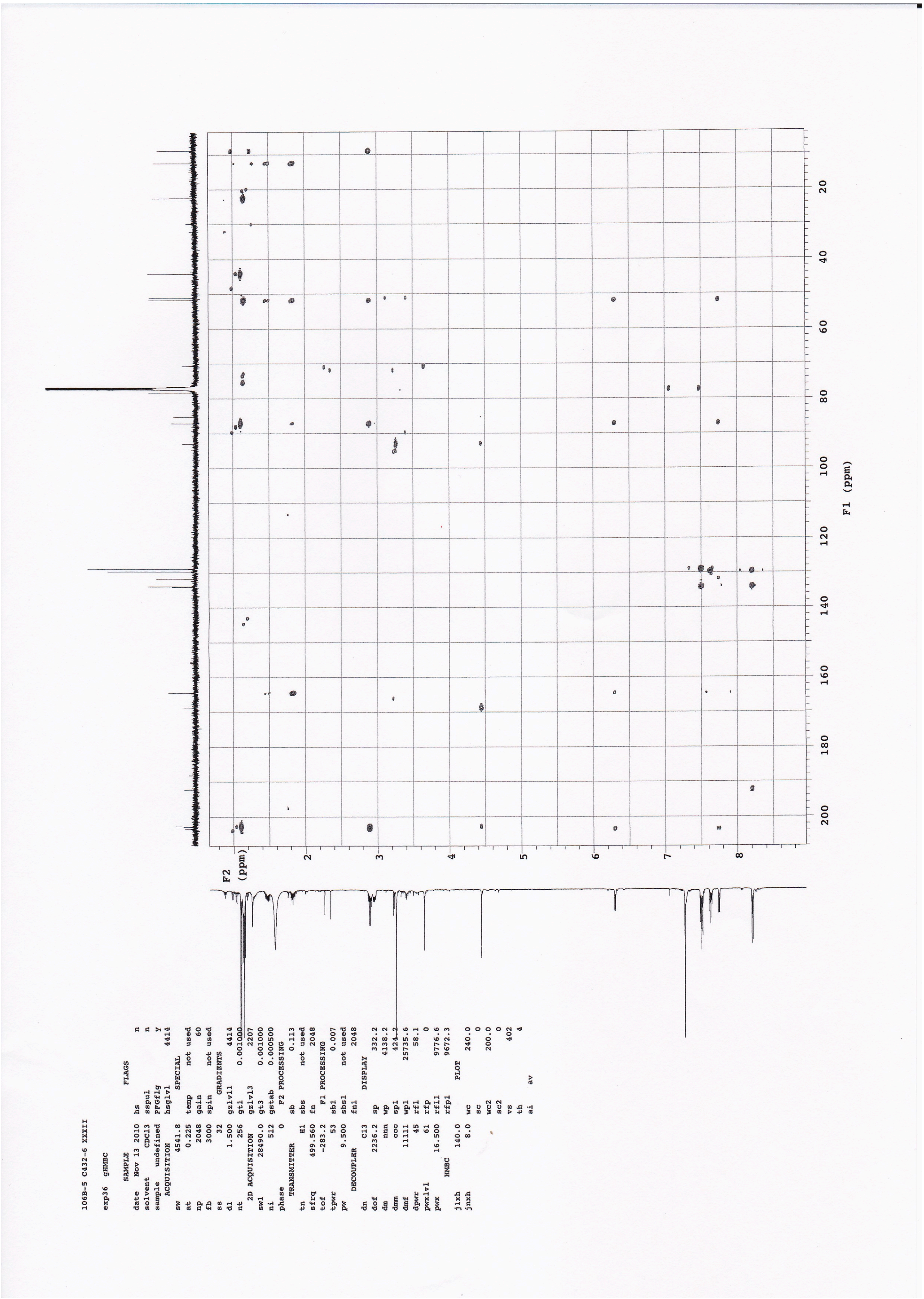

Figure S34 IR spectrum of **5**

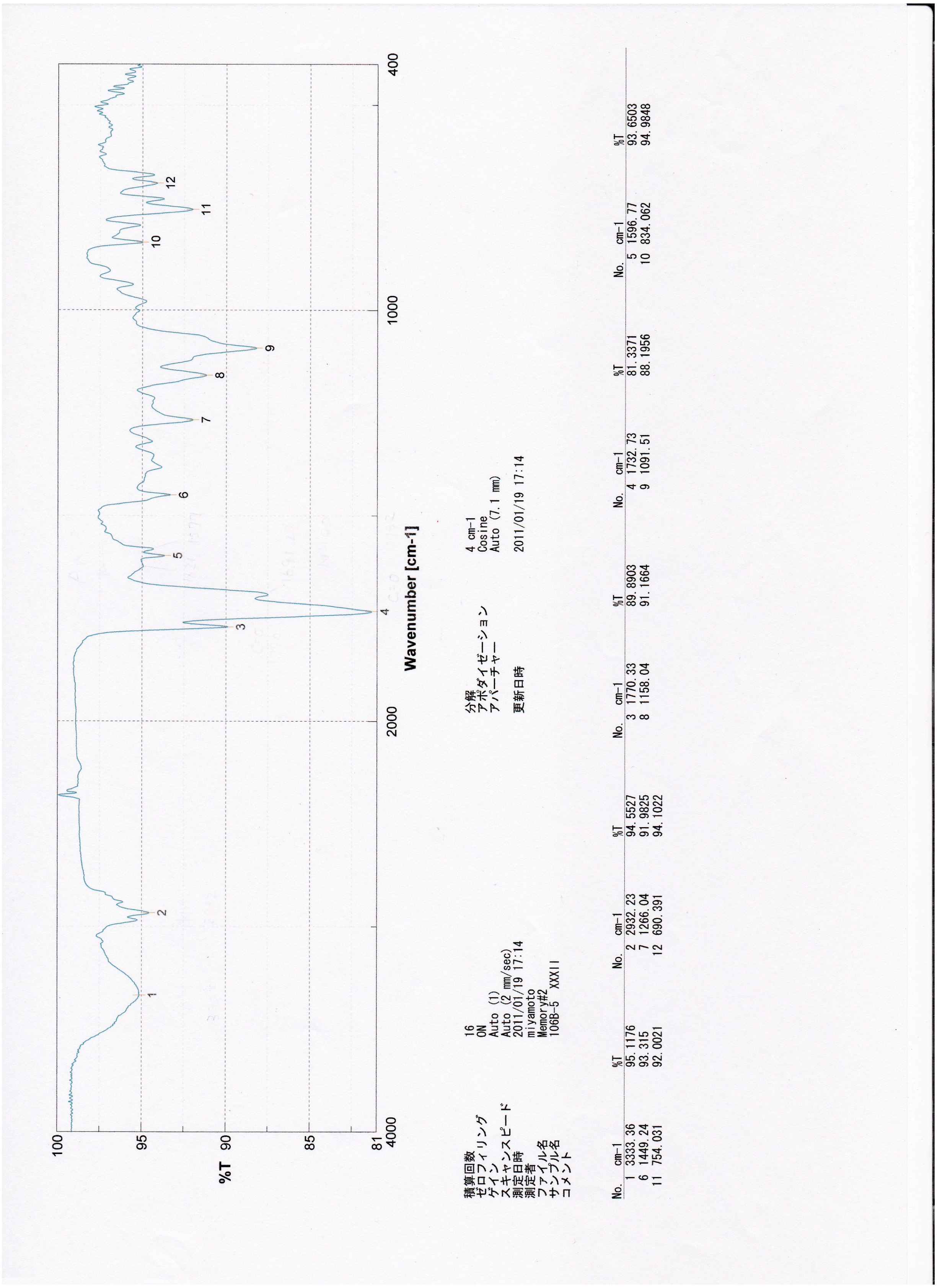

Figure S35 FABMS of **5**

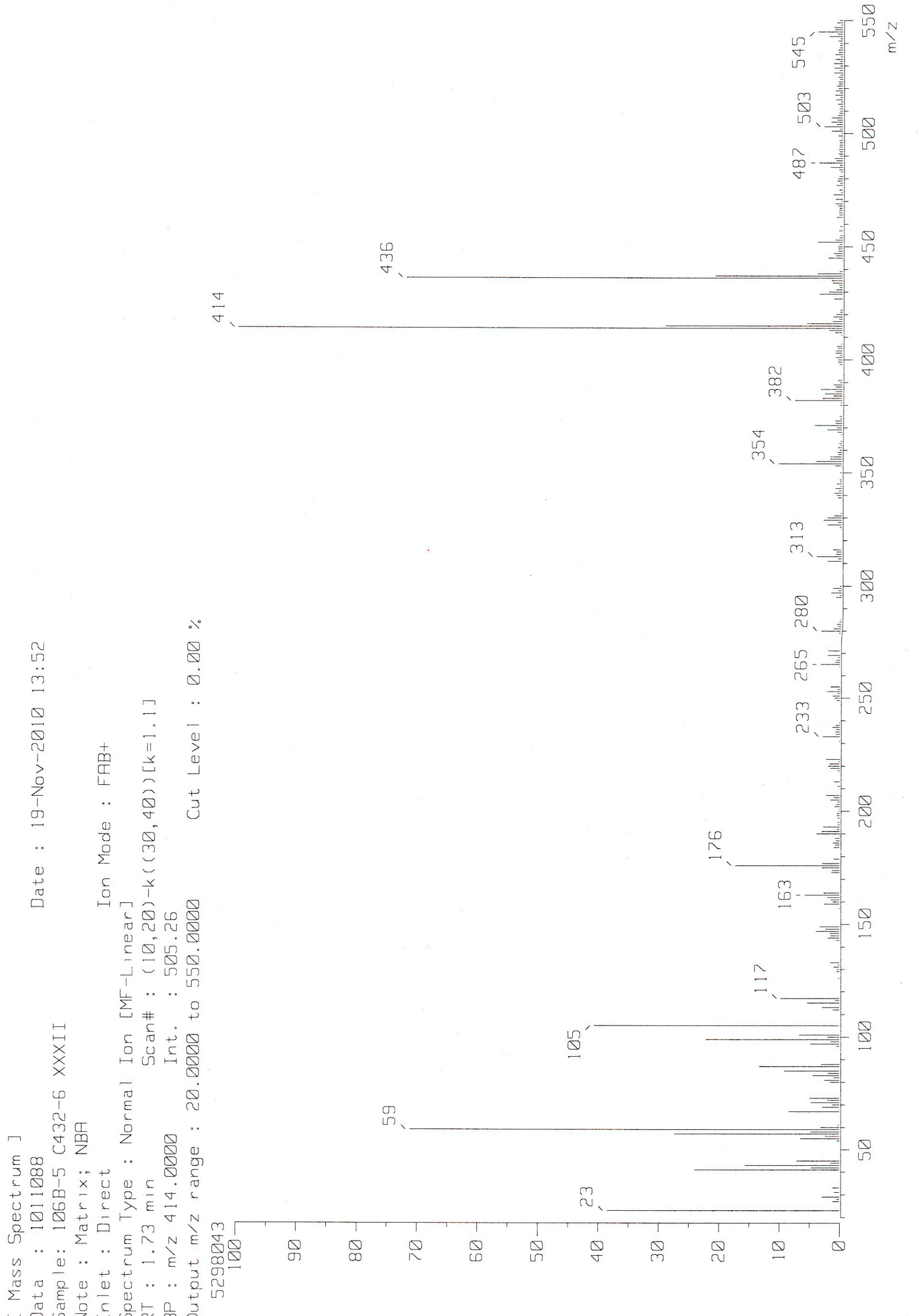

Figure S36 ^{1}H and ^{13}C NMR spectra of **6** in $CDCl_3$

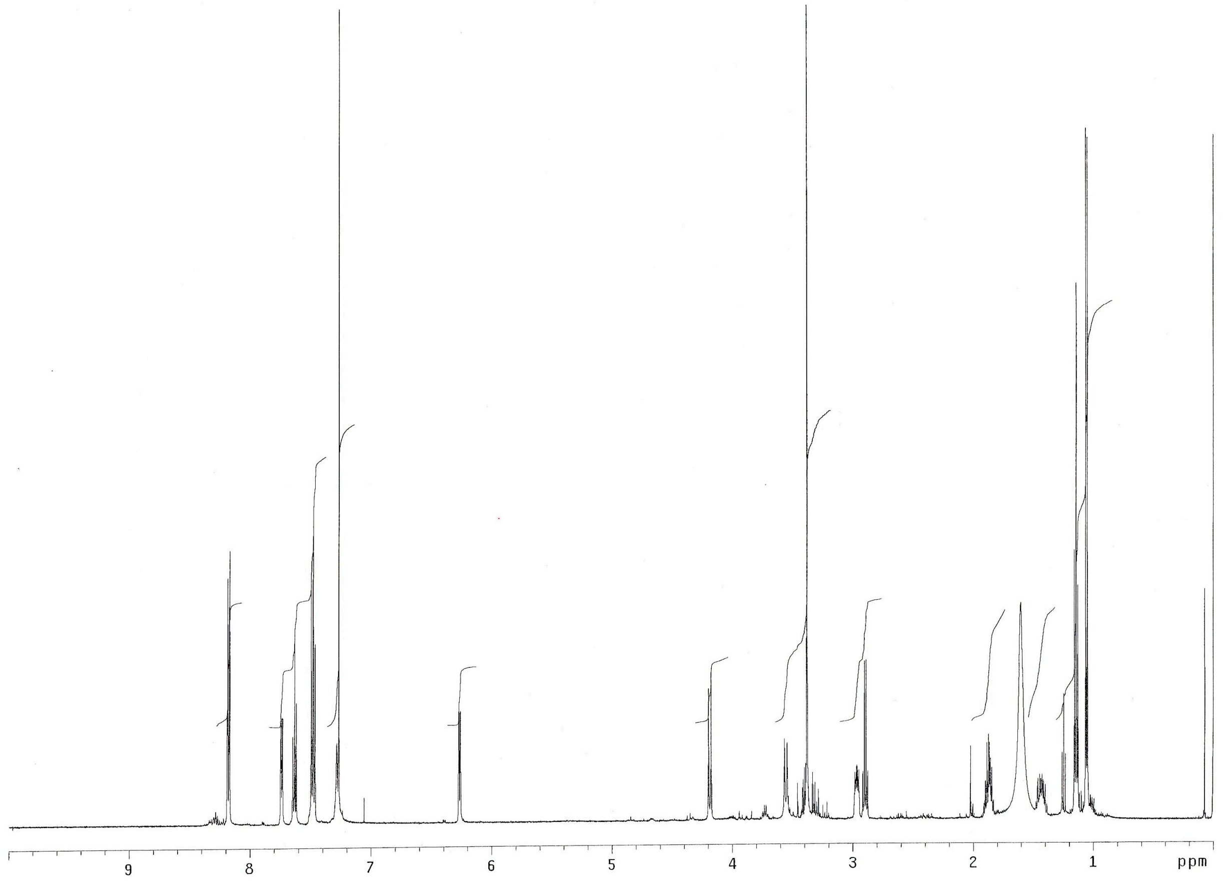

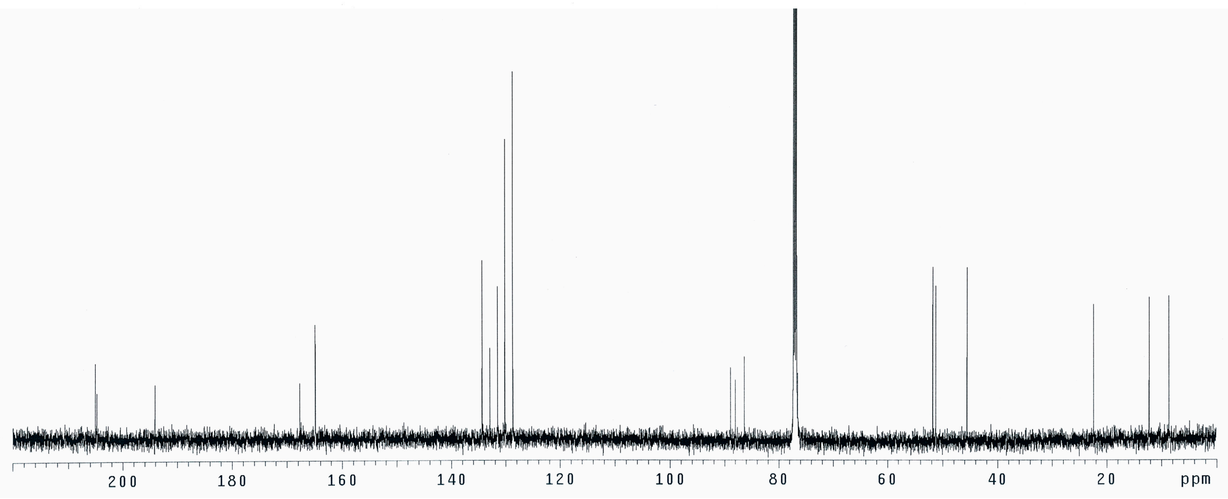

Figure S37 ^{1}H-^{1}H COSY of **6**

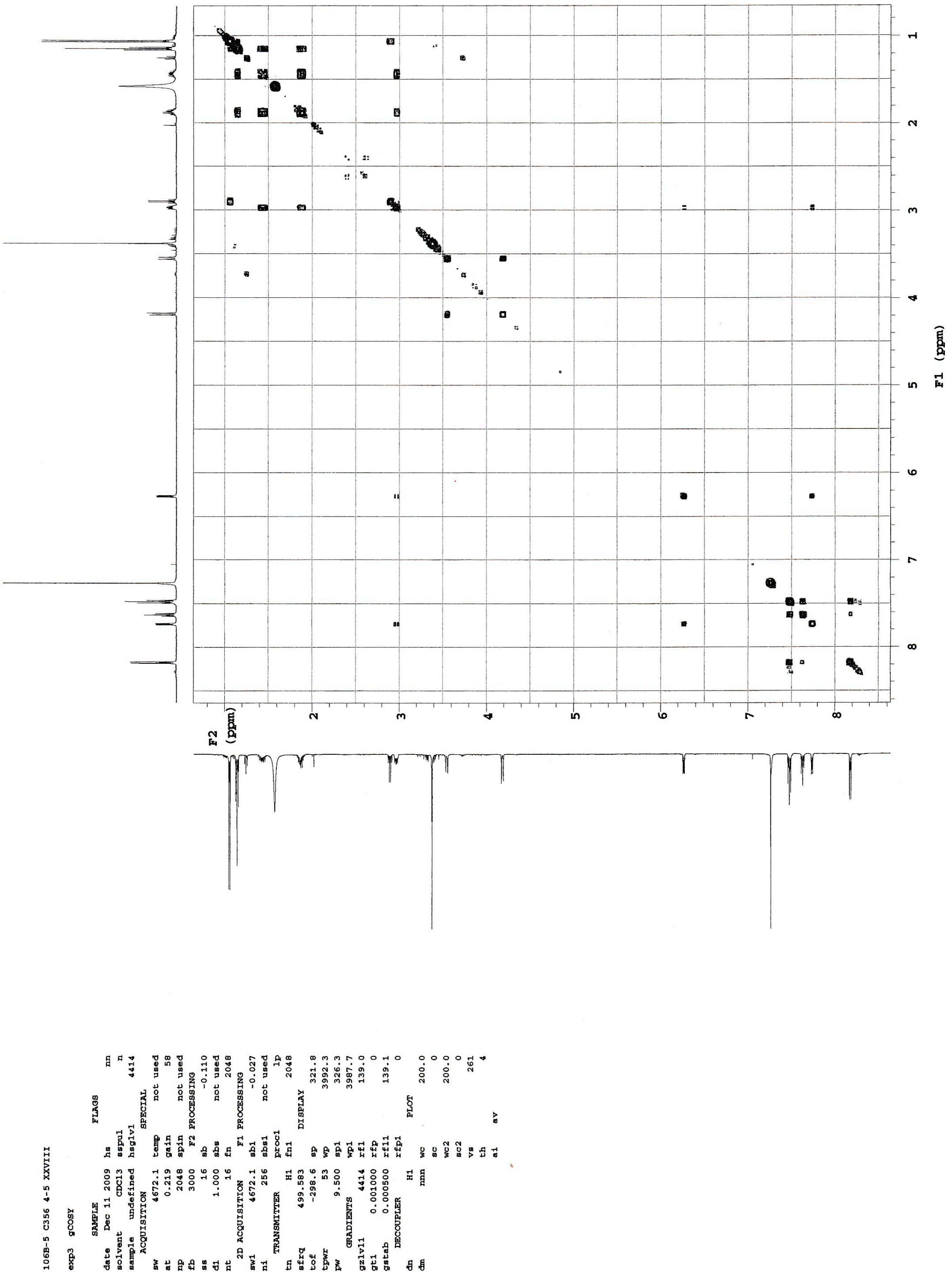

Figure S38 NOESY of **6**

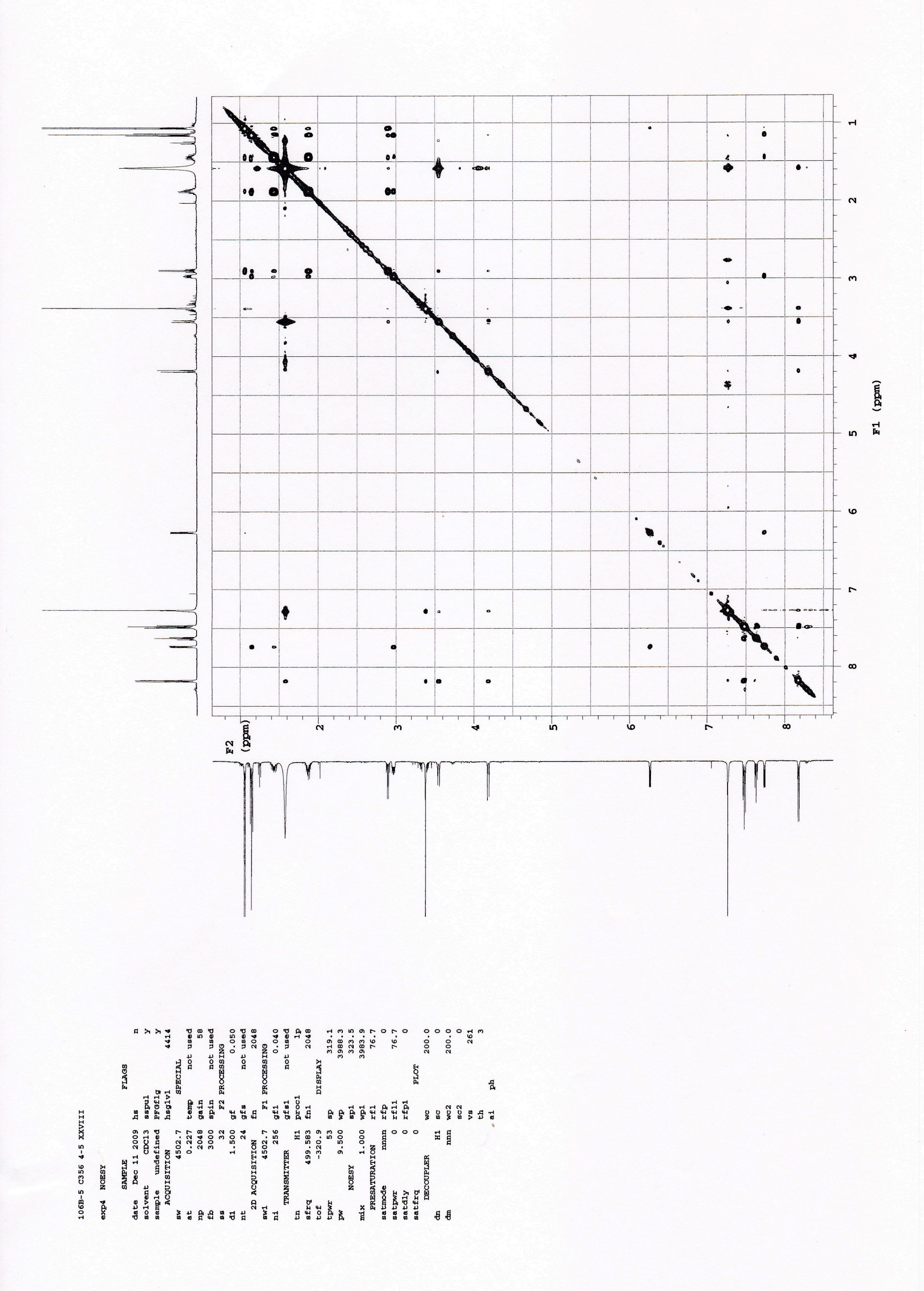

Figure S39 HMQC of **6**

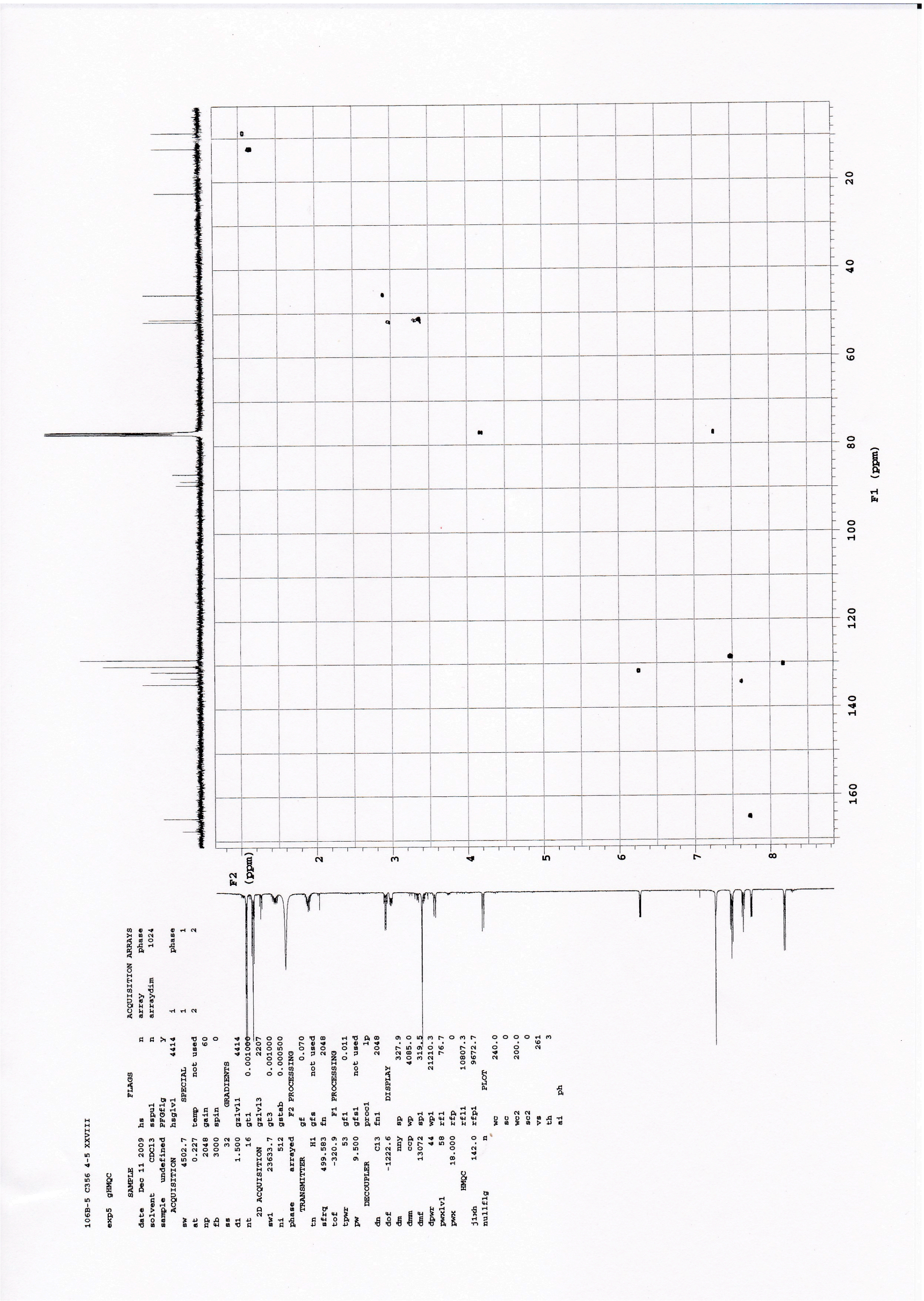

Figure S40 HMBC of **6**

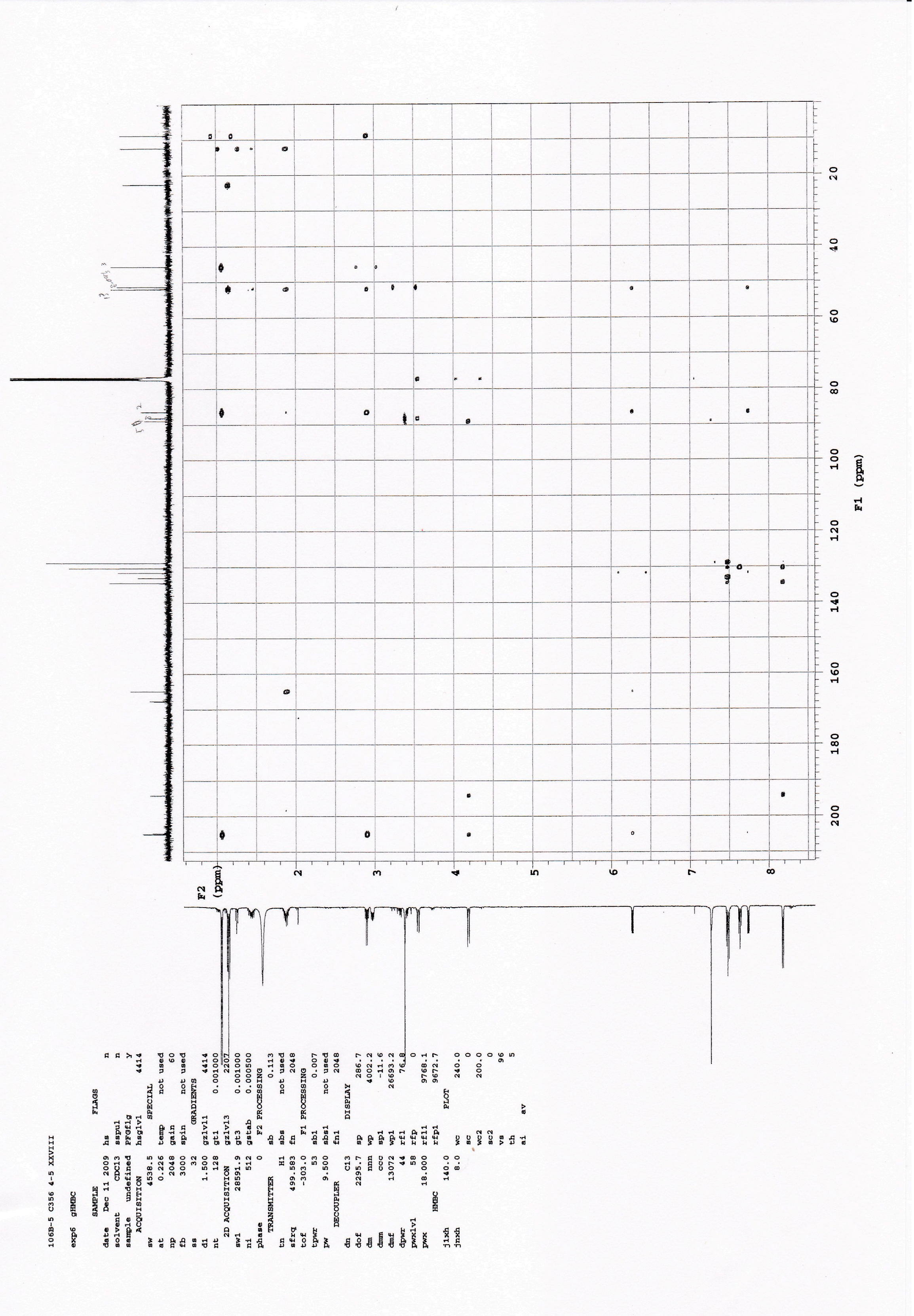

Figure S41 IR spectrum of **6**

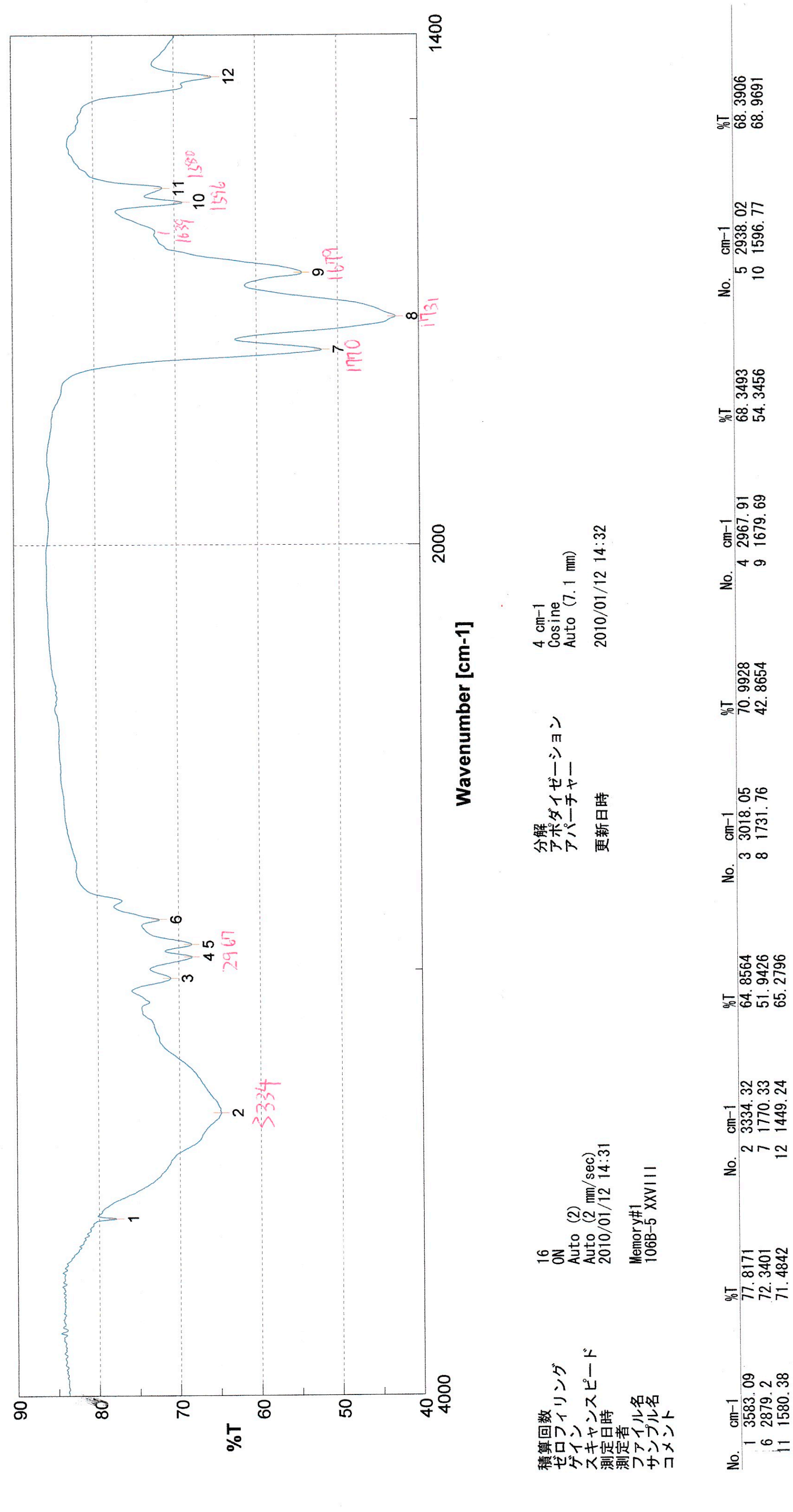

Figure S42 FABMS of **6**

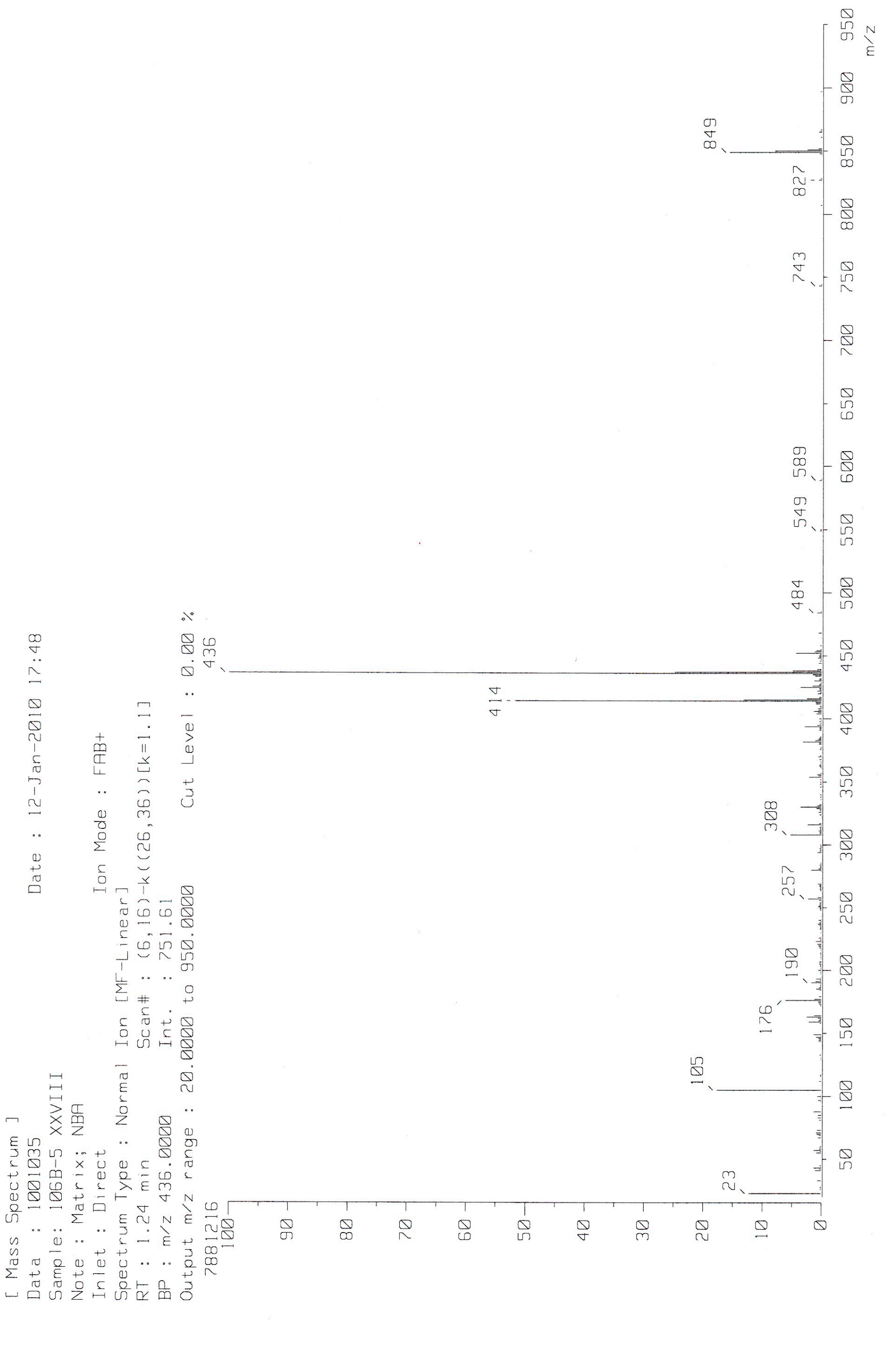

Figure S43 H and ^{13}C NMR spectra of **7** in $CDCl_3$

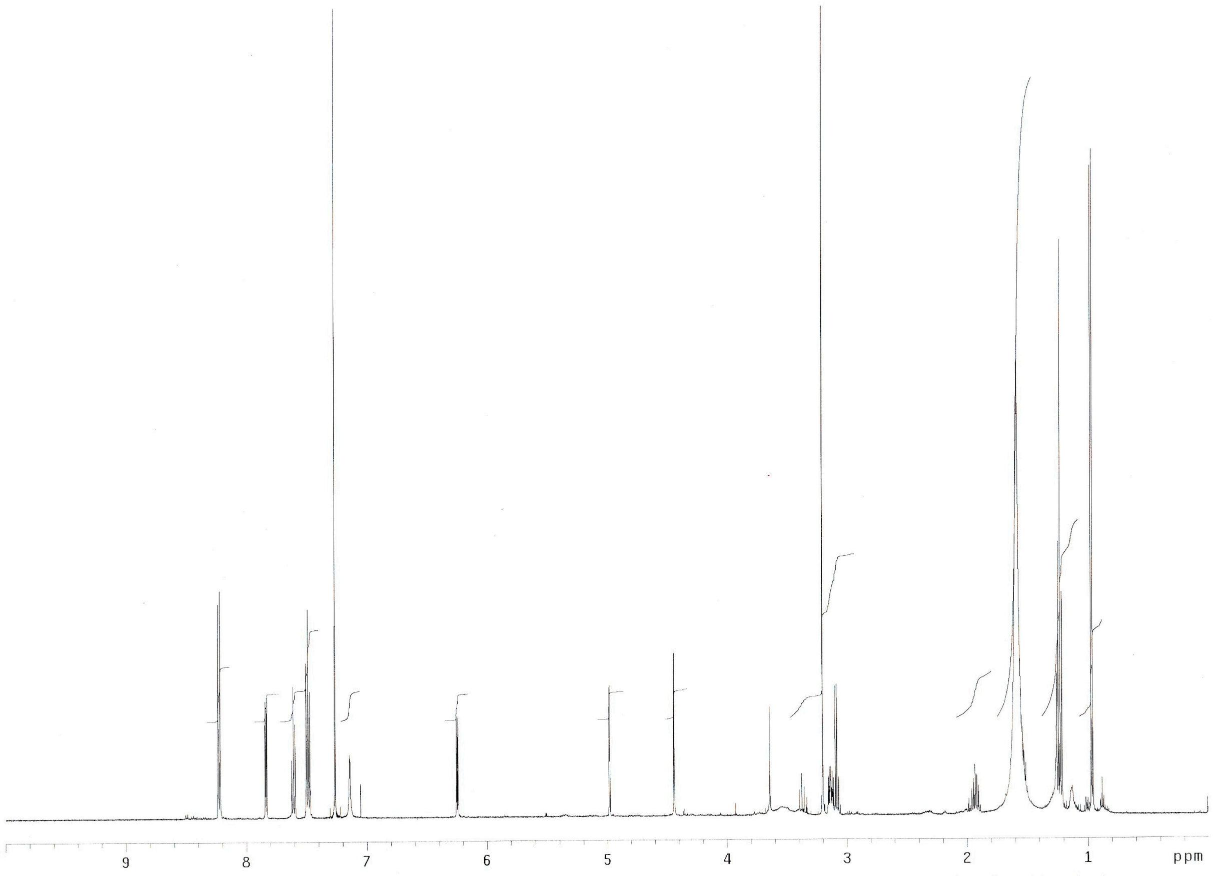

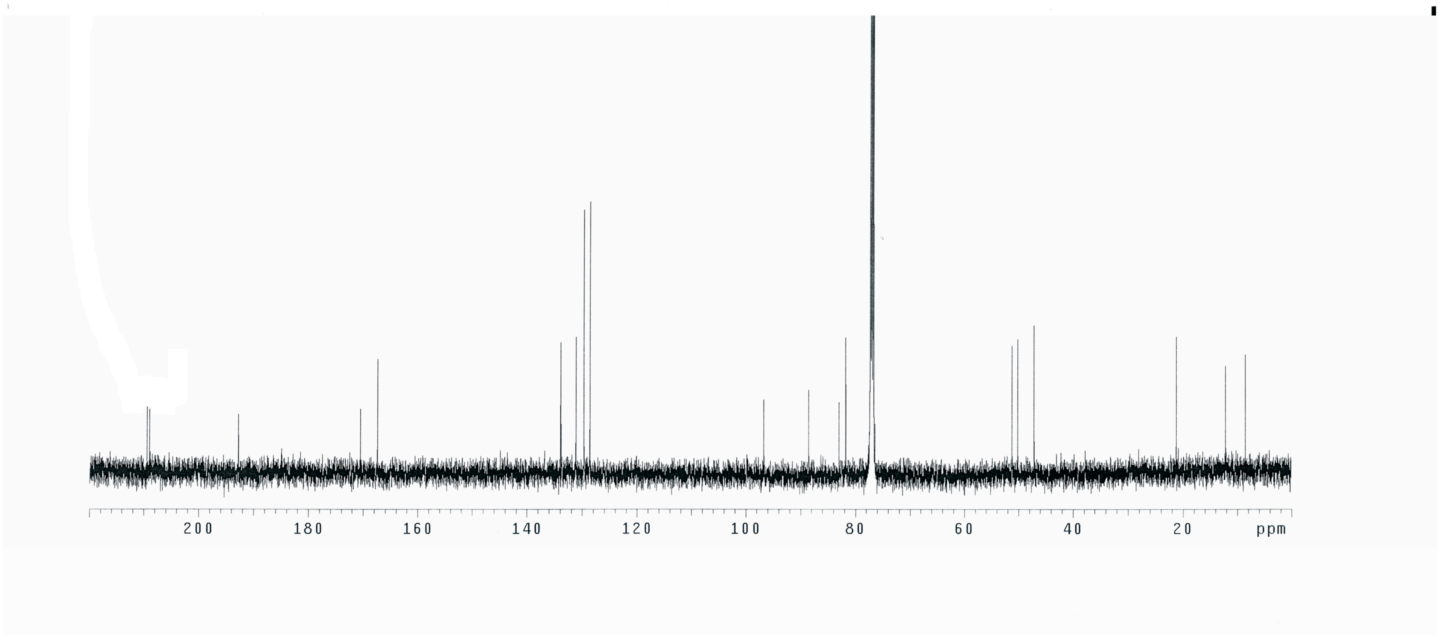

Figure S44 ^1H-^1H COSY of **7**

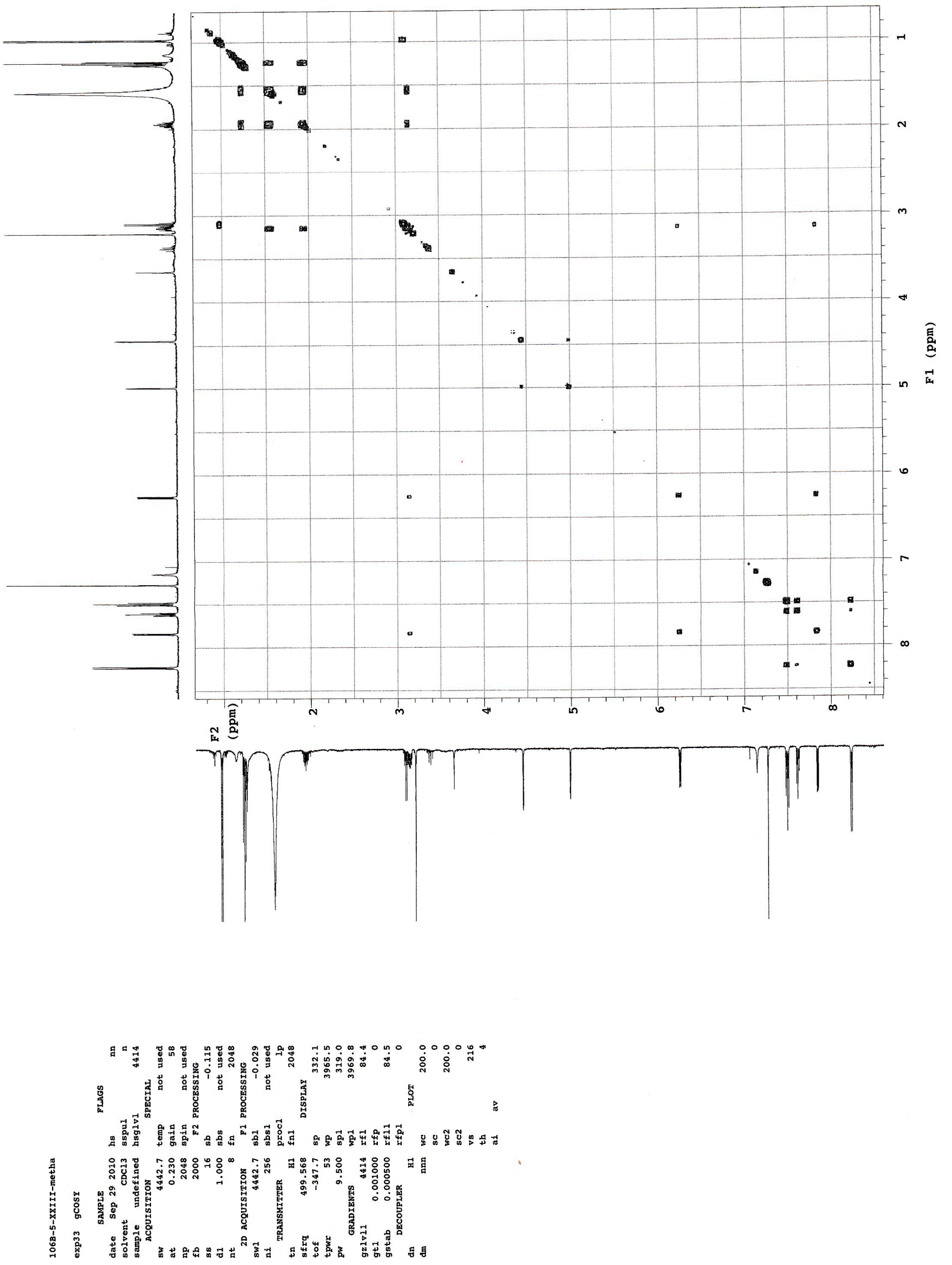

Figure S45 NOESY of **7**

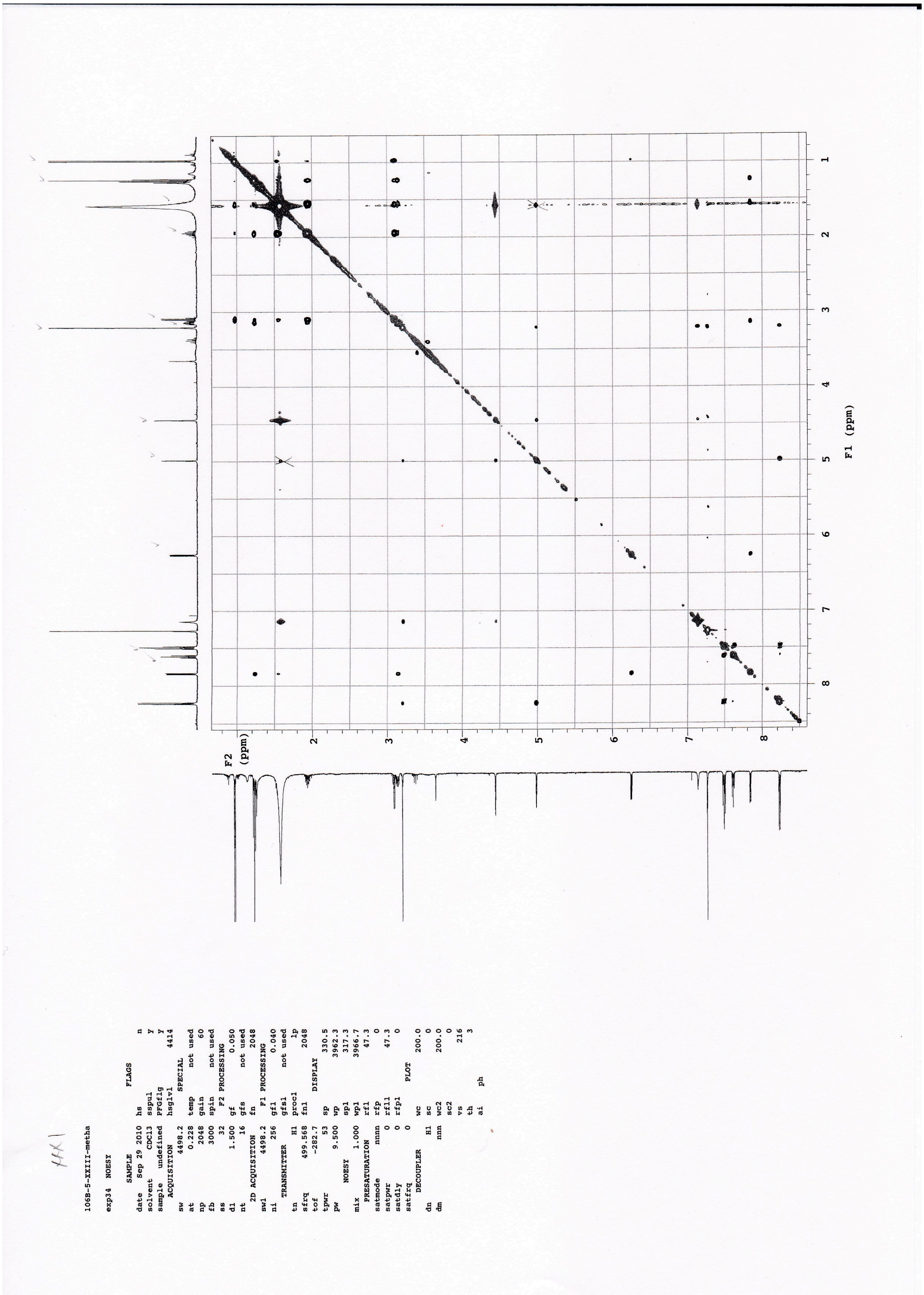

Figure S46 HMQC of **7**

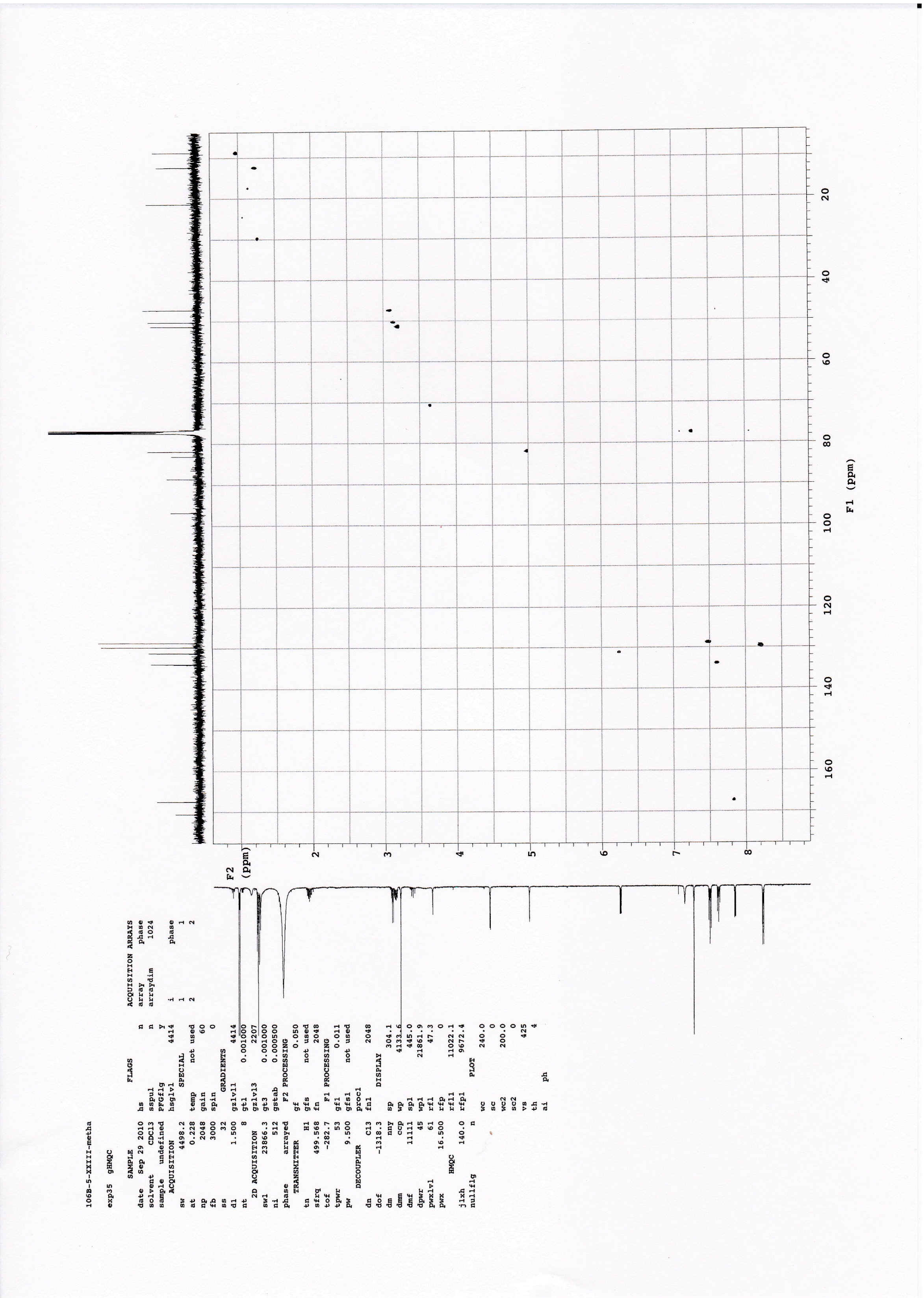

Figure S47 HMBC of **7**

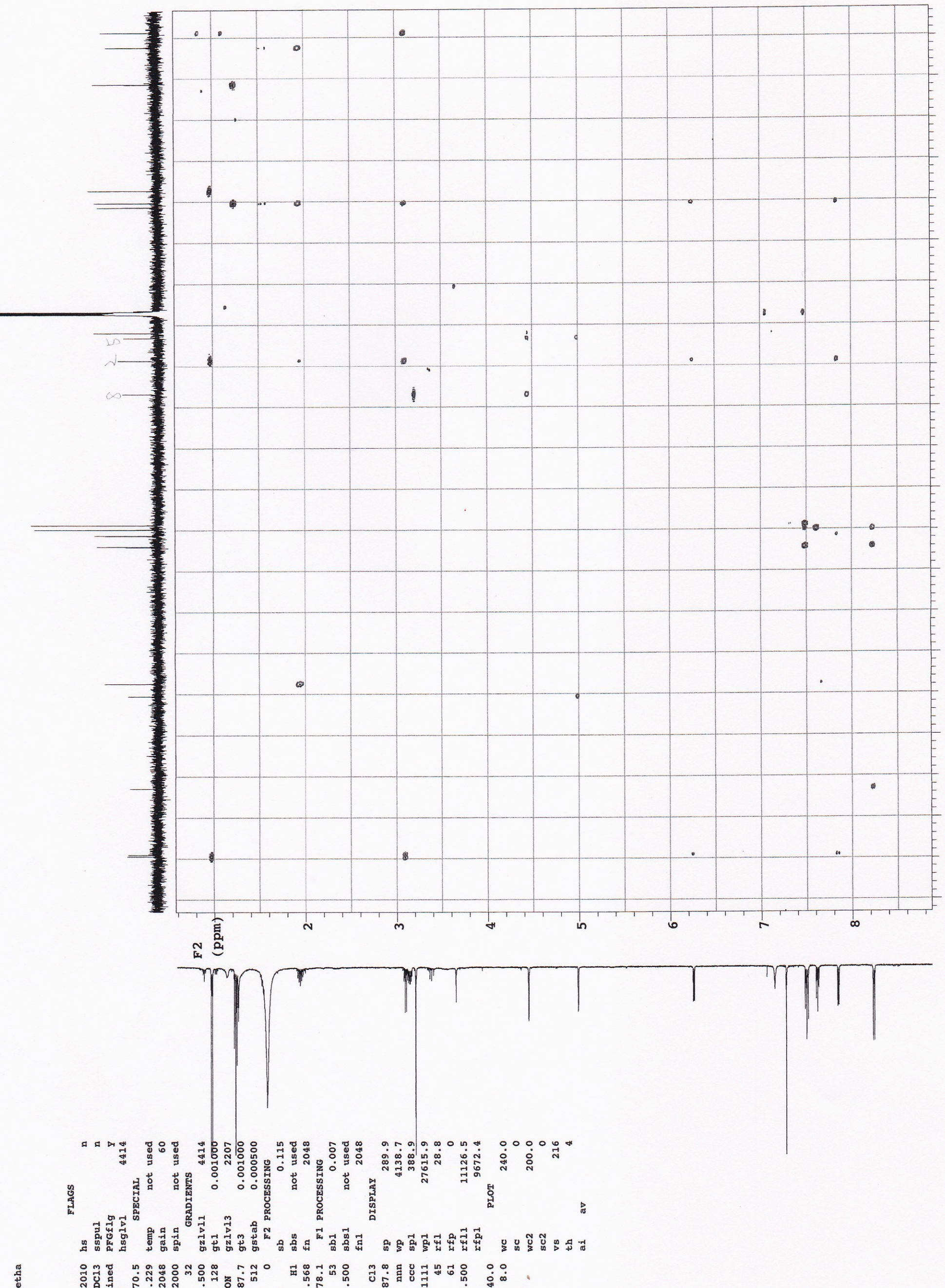

Figure S48 IR spectrum of **7**

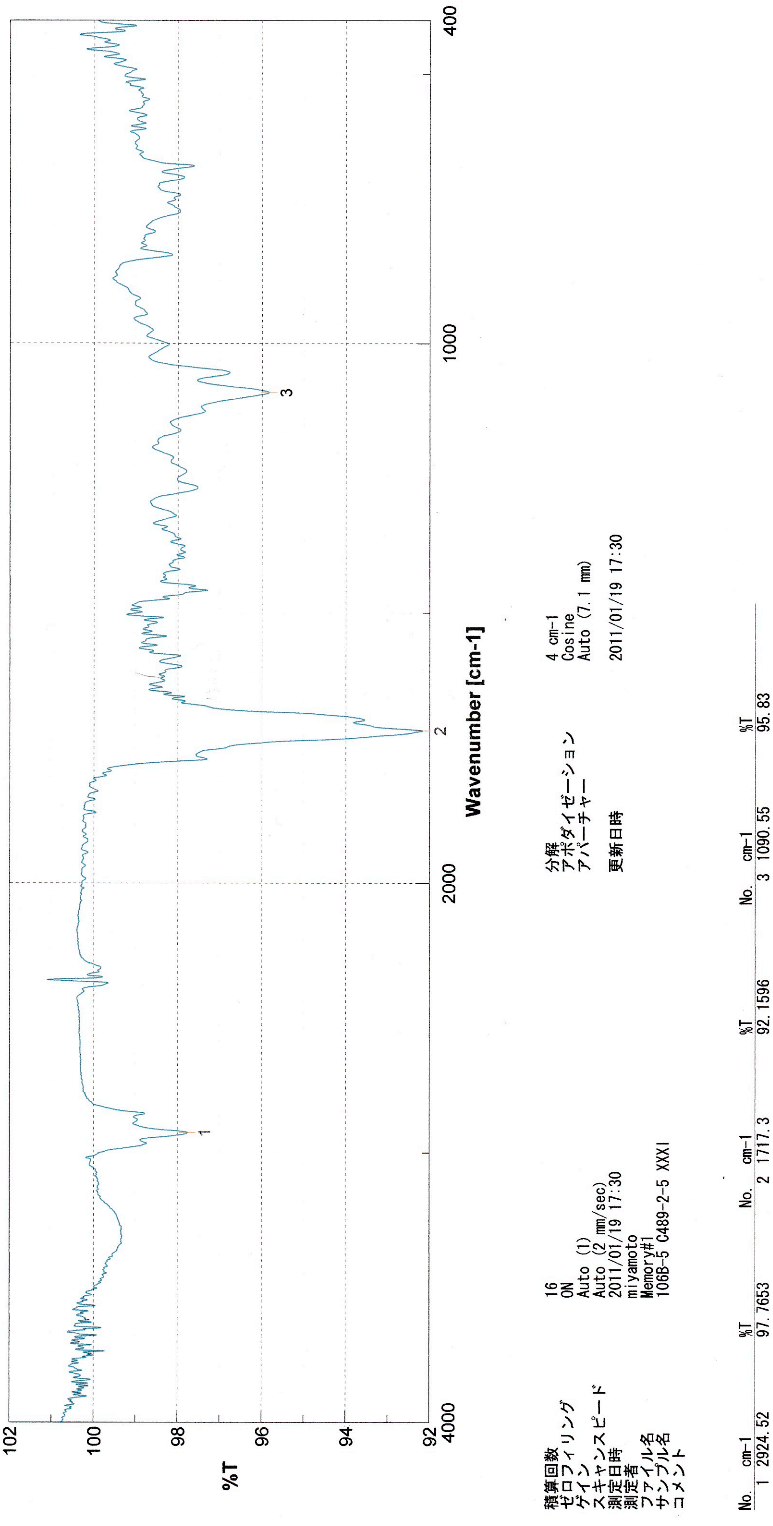

Figure S49 FABMS of **7**

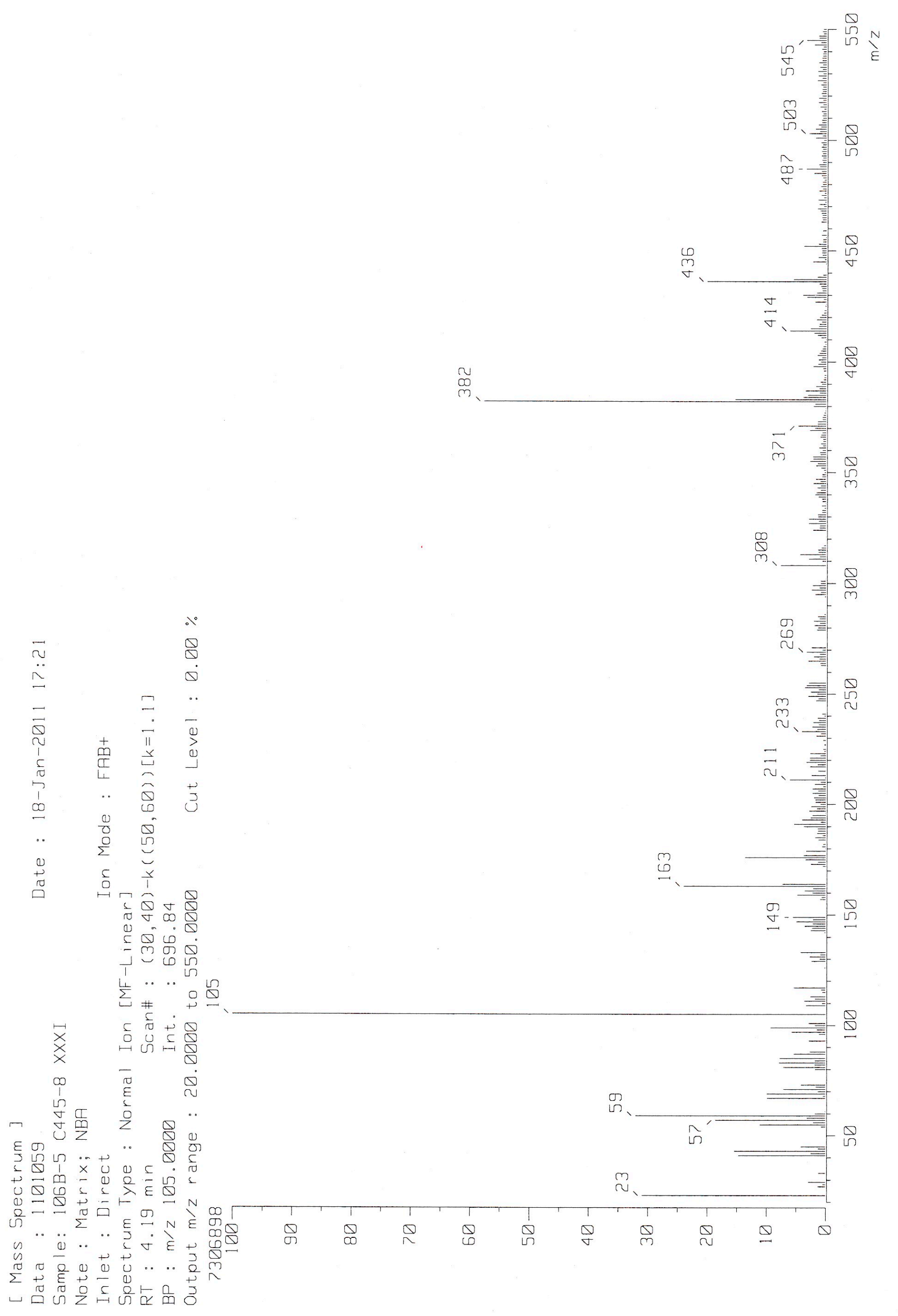

Figure S50^{1}H and ^{13}C NMR spectra of **8** in $CDCl_3$

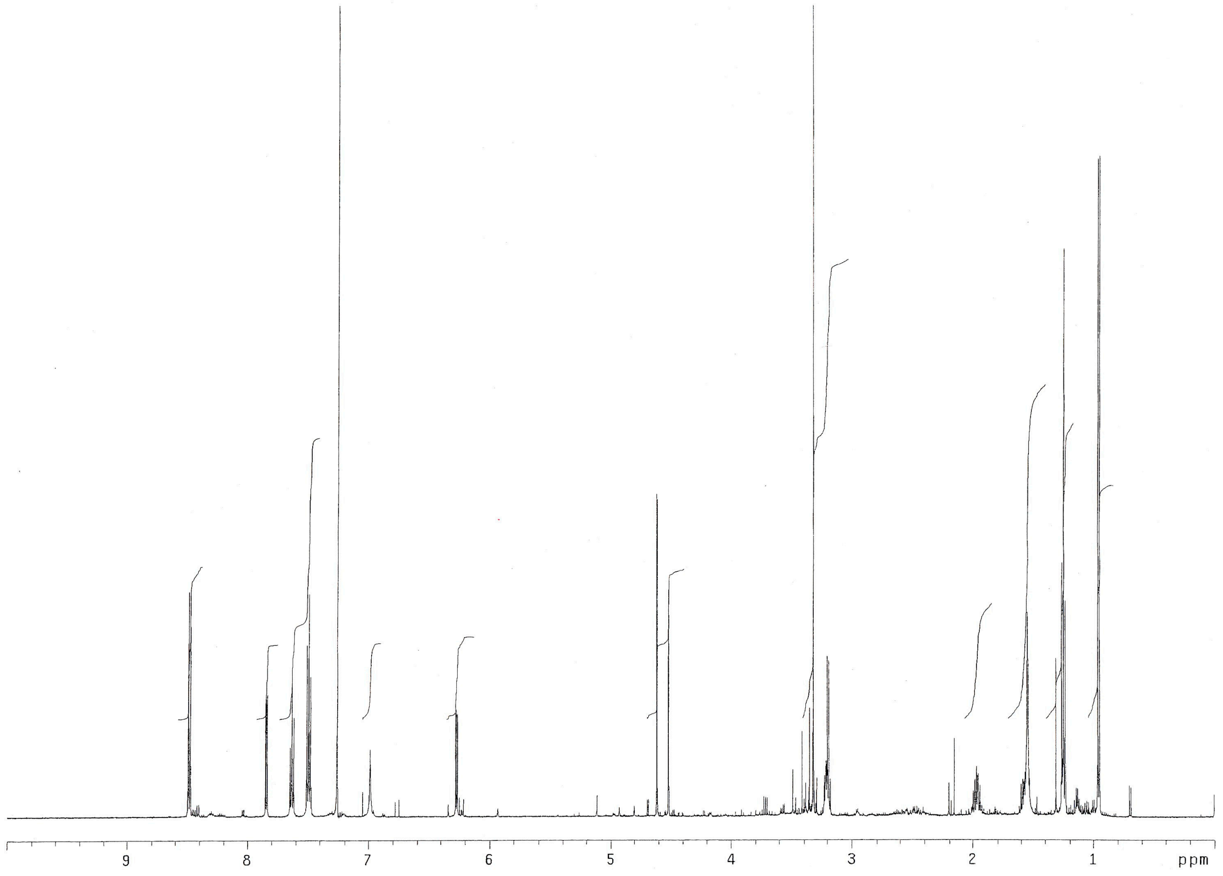

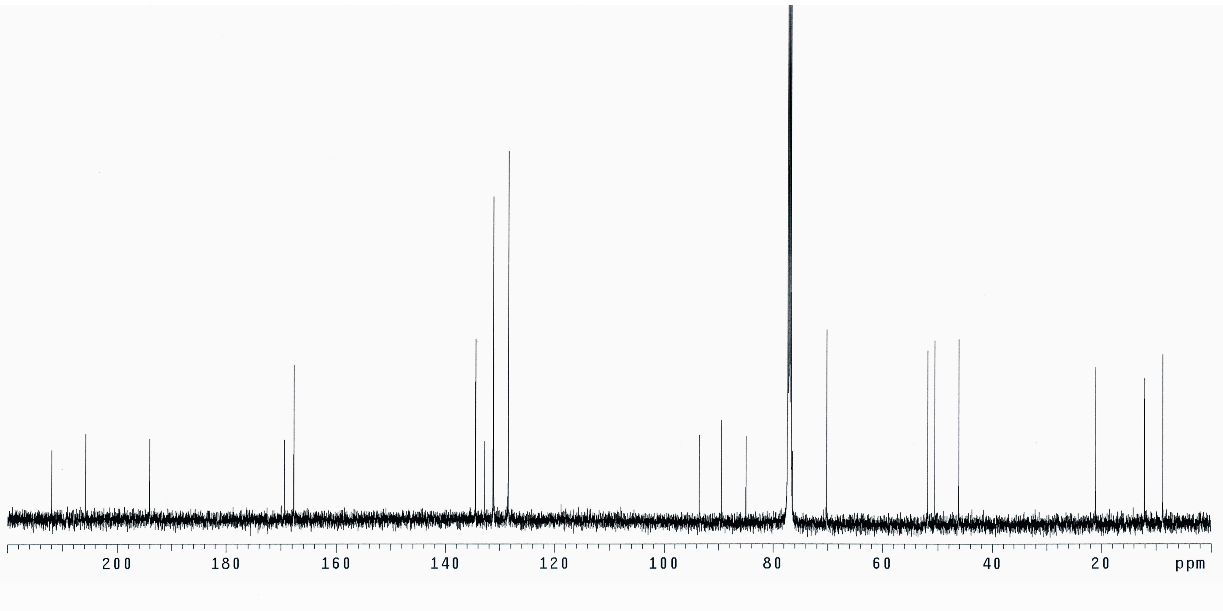

Figure S51 ¹H-¹H COSY of **8**

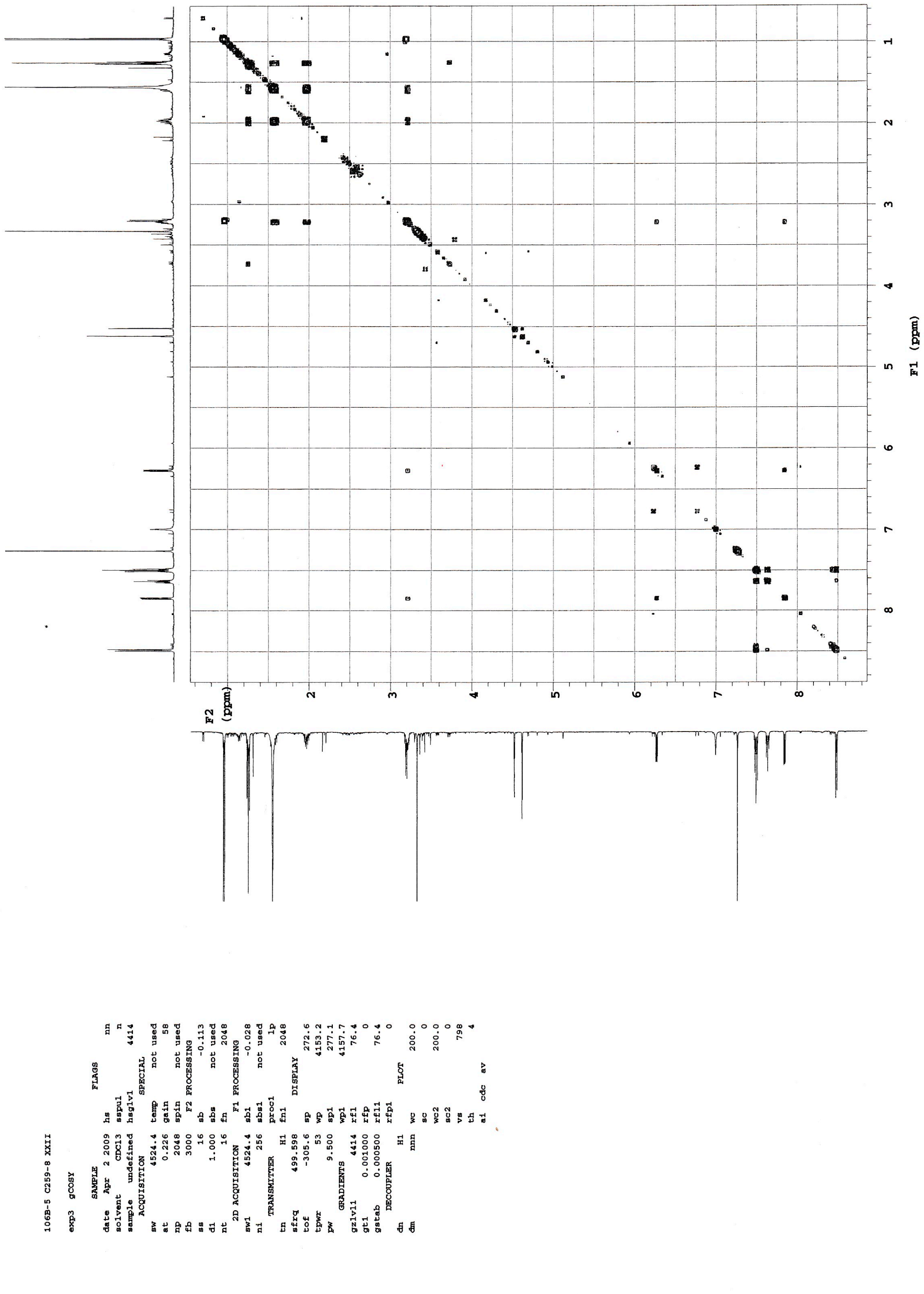

Figure S52 NOESY of **8**

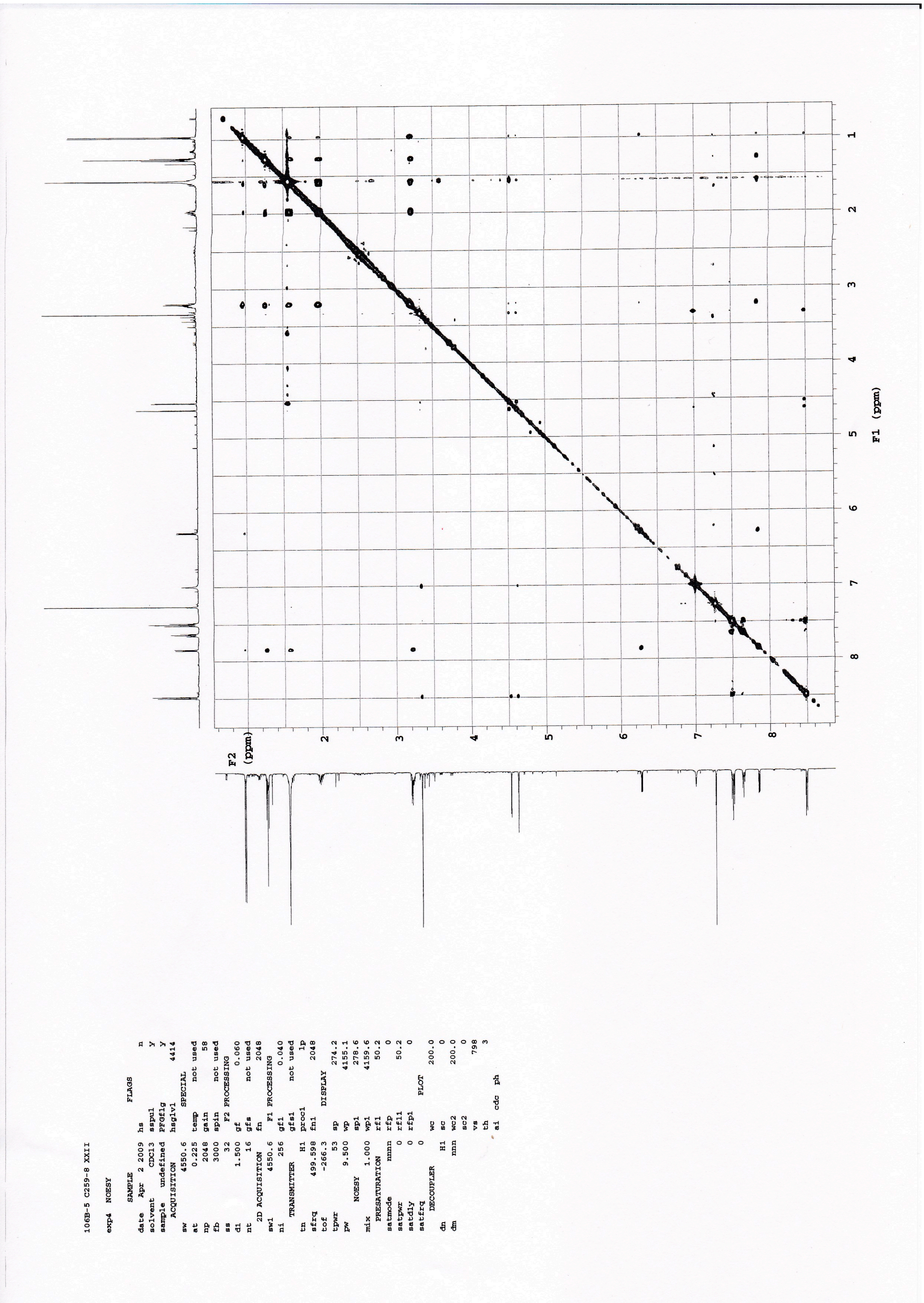

Figure S53 HMQC of **8**

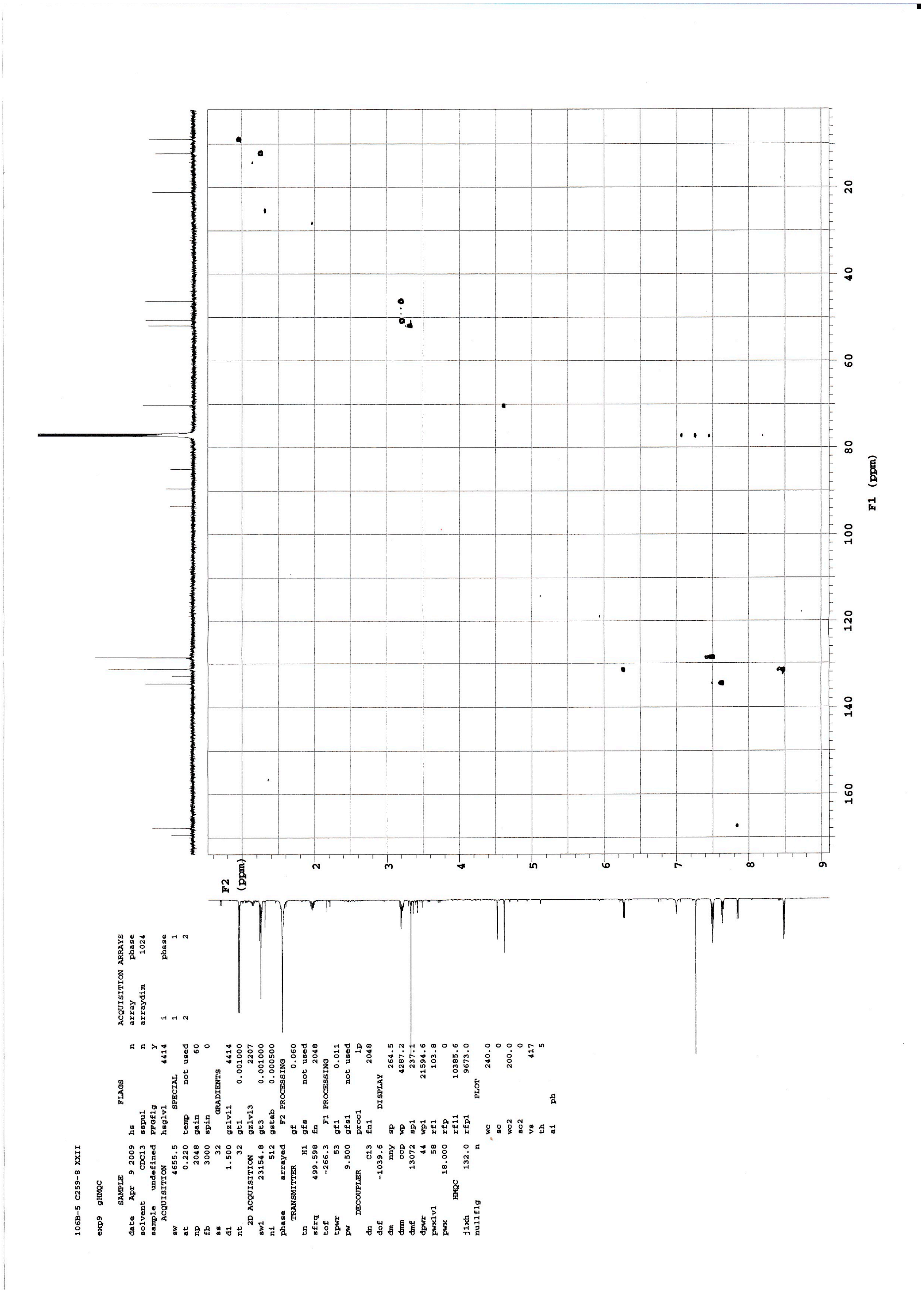

Figure S54 HMBC of **8**

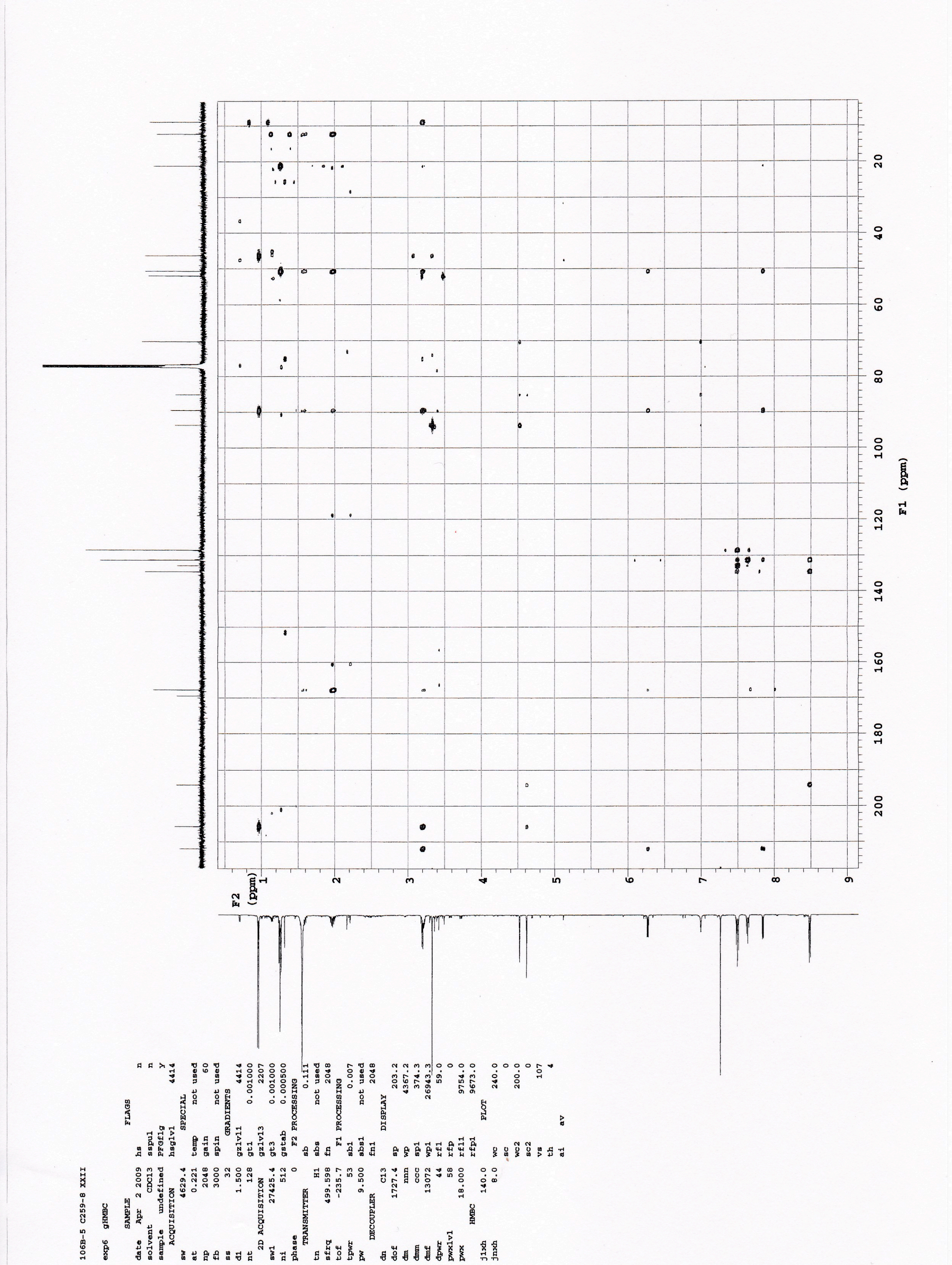

Figure S55 IR spectrum of **8**

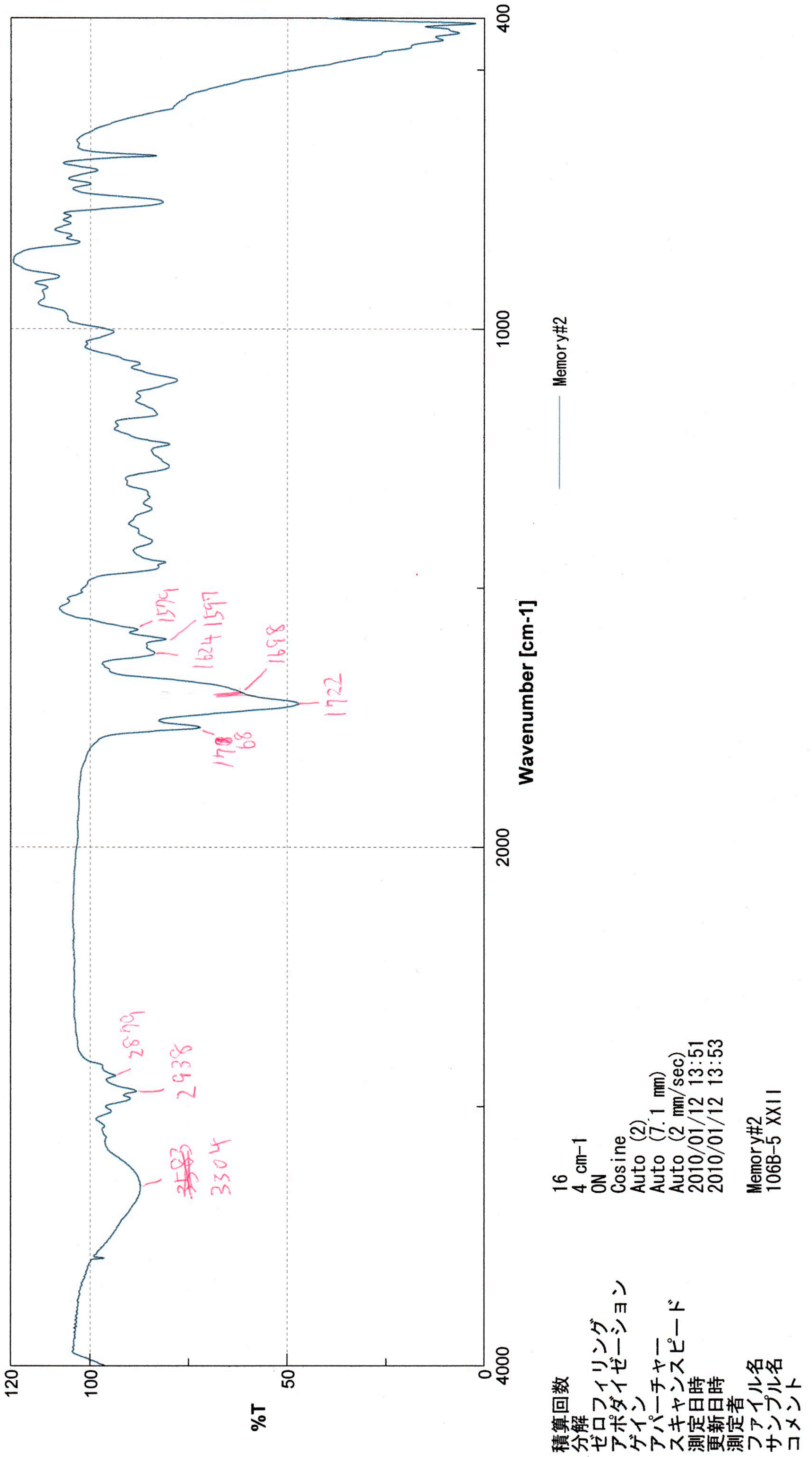

Figure S56 FABMS of **8**

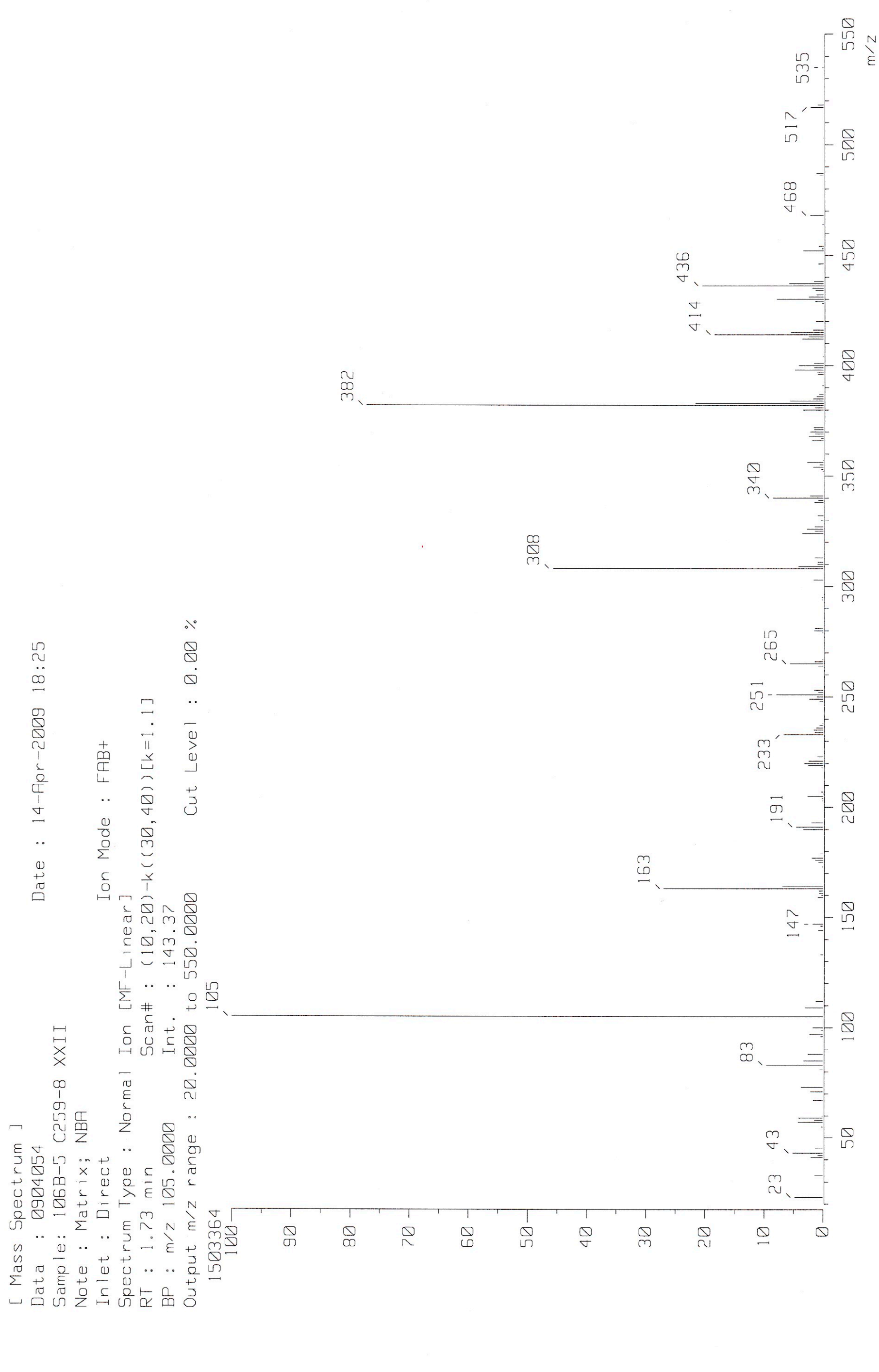

Figure S57 ^{1}H NMR spectrum of **1’**

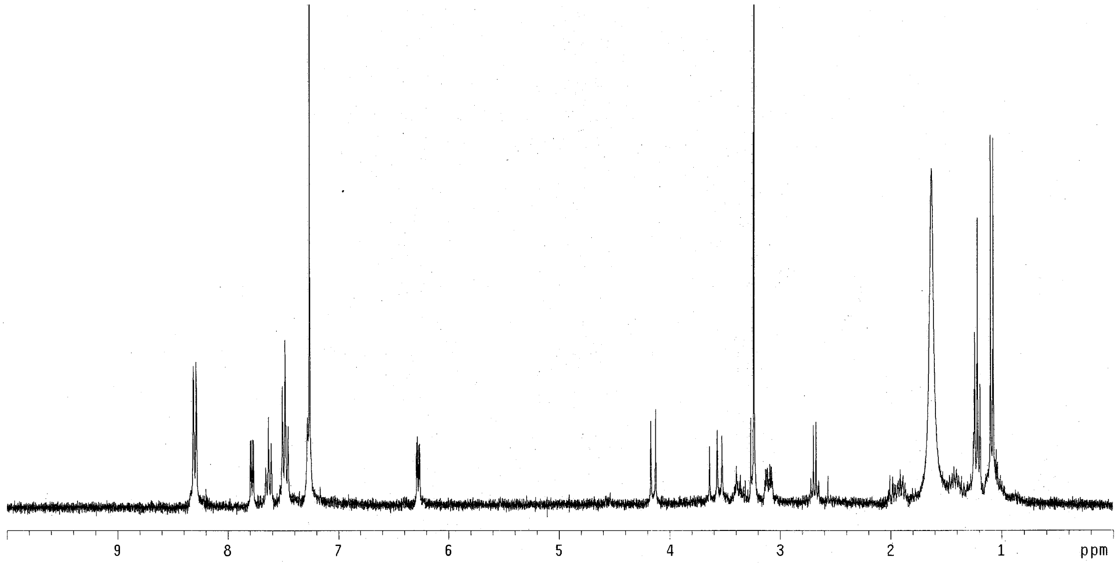

Figure S58 ^{1}H NMR spectrum of **2’**

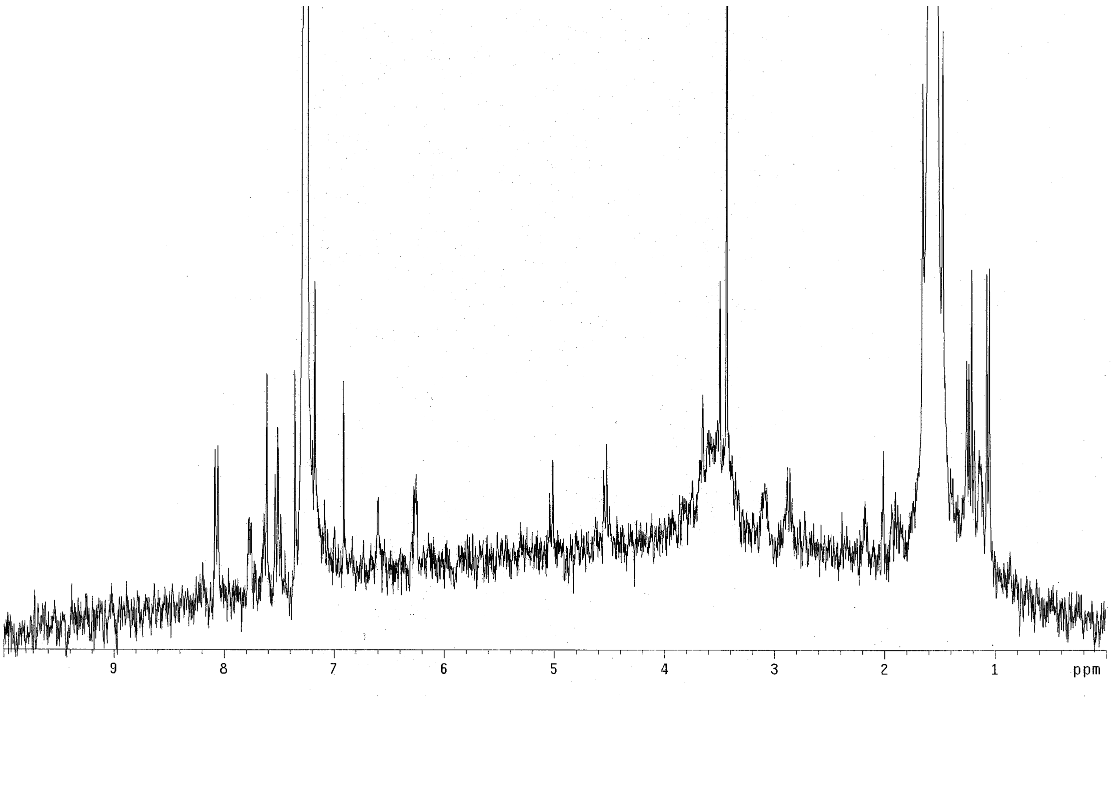

Figure S59 ^{1}H NMR spectrum of **3'**

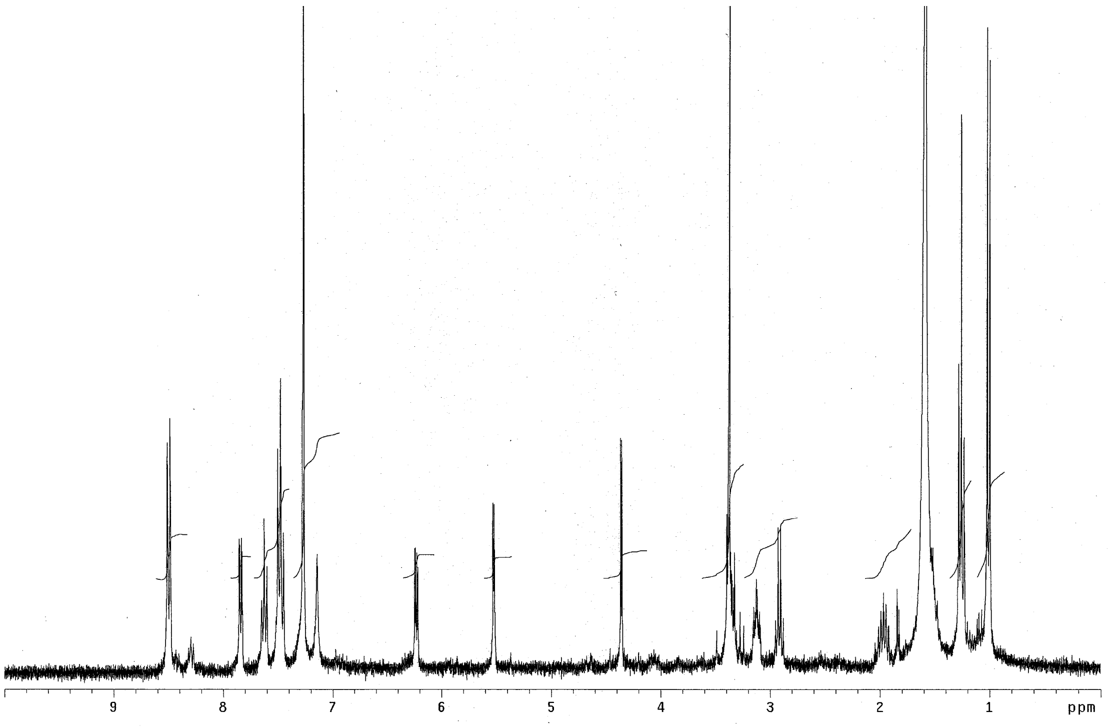

Figure S60 ^{1}H NMR spectrum of **4'**

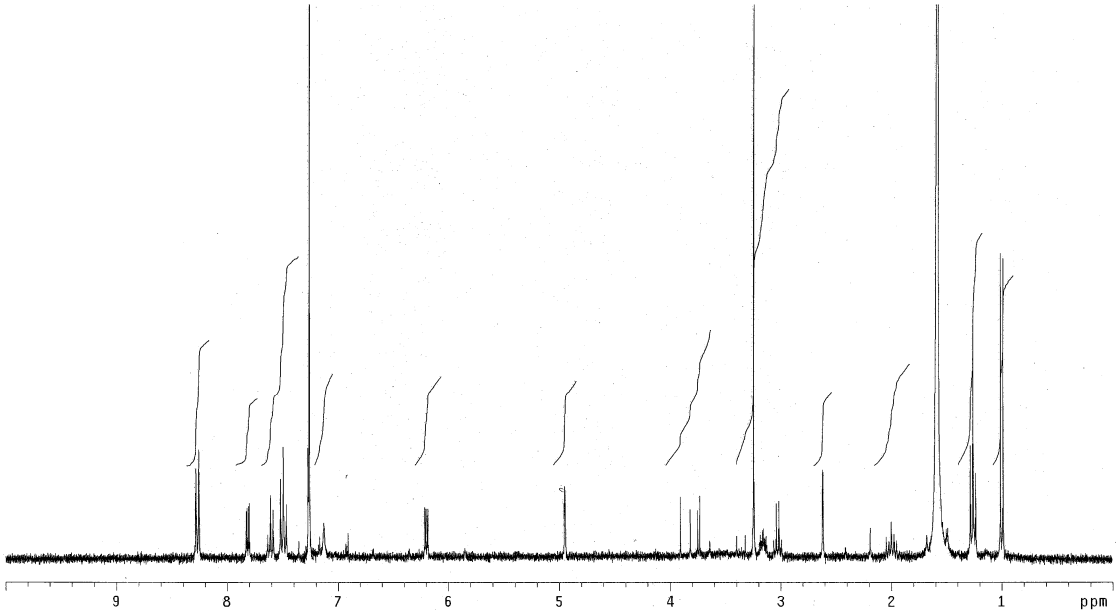

Figure S61 ^{1}H NMR spectrum of **5'**

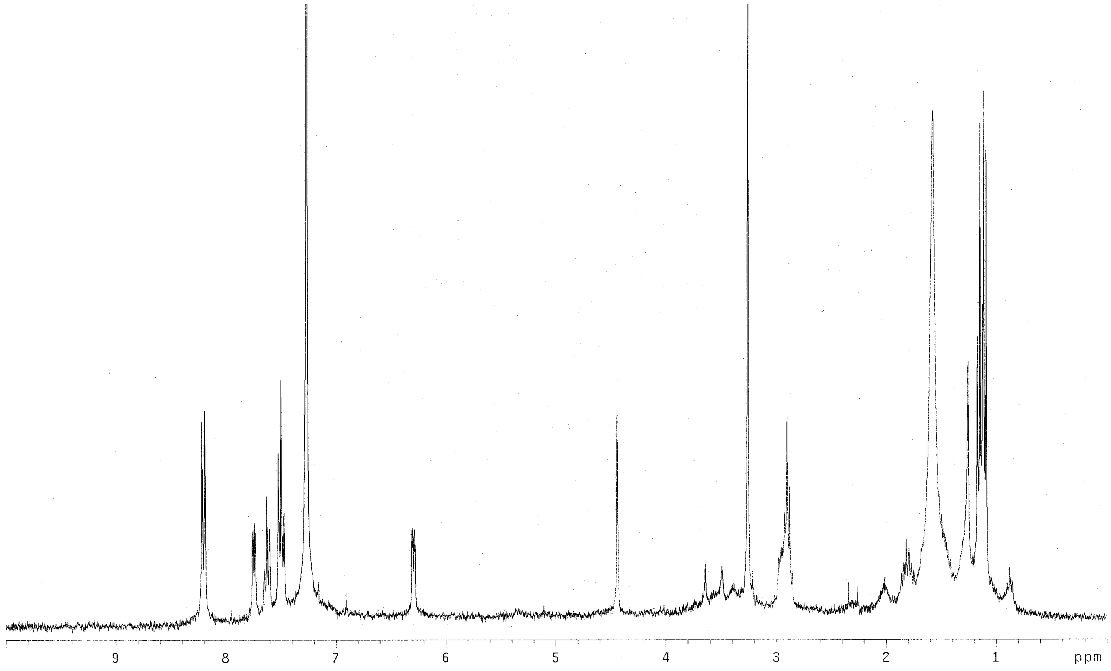

Figure S62 ^{1}H NMR spectrum of **6'**

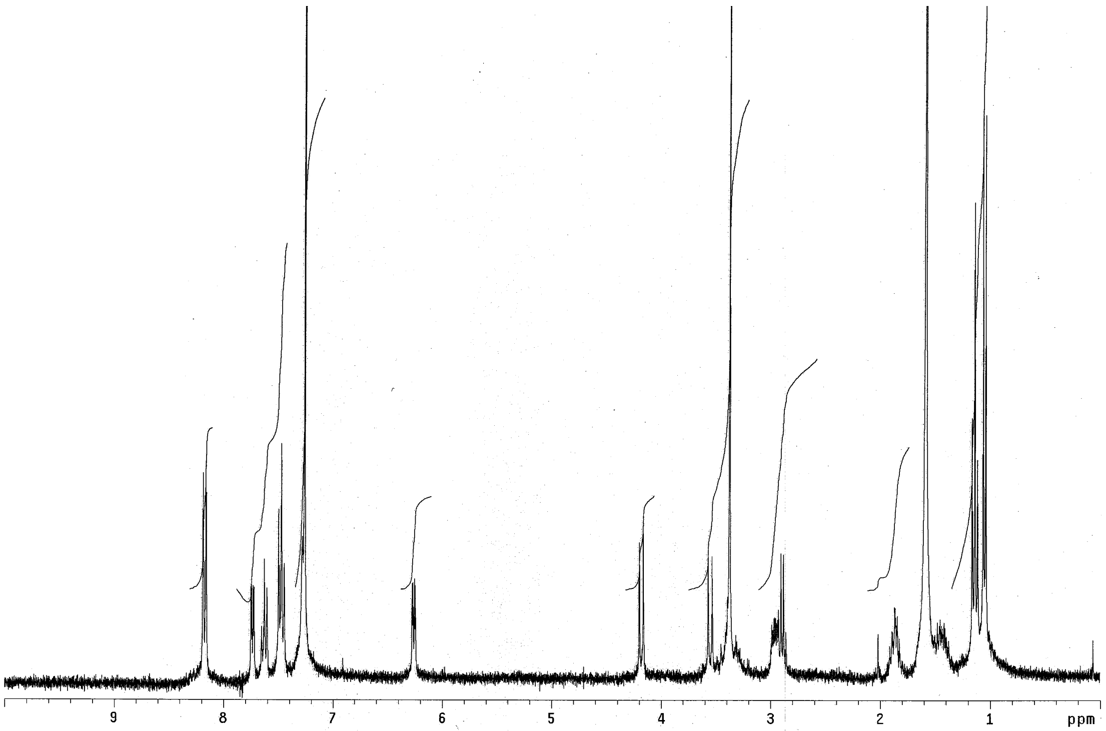

Figure S63 ^{1}H NMR spectrum of **7'**

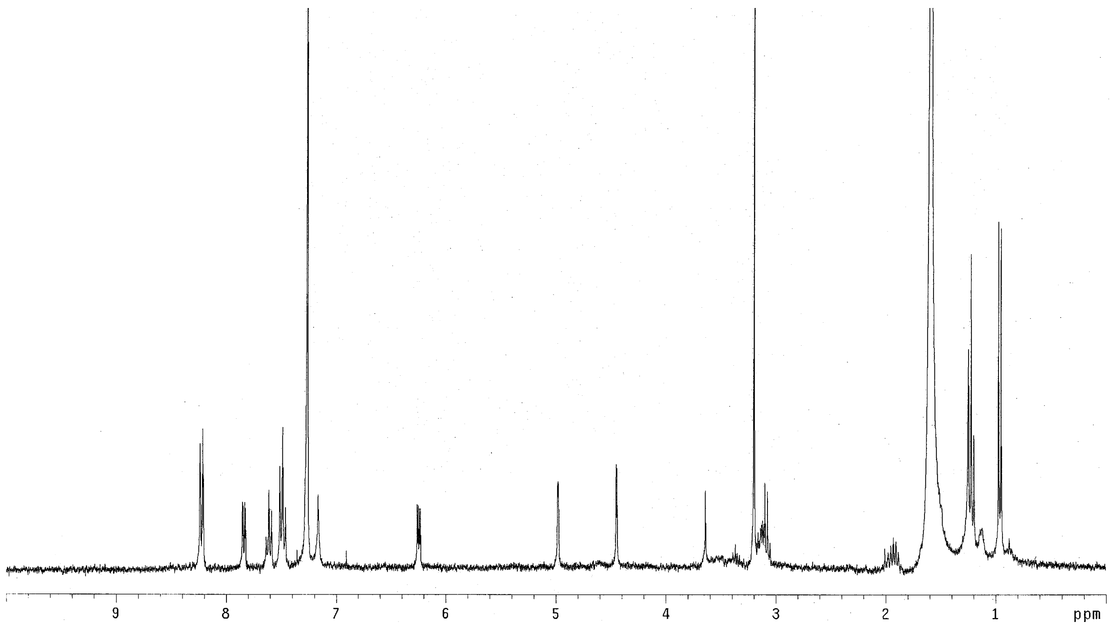

Figure S64 ^{1}H NMR spectrum of **8'**

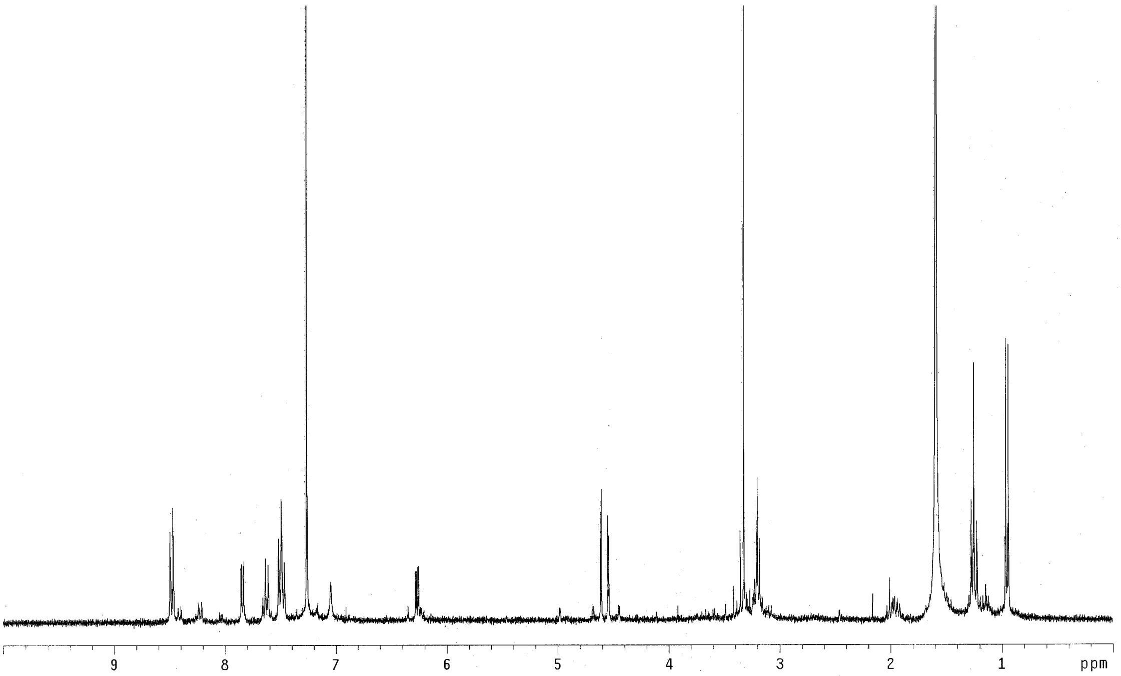

Figure S65

cephalimysin E (**1**)

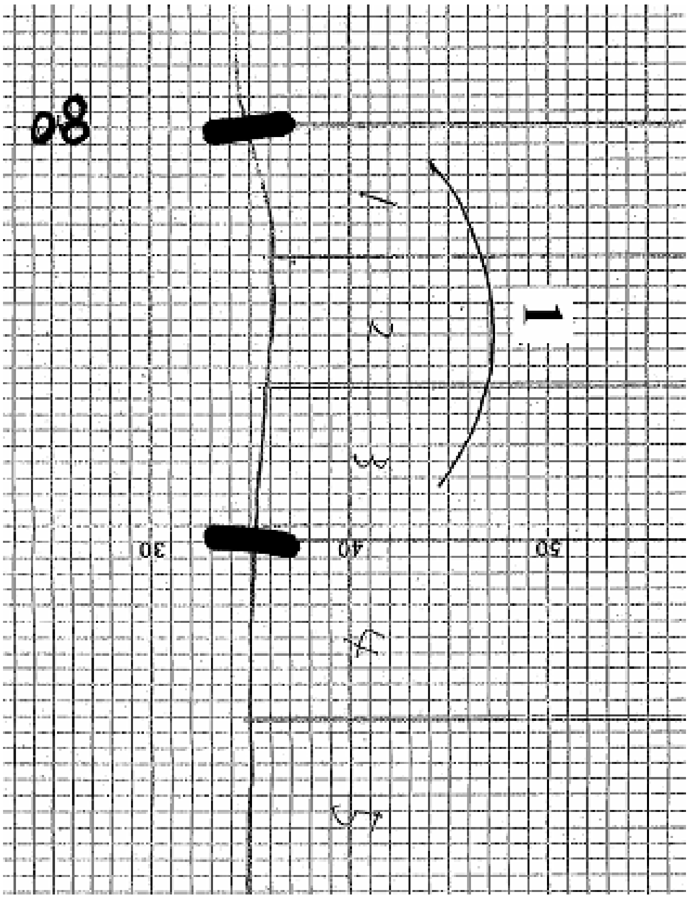

Moble phase CH_3CN / H_2O = 40 / 60, 4ml/min

Figure S66

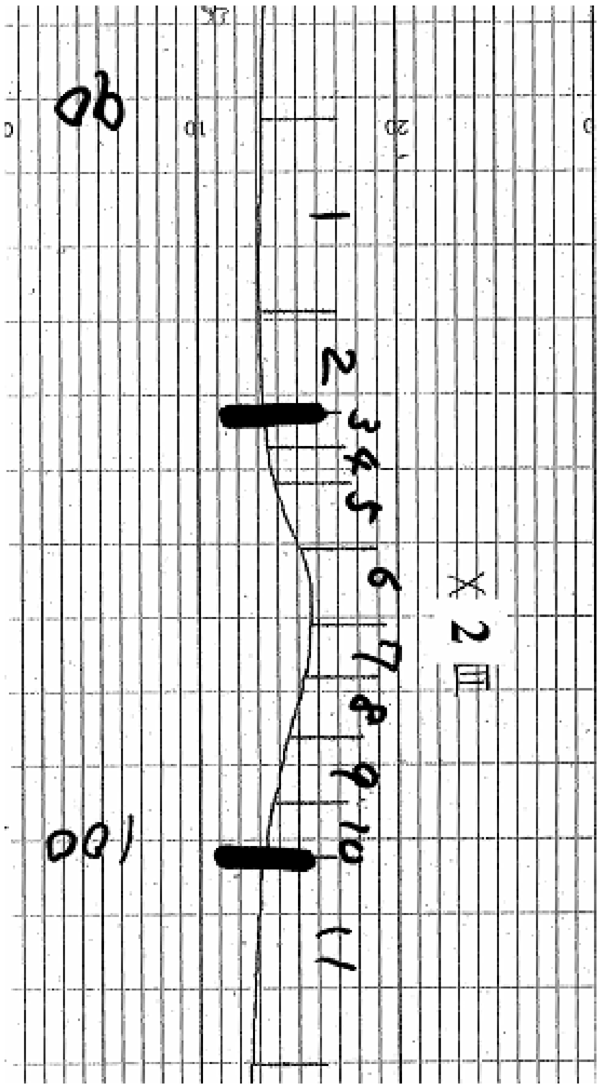

cephalimysin F (**2**)

Moble phase CH_3CN / H_2O = 30 / 70, 4ml/min

Figure S67

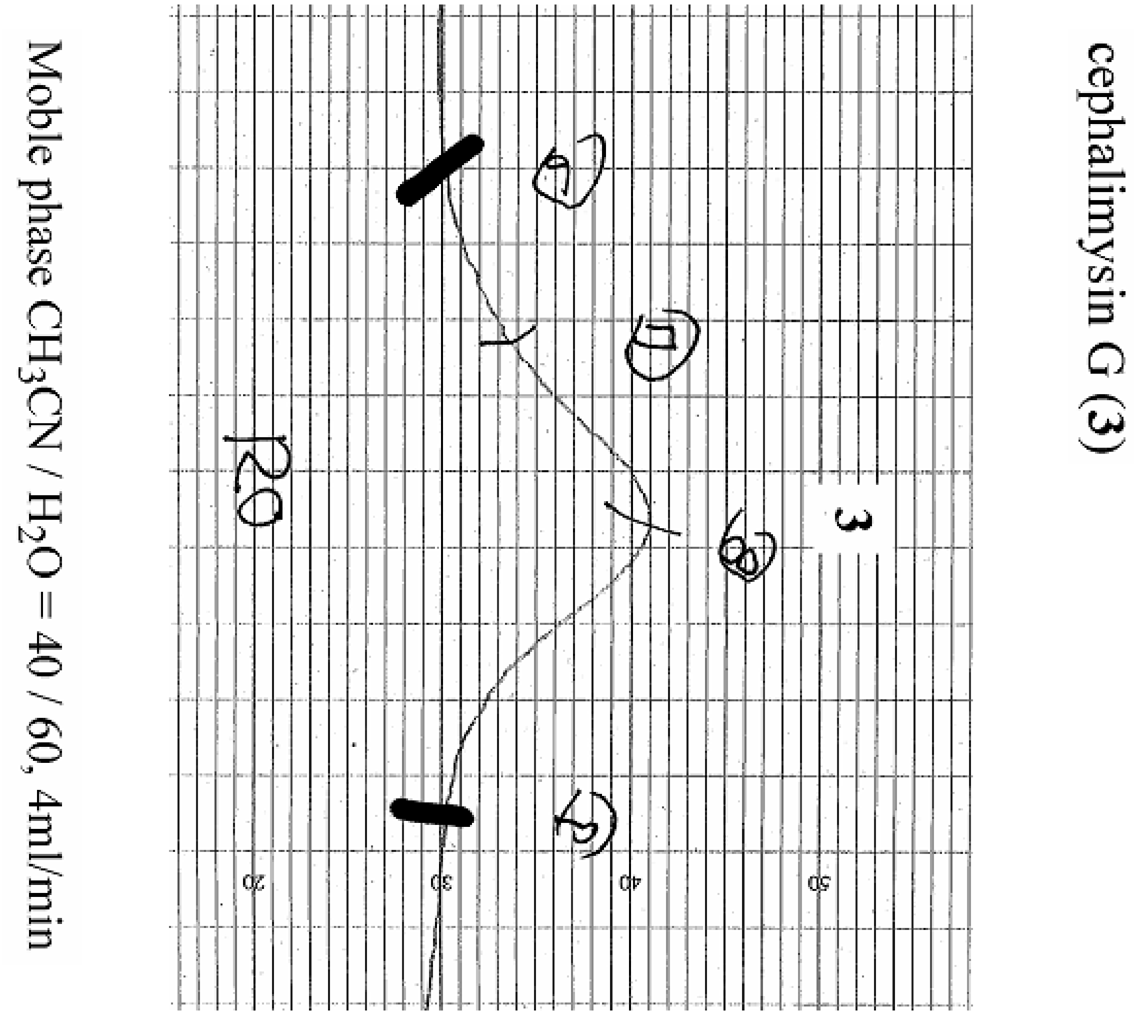
cephalimysin G (3)
3
20
30
40
50
Moble phase CH_3CN / H_2O = 40 / 60, 4ml/min

Figure S68

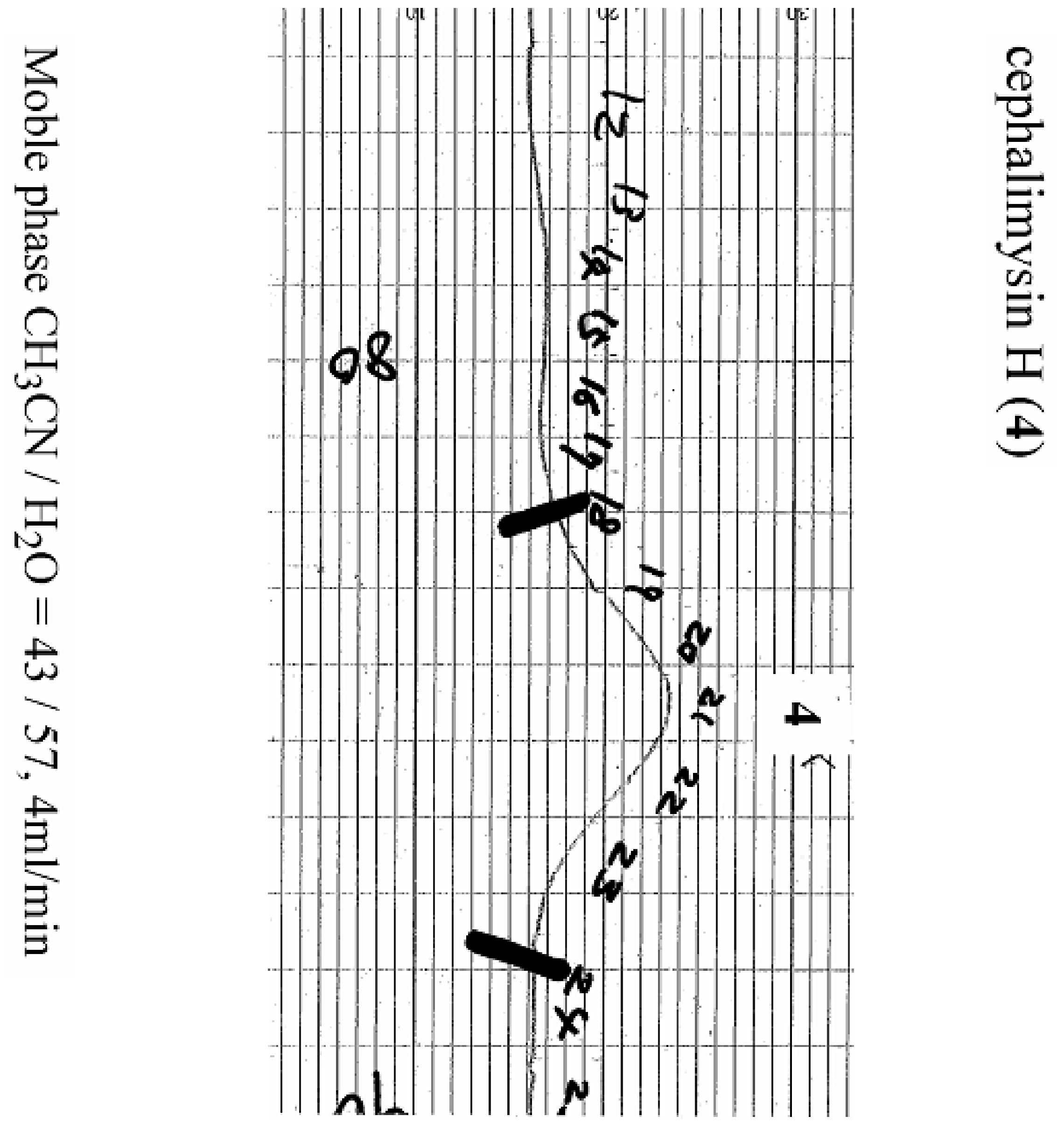

Figure S69

cephalimysin I (5)

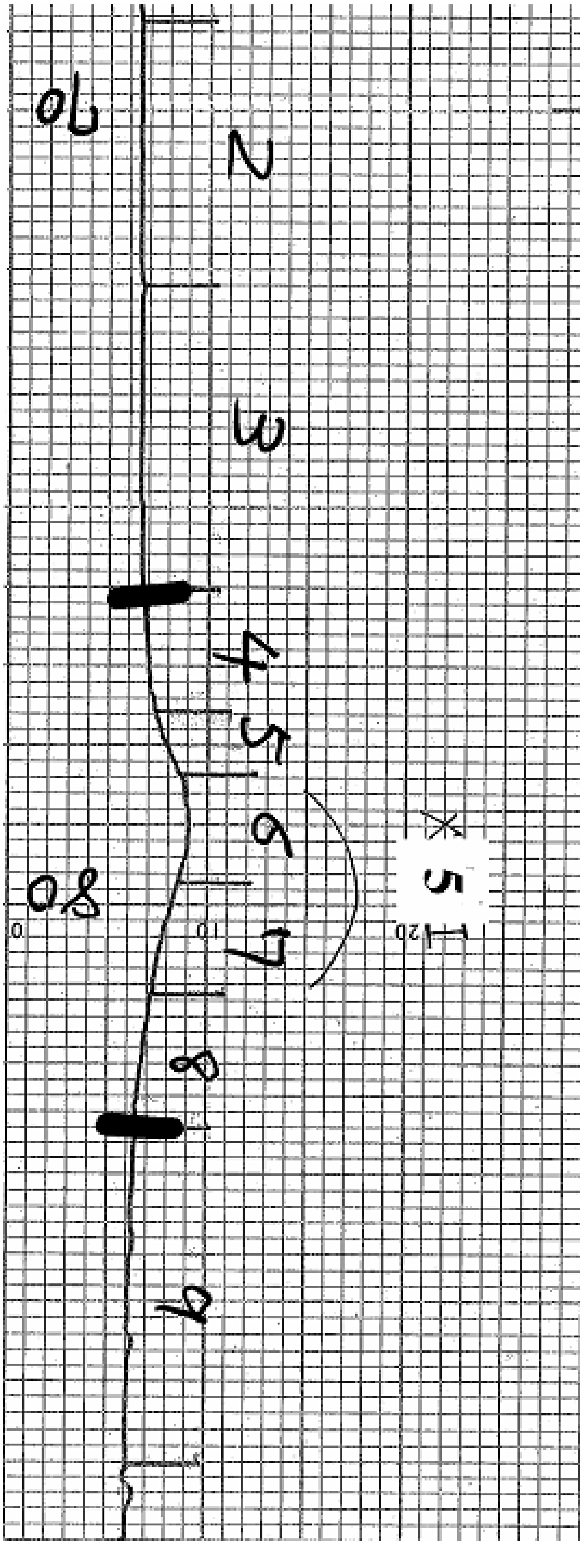

Moble phase CH_3CN / H_2O = 40 / 60, 4ml/min

Figure S70

cephalimysin J(6)

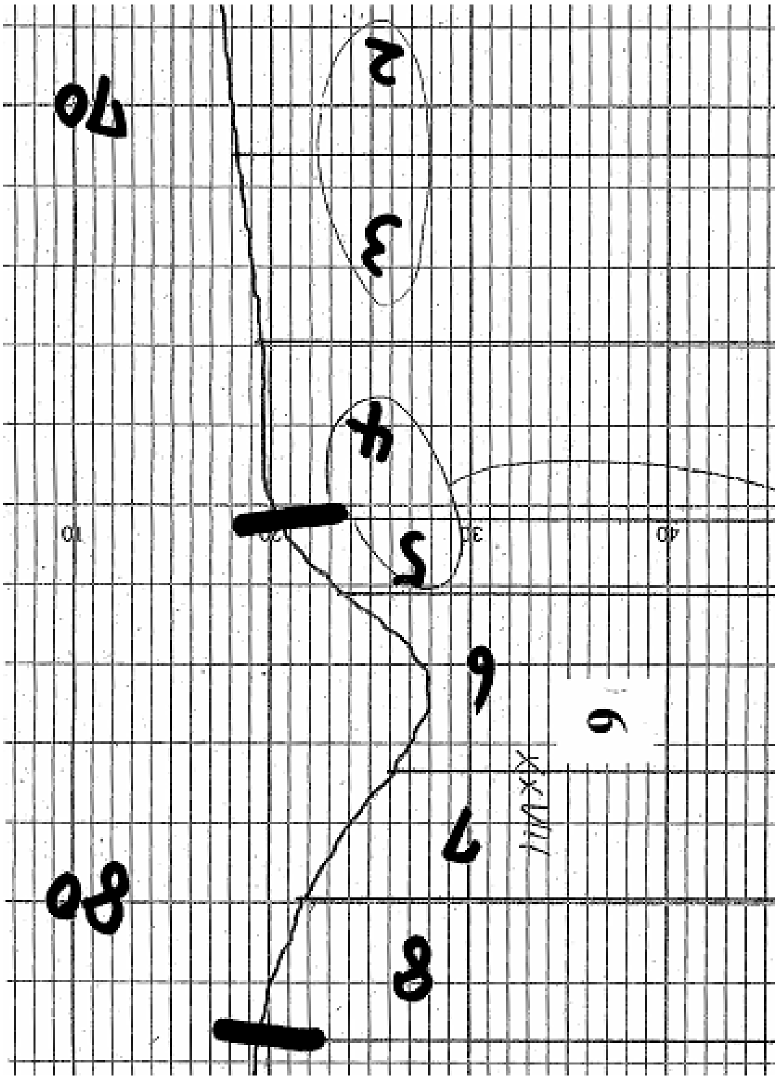

Moble phase CH_3CN / H_2O = 38 / 62, 4ml/min

Figure S71

cephalimysin K (7)

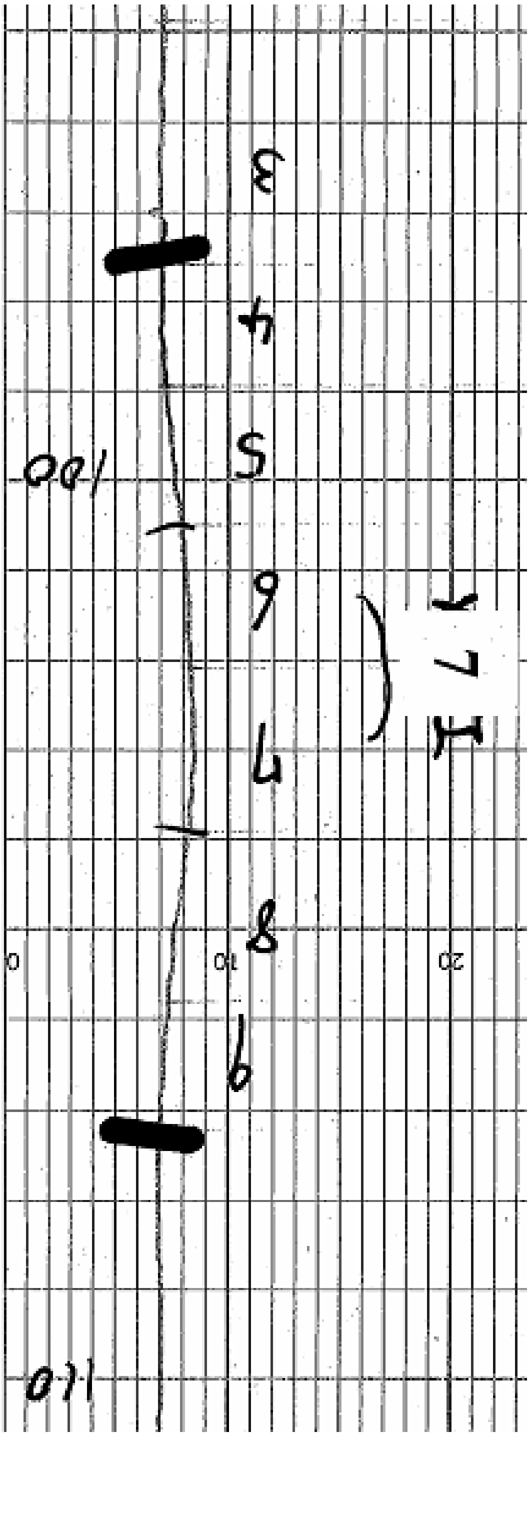

Moble phase CH_3CN / H_2O = 43 / 57, 4ml/min

Figure S72

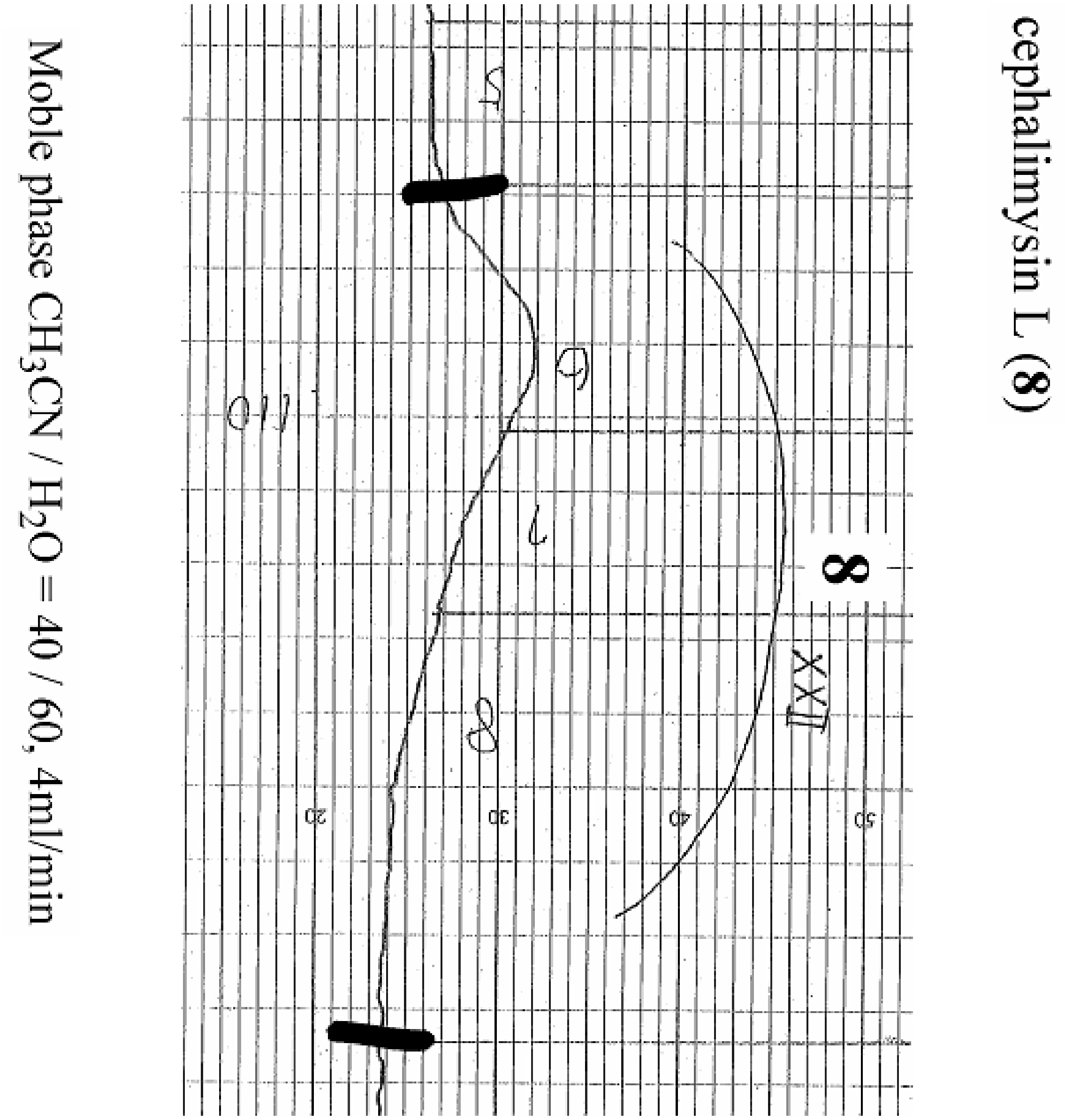
cephalimysin L (8)
8
20
30
40
50
Moble phase CH3CN / H2O = 40 / 60, 4ml/min

Figure S73

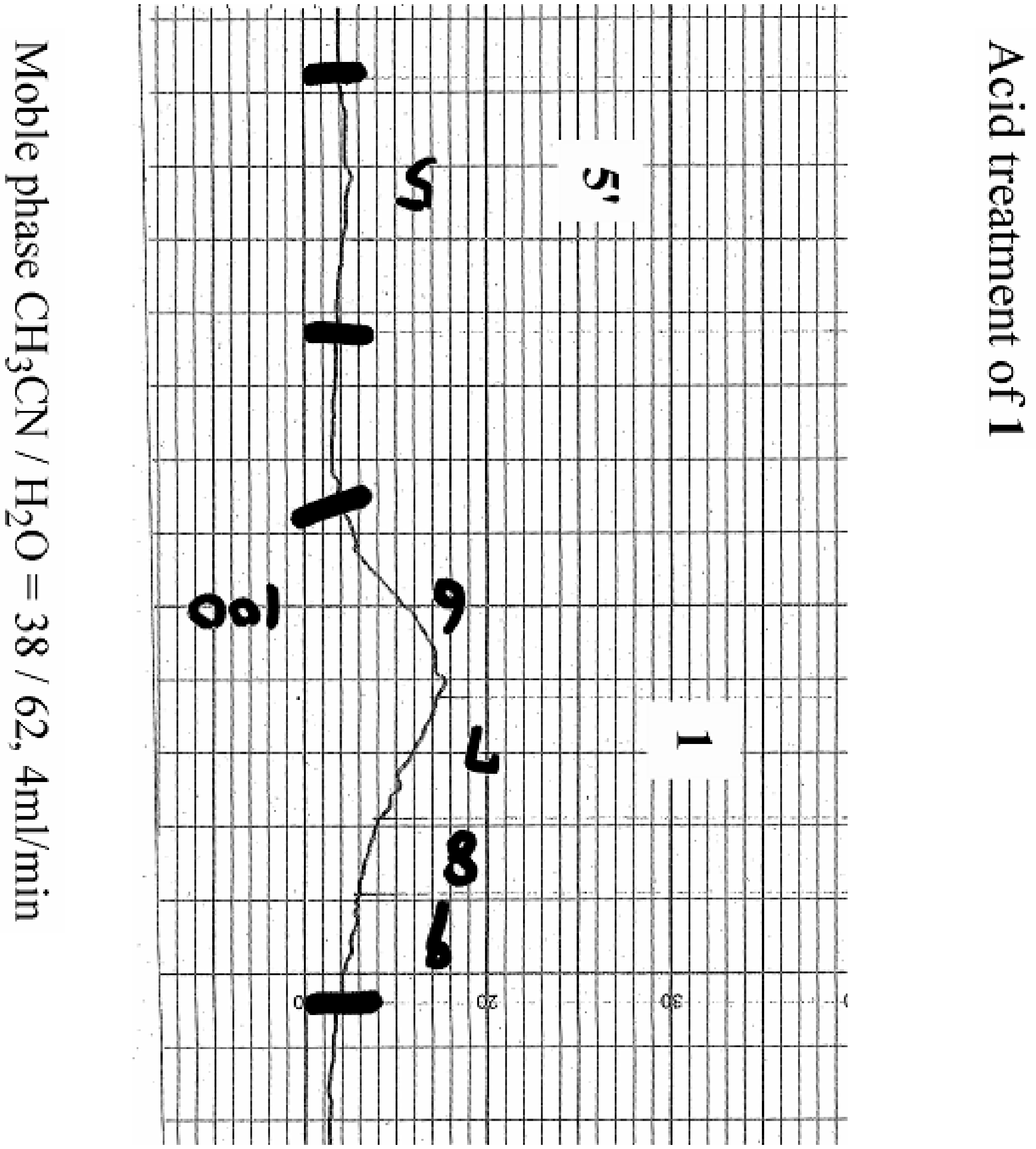

Figure S74

Acid treatment of **2**

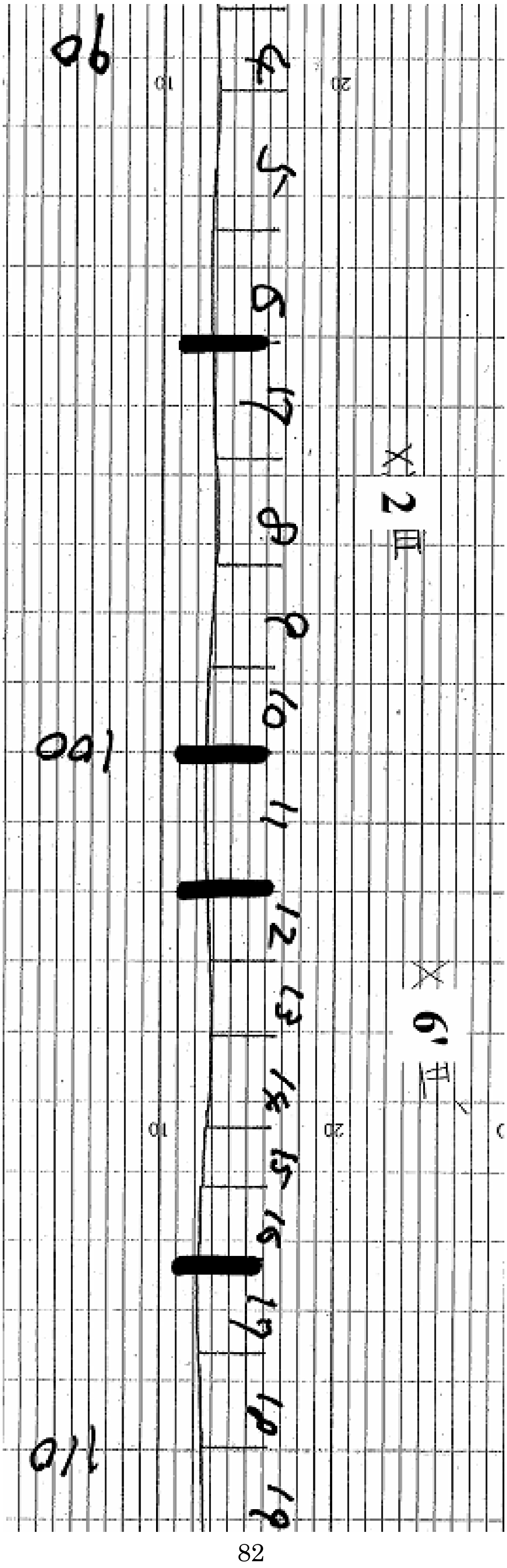

Moble phase CH_3CN / H_2O = 35 / 65, 4ml/min

Figure S75

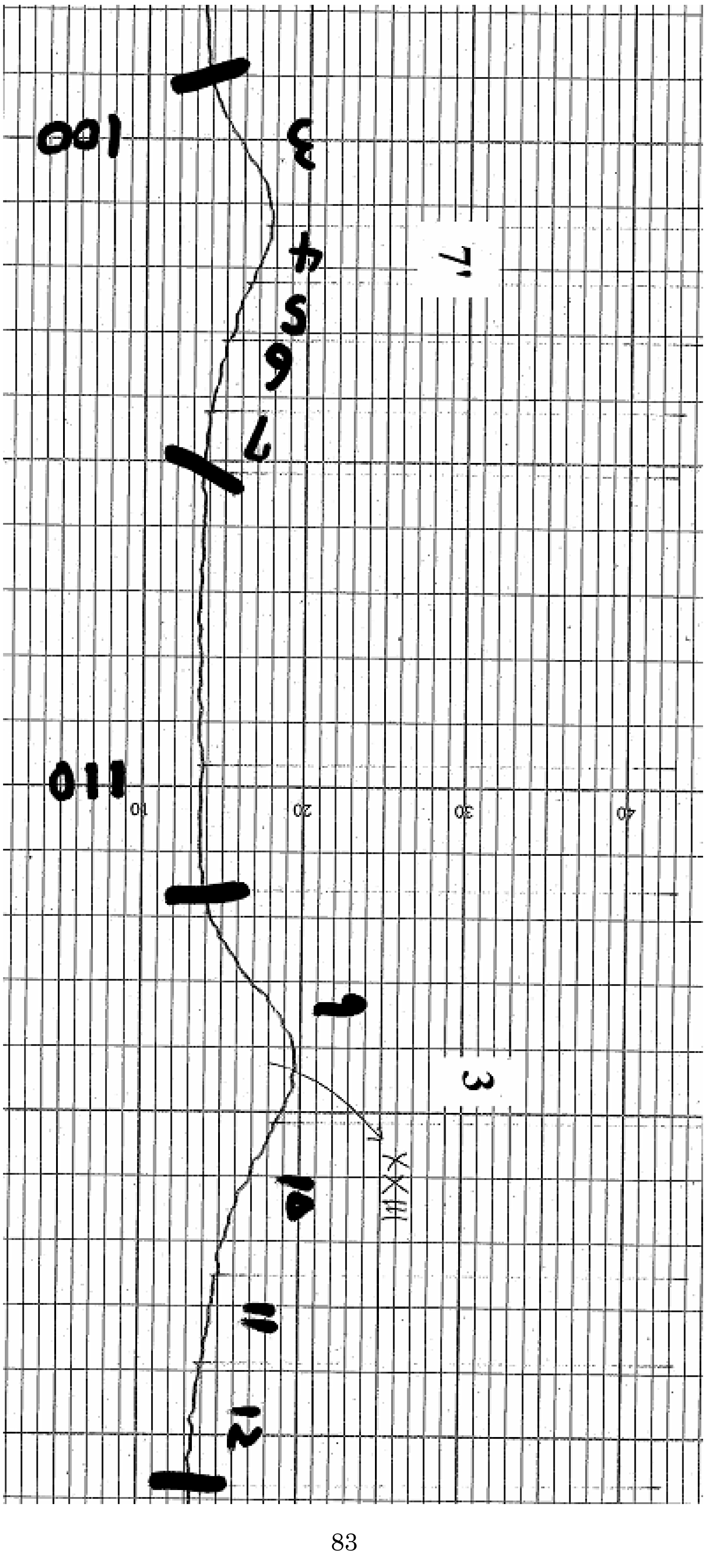

Acid treatment of 3

Moble phase CH_3CN / H_2O = 43 / 57, 4ml/min

Figure S76

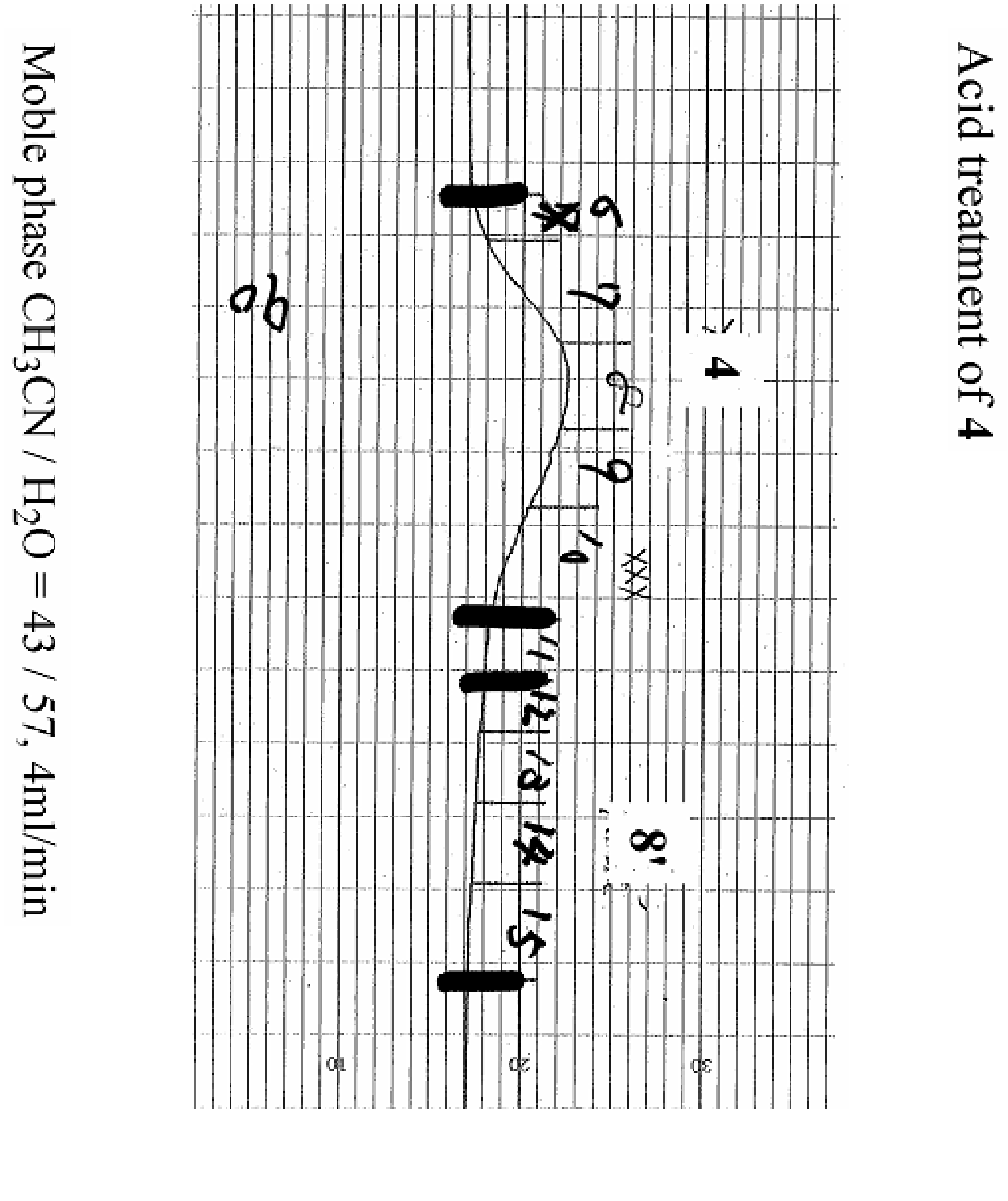

Figure S77

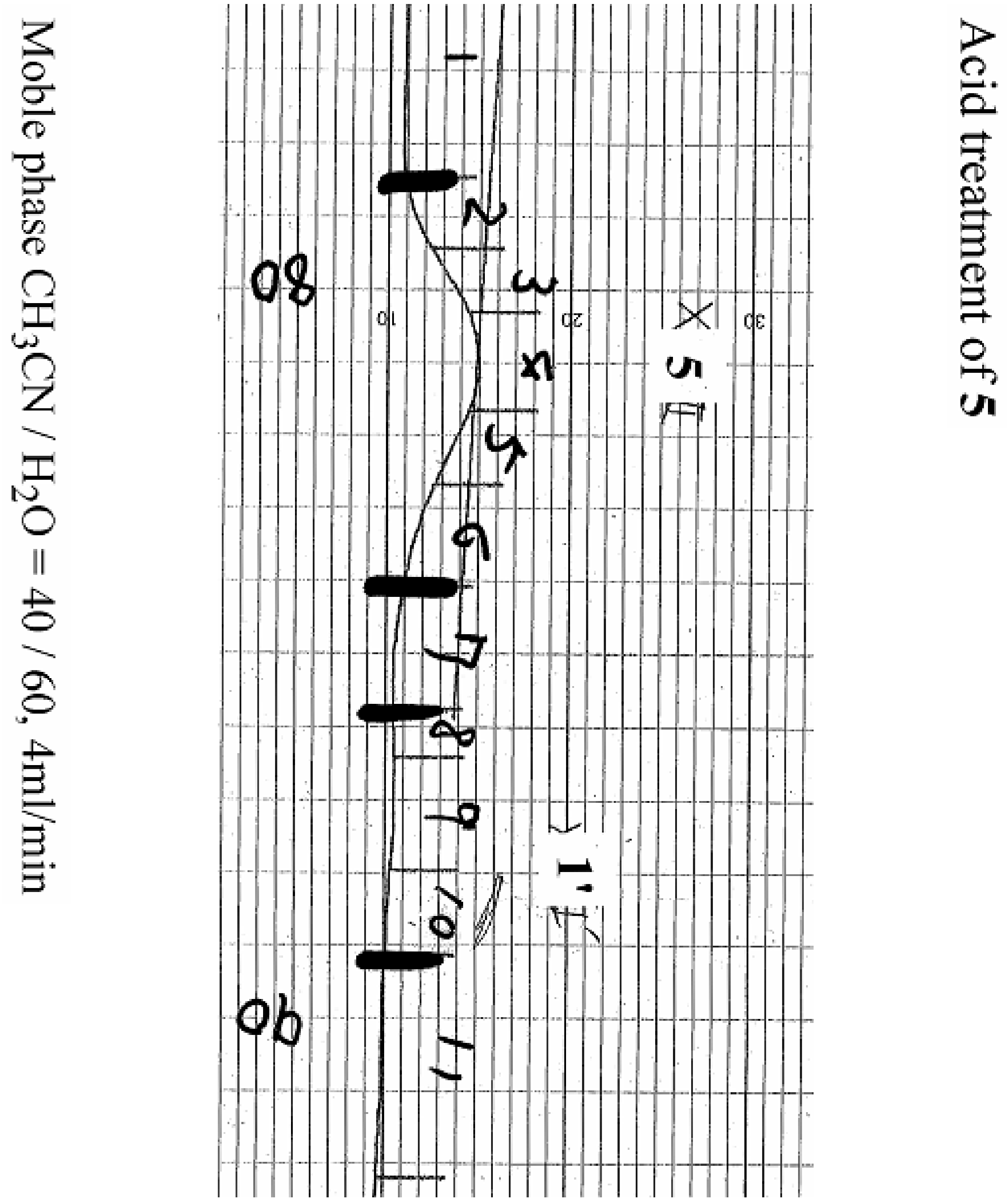

Figure S78

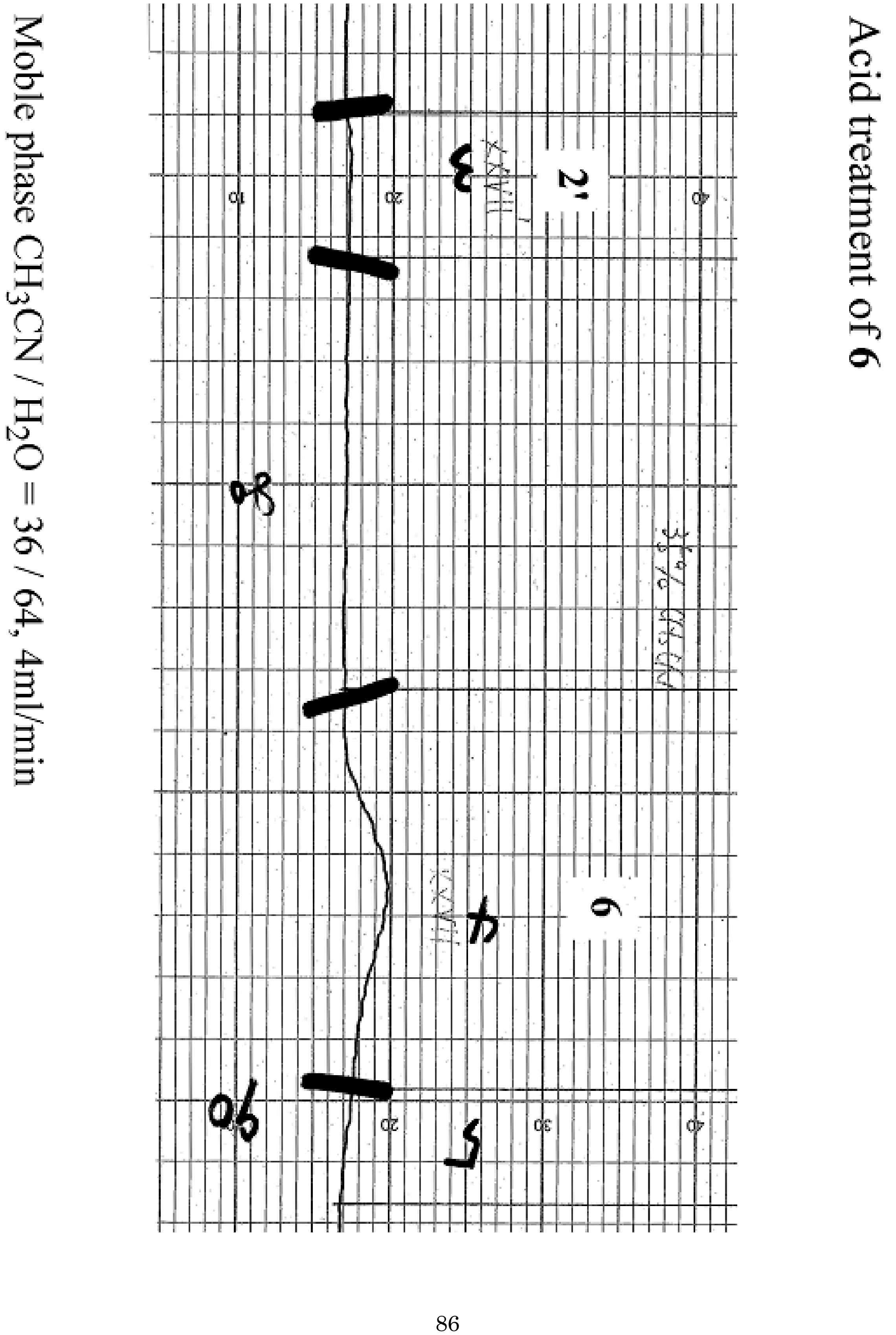

Figure S79

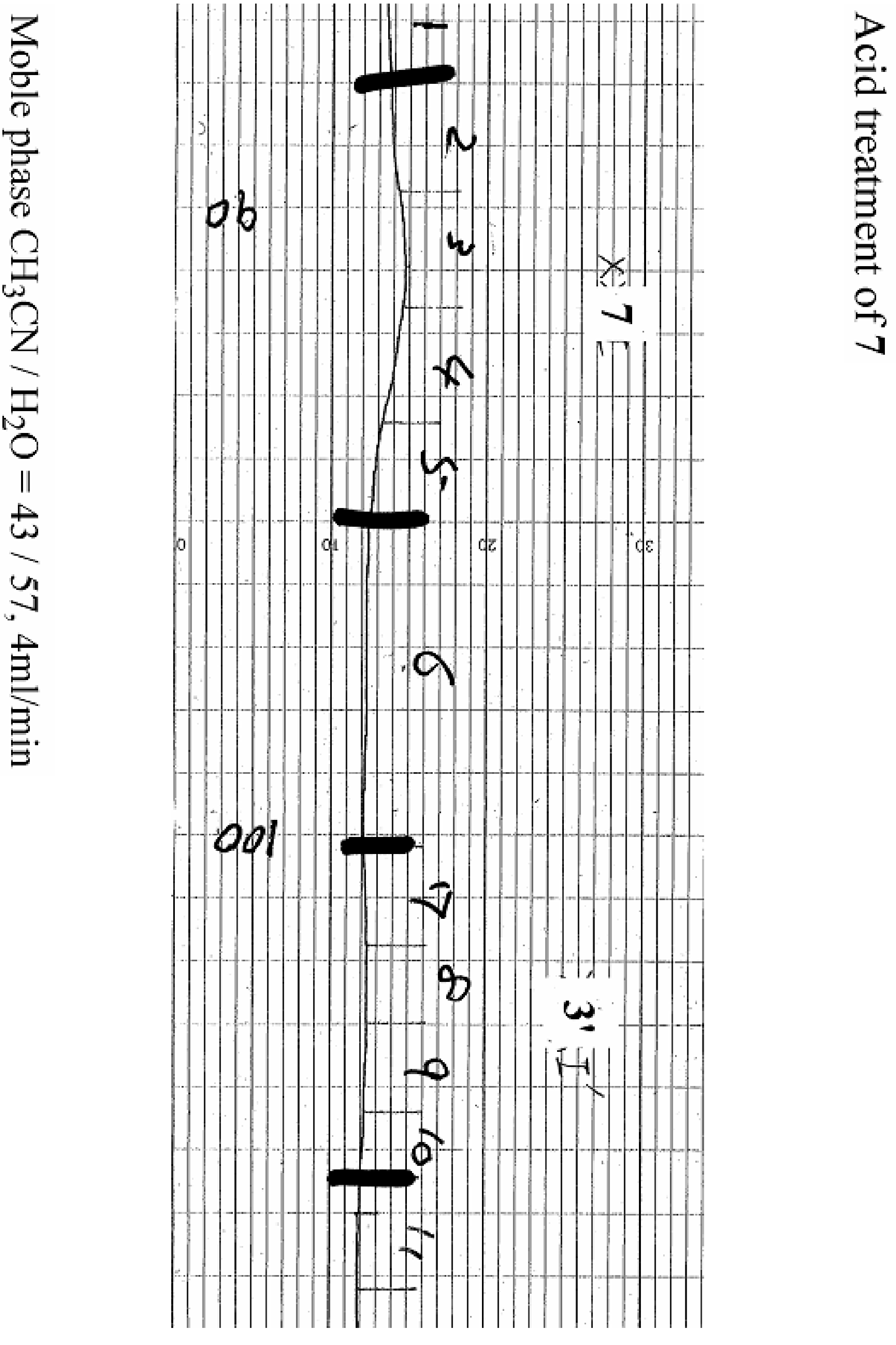

Figure S80

Acid treatment of 8

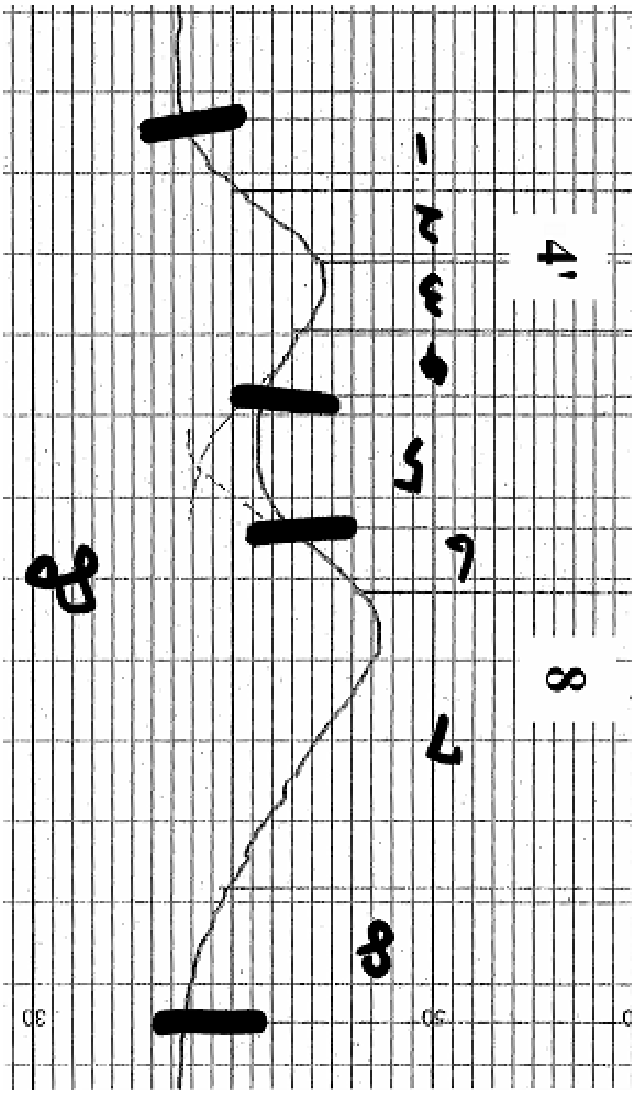

Moble phase CH_3CN / H_2O = 45 / 55, 4ml/min

Figure S81 the CD spectra of the 16 stereoisomers **1**–**8** and **1′**–**8′**, symmetrical Cotton effects between enantiomers

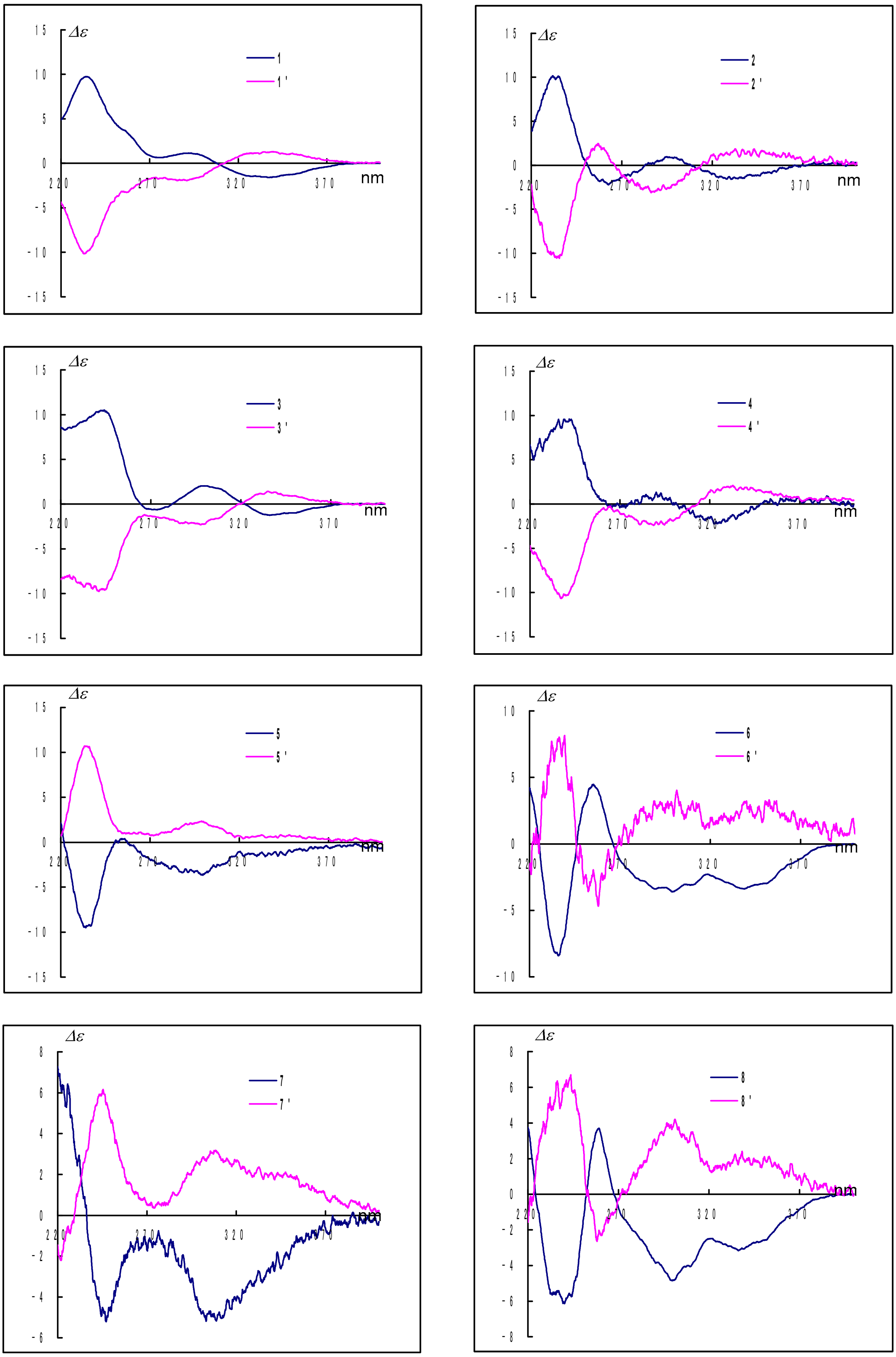

Supplement: Supplementary file 1 [file marinedrugs-16-00223-s001.pdf]
